# Revealing mind-body interaction mechanisms: a network meta-analysis of the effects of traditional Chinese medicine mind-body exercise interventions on stroke rehabilitation

**DOI:** 10.3389/fneur.2026.1759127

**Published:** 2026-03-13

**Authors:** Zhenhui Zhao, Xianliang Wang

**Affiliations:** School of Physical Education, Shandong University, Jinan, China

**Keywords:** activities of daily living, limb function, network meta-analysis, stroke, traditional Chinese medicine mind-body exercises

## Abstract

**Objective:**

This study employed a network meta-analysis to compare the efficacy of different Traditional Chinese Medicine mind-body exercises and determine their relative efficacy rankings in stroke rehabilitation.

**Methods:**

A comprehensive search of nine databases was conducted, covering studies from inception to February 2025. Participants included stroke patients (age ≥18 years). Interventions evaluated were four Traditional Chinese movement therapies: Tai Ji, Ba Duan Jin, Yi Jin Jing, and Wu Qin Xi. Primary outcomes included objective measures of upper limb function, lower limb function, balance function, and activities of daily living.

**Results:**

A total of 46 randomized controlled trials involving 3,096 stroke patients were included. Network meta-analysis revealed: (1) Upper limb function: Ba Duan Jin ranked highest (SMD = 1.33, 95% CI = 0.80–1.86). (2) Lower limb function: Ba Duan Jin was the most effective (SMD = 1.49, 95% CI = 0.97–2.01). (3) Balance function: Wu Qin Xi demonstrated the best efficacy (SMD = 1.56, 95% CI = 0.45–2.67). (4) Activities of daily living: Wu Qin Xi was the top-ranked intervention (SMD = 2.34, 95% CI = 0.91–3.76). While Tai Ji and Yi Jin Jing were not the optimal choices, they still exhibited significant therapeutic benefits compared to conventional therapies.

**Conclusion:**

Traditional Chinese Medicine mind-body exercises significantly improve limb function and activities of daily living in stroke patients. Ba Duan Jin is recommended for upper and lower limb rehabilitation, while Wu Qin Xi is optimal for activities of daily living restoration and balance recovery. These findings provide clinicians with evidence-based guidance for selecting interventions tailored to specific functional deficits.

**Systematic review registration:**

PROSPERO, identifier (CRD42025642353).

## 1 Introduction

Stroke is a severe, life-threatening acute cerebrovascular disease and a leading cause of chronic disability. Its core pathogenic mechanism involves ischemia and hypoxia in the affected brain tissue, which disrupts cellular energy homeostasis and impedes mitochondrial adenosine triphosphate (ATP) synthesis. This ultimately triggers a cascade of physiological responses, including altered vascular autoregulation and programmed cell death (1). In 2019, the global incidence of stroke reached 12.2 million cases, alongside 6.55 million stroke-related deaths, making it the second leading cause of death worldwide (2). In addition to its high mortality rate, the functional impairments faced by stroke survivors are equally severe. Driven by motor control and sensory deficits, up to 73% of patients experience falls within 6 months post-stroke. These incidents can lead to further secondary injuries and psychological barriers, thereby severely compromising their activities of daily living (ADL) and overall quality of life (3). Although passive interventions, such as pharmacological and surgical treatments, are indispensable in acute stroke management, their efficacy during the rehabilitation phase is limited, making it difficult to improve patients’ long-term functional outcomes. In contrast, physical activity, serving as an active rehabilitation strategy, has been well-established as a critical factor in promoting motor function remodeling in stroke patients (4).

As an active participation-based therapeutic modality, physical activity holds irreplaceable significance in stroke rehabilitation (5). Various exercise interventions have been utilized for the functional recovery of stroke survivors, yielding a proliferation of related trials, systematic reviews, and meta-analyses. Research indicates that regular exercise training promotes neural remodeling through multiple mechanisms: (1) Neurotrophic effects: Regular exercise elevates the levels of brain-derived neurotrophic factor (BDNF), thereby promoting synaptic plasticity and neurogenesis (6). (2) Cerebrovascular benefits: Exercise improves cerebral hemodynamics, enhances vascular recanalization rates, and mitigates secondary injuries (7). (3) Anti-inflammatory effects: Exercise modulates inflammatory mediators, reducing secondary neurodegeneration (8). These findings underscore the necessity of implementing personalized, evidence-based exercise interventions to optimize functional recovery and mitigate long-term disability (9).

Traditional rehabilitation approaches, such as constraint-induced movement therapy, often face limitations due to their high intensity and low patient adherence, while conventional aerobic exercise fails to fully address stroke patients’ specific needs for balance and coordination recovery. Traditional Chinese Medicine (TCM) mind-body exercises, such as Tai Ji (TJ), Ba Duan Jin (BDJ), Yi Jin Jing (YJJ), and Wu Qin Xi (WQX), as a system of physical therapy based on Chinese medical theory, have demonstrated unique potential in the field of neurological rehabilitation in recent years (10). Compared with modern exercise therapy, TCM mind-body exercises offer the following significant features and advantages: First, they emphasize the holistic view of “form-qi-shen” (body-energy-spirit), achieving synergistic training of the body and mind through slow, smooth movements combined with breath regulation and mental guidance (11). This unique training mode not only improves limb function but also regulates the autonomic nervous system to alleviate common post-stroke psychological issues, such as anxiety and depression (12, 13). Second, TCM mind-body exercises focus on the principle of “combining motion and stillness.” This approach effectively avoids the risk of secondary injuries associated with high-intensity training through low-intensity, continuous movements. Additionally, it activates the mirror neuron system via a multi-planar, complex movement pattern, thereby promoting the reorganization of the motor cortex (14, 15). In addition, the TCM mind-body exercise has significant cultural affinity and economy, which is easier for patients to accept and adhere to, and is not restricted by the venue and equipment, which is suitable for community and family rehabilitation (16). There are also studies confirming that TJ training significantly improves balance in the chronic phase (17), while BDJ training elevates upper limb Fugl-Meyer scores (18).

However, key limitations exist within the current body of evidence, notably the lack of direct, comparative data to establish an efficacy hierarchy among various interventions. Traditional meta-analyses are limited to comparing only two treatments at a time, making them inadequate for comprehensively evaluating trials with multiple treatment groups or answering critical questions about which treatment is superior. In contrast, network meta-analysis methods enable the simultaneous comparison of multiple treatments while accounting for other potential sources of heterogeneity. Therefore, in this study, we employed a systematic review combined with a network meta-analysis to integrate, for the first time, evidence from randomized controlled trials (RCT) regarding the effects of TCM mind-body exercises—such as TJ, BDJ, YJJ, and WQX—on limb function and ADL in stroke patients. By evaluating key outcome measures and quantifying the relative efficacy rankings of these interventions, this research aims to identify the optimal approaches for promoting functional recovery. Ultimately, these findings have the potential to provide a robust foundation for evidence-based decision-making in clinical practice and establish a methodological reference for modernizing research paradigms in traditional medicine (19).

## 2 Methods

### 2.1 Study registration

This systematic review adheres to the PRISMA-NMA (Preferred Reporting Items for Systematic Reviews and Meta-Analyses extension for network meta-analyses) guidelines for reporting and was prospectively registered in the PROSPERO International Prospective Register of Systematic Reviews (registration number: CRD 42025642353) (20).

### 2.2 Search strategy

To determine the effects of different exercise types on stroke patients, we comprehensively searched nine electronic databases: PubMed, Web of Science, Embase, Scopus, The Cochrane Library, China National Knowledge Infrastructure (CNKI), Wanfang Data, the Chinese Biomedical Literature Database, and the VIP Database. The search spanned from database inception to February 10, 2025. The search strategy utilized a combination of subject headings and free-text terms. Additionally, we manually screened the reference lists of previously published relevant studies to ensure comprehensive literature coverage. Supplementary Table 2 details the specific search strategy for each database.

### 2.3 Study selection

#### 2.3.1 Inclusion criteria

Studies that met the following criteria were included according to the PICOS framework: (1) P: patients diagnosed with stroke (hemorrhagic or ischemic), and aged ≥ 18 years; (2) I: the intervention was one of TJ, BDJ, YJJ, or WQX, with no limit on the number of groups, and at least one group receiving a single exercise intervention; (3) C: conventional therapy (CT) or an exercise intervention different from the intervention group; (4) O: the primary outcome measures were FMA, FMA-LE, FMA-UE, BBS, BI, and MBI; and (5) S: only randomized controlled trials were included.

#### 2.3.2 Exclusion criteria

Studies were excluded if they met any of the following criteria: (1) use of an intervention other than the exercise modalities listed above, or a combination of exercise interventions; (2) duplicate publications, unavailable full texts, incomplete or missing data, or study protocols; (3) non-randomized controlled trials; (4) conference abstracts or systematic reviews; and (5) problematic trial designs.

### 2.4 Literature selection and data extraction

We managed the retrieved records by importing them into EndNote 21. Two reviewers independently screened the titles and abstracts to determine whether the articles met the inclusion criteria. Each study was critically assessed against these criteria, and discrepancies were resolved through consensus or by consulting a third reviewer. Information extracted included: study characteristics (first author, year of publication); population characteristics (whether participants were stroke patients, sample size, age, sex ratio); details of the intervention and control (intervention modality, type of control, duration, and frequency of treatment); and outcome measures, along with the corresponding data.

### 2.5 Risk of bias assessment

During data extraction, the Cochrane Collaboration’s tool was used to assess the risk of bias in the included studies. This assessment was performed independently by two reviewers, with any discrepancies resolved through consensus or by consulting a third reviewer.

### 2.6 Data analysis

#### 2.6.1 Standard meta-analysis

Stata software, versions 15 and 18 (StataCorp LLC, College Station, TX, United States), was utilized to perform the meta-analysis and generate forest plots. Given that the dataset comprised continuous variables, the standardized mean difference (SMD) was selected as the effect size metric. Between-study heterogeneity was evaluated using the I^2^ statistic: an I^2^ ≥ 50% was interpreted as substantial heterogeneity, necessitating the application of a random-effects model; conversely, an I^2^ < 50% indicated low heterogeneity, warranting the use of a fixed-effects model. Statistical significance was set at *p* < 0.05.

#### 2.6.2 Network meta-analysis

A network meta-analysis was conducted using Stata software, versions 15 and 18, utilizing dedicated analytical and visualization packages (21). A consistency model within the frequentist framework was employed to analyze the data. A network graph was constructed to map all interventions, where node size represented the total sample size for each intervention, and line thickness indicated the number of studies directly comparing the connected interventions. The efficacy rankings of the different TCM mind-body exercise techniques were estimated using the surface under the cumulative ranking curve (SUCRA). SUCRA values range from 0 to 100%, with 100% indicating the most effective treatment and lower values indicating decreased efficacy (22).

#### 2.6.3 Additional analysis

Review Manager 5.4 software was used to assess the risk of bias in the included studies. To examine potential sources of variability in limb function and ADL outcomes, subgroup analyses were conducted, stratified by moderator variables including intervention cycle, exercise frequency, and patient age. A sensitivity analysis was performed using the leave-one-out approach to examine the robustness of the results. The likelihood of publication bias was initially assessed via visual inspection of funnel plots. If asymmetry was observed, egger’s test was further performed to quantify the bias; *p* > 0.05 indicated no significant publication bias.

## 3 Results

### 3.1 Literature screening process and outcomes

**Figure 1:**
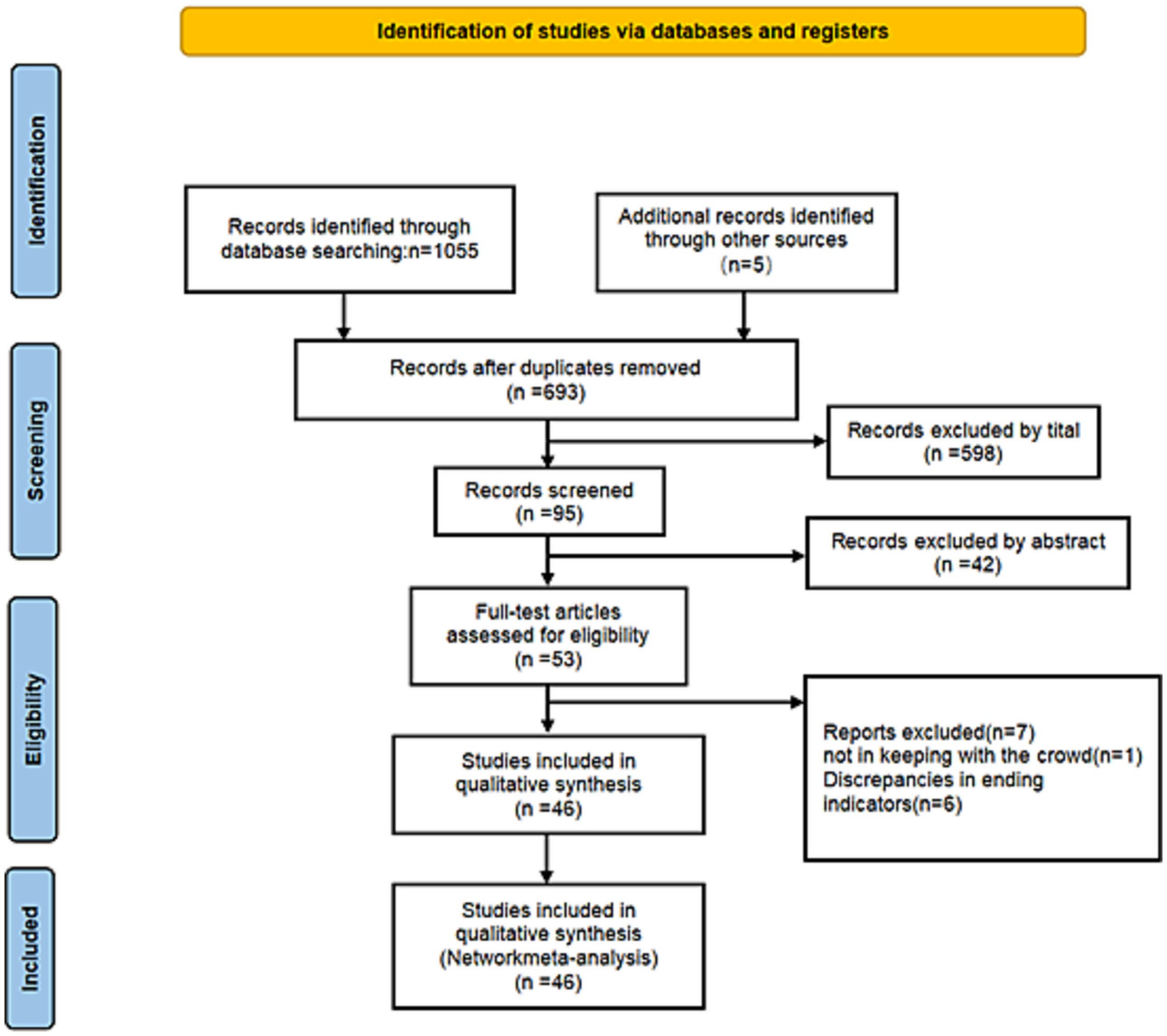
PRISMA flowchart of included studies.

The initial electronic search identified 1,060 potentially relevant publications. After removing 367 duplicates, 640 records were excluded by screening titles and abstracts. Of the remaining 53 studies eligible for full-text evaluation, 7 were excluded due to ineligible populations and unrelated outcomes. Finally, 46 RCT were included in the meta-analysis (Figure 1).

### 3.2 Characteristics of included studies

The 46 included RCT were published between 2010 and 2024, comprising a total of 3,096 individuals with stroke. Individual sample sizes ranged from 17 to 244. Intervention duration ranged from 3 to 24 weeks. A total of four TCM mind-body exercises were evaluated: TJ (18 studies), BDJ (16 studies), YJJ (8 studies), and WQX (4 studies) (Supplementary Table 5).

### 3.3 Risk of bias assessment

**Figure 2:**
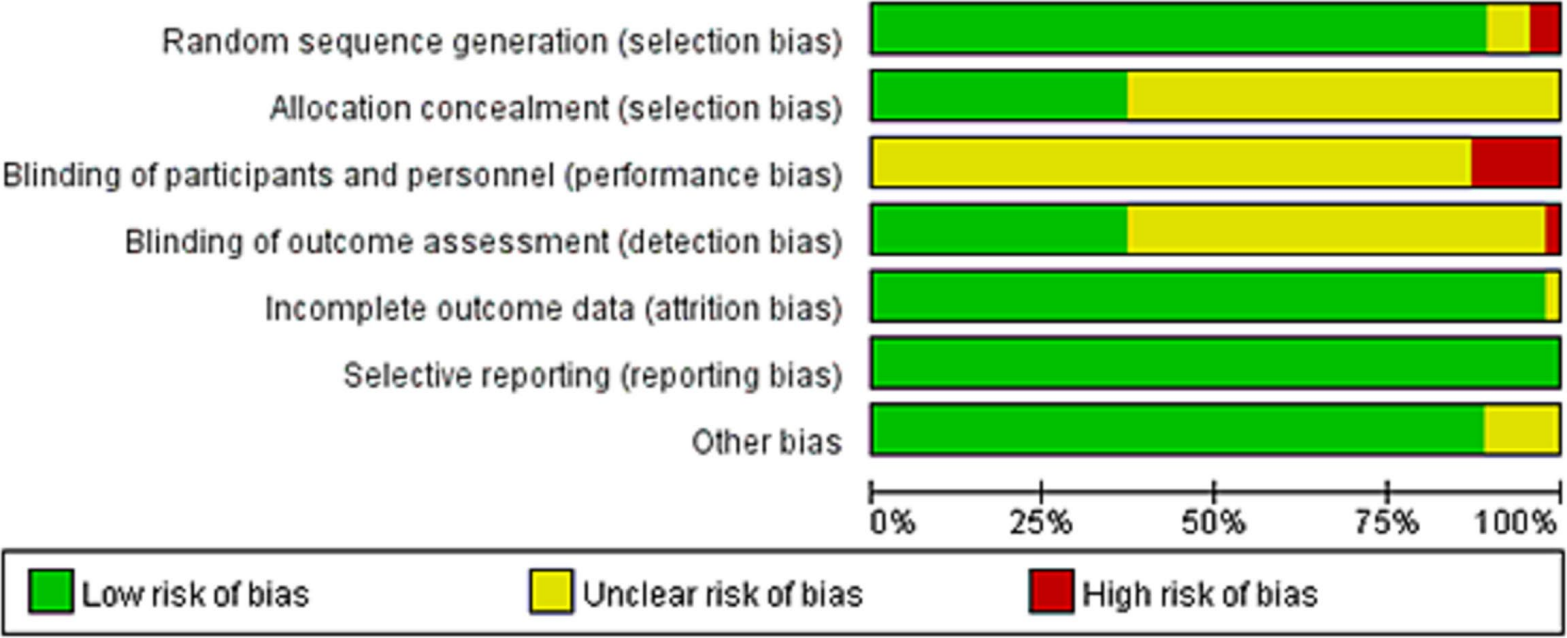
Risk of bias graph.

Two review authors employed the Cochrane risk of bias tool, which encompasses seven domains: random sequence generation, allocation concealment, blinding of participants, blinding of outcome assessment, incomplete outcome data, selective reporting, and other sources of bias. The potential risk of bias in each domain was categorized as “low risk,” “high risk,” or “unclear risk.” Any disagreements between the two reviewers were resolved by consulting a third author to reach a consensus. (1) The methodological quality of the 46 included studies was evaluated using RevMan 5.4. Overall, the included studies demonstrated a relatively low risk of bias. (2) Based on the Cochrane criteria, the quality assessment revealed the following: 41 studies reported adequate random sequence generation, 3 had unclear randomization methods, and 2 utilized incorrect randomization procedures; 17 studies reported specific allocation concealment methods, while 29 had unclear allocation concealment; and a total of 17 studies implemented blinding of outcome assessment. Furthermore, the majority of the studies were rated as low risk regarding attrition bias (incomplete outcome data), reporting bias (selective reporting), and other biases (Figure 2; Supplementary Figure 3).

### 3.4 Standard meta-analysis

#### 3.4.1 Upper limb function

**Figure 3:**
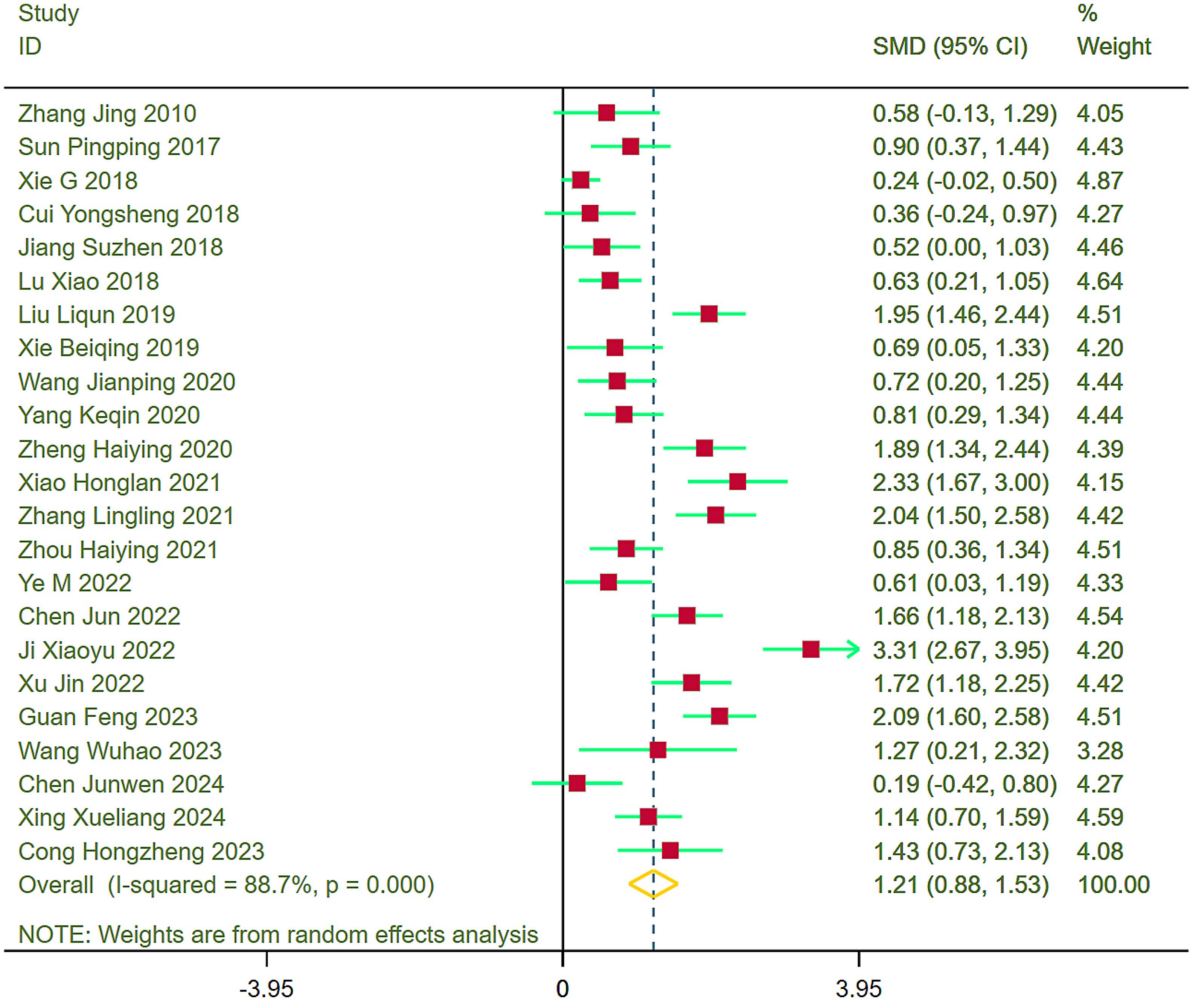
Comparison of upper limb function between the experimental group and the control group.

Forest plots were generated to assess upper limb function across the included studies, revealing heterogeneity (I^2^ = 88.7%). Consequently, a random-effects model was implemented for the meta-analysis. Results indicated that stroke patients in the exercise group demonstrated significantly better recovery of upper limb function compared to those in the control group (SMD = 1.21, 95% CI = 0.88–1.53) (Figure 3).

#### 3.4.2 Lower limb function

**Figure 4:**
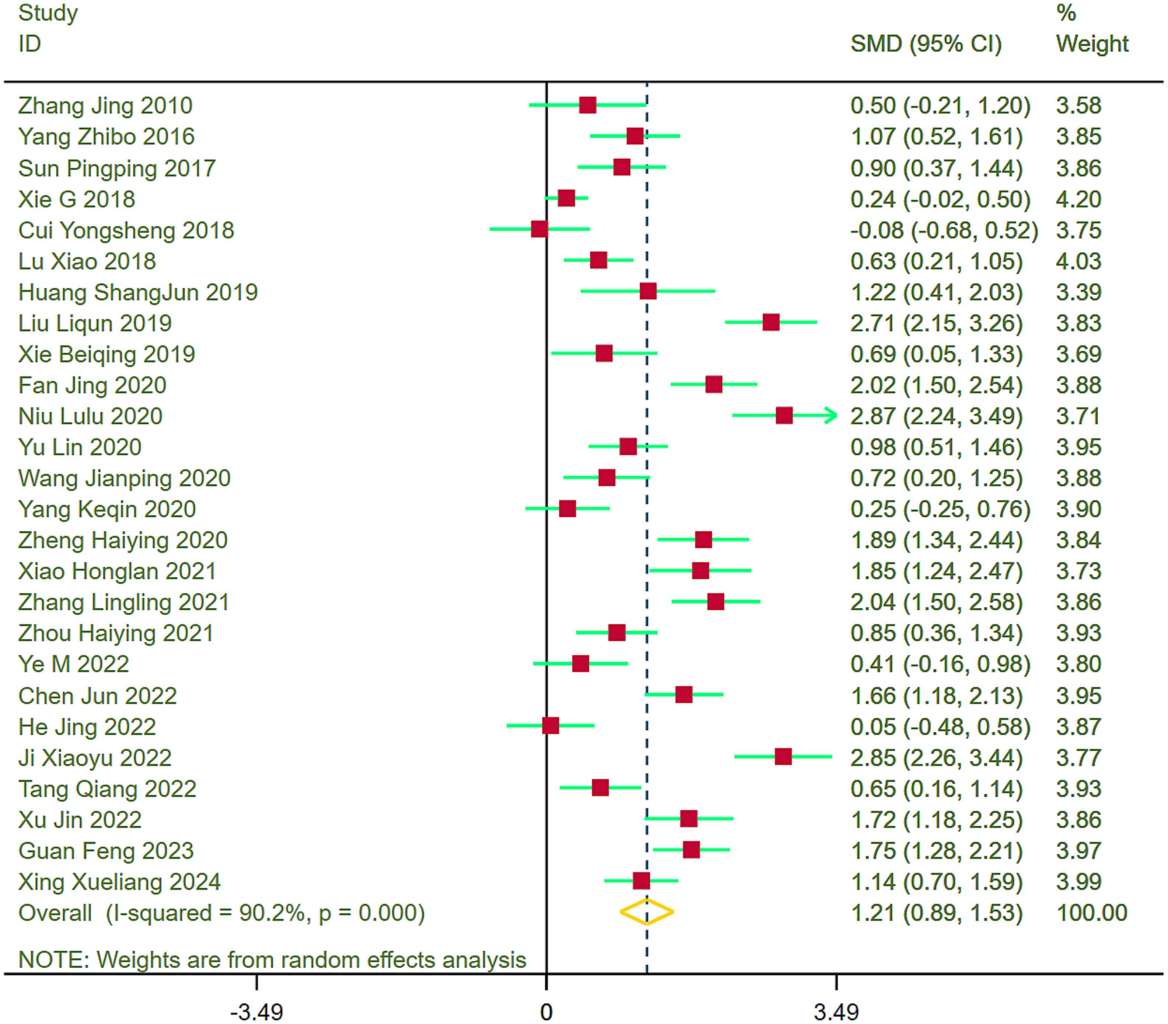
Comparison of lower limb function between the experimental group and the control group.

Forest plots were constructed to evaluate lower limb function across the included studies, indicating heterogeneity (I^2^ = 90.2%). Thus, a random-effects model was applied for the meta-analysis. Results demonstrated that stroke patients in the exercise group exhibited notably superior recovery of lower limb function compared to the control group (SMD = 1.21, 95% CI = 0.89–1.53) (Figure 4).

#### 3.4.3 Balance function

**Figure 5:**
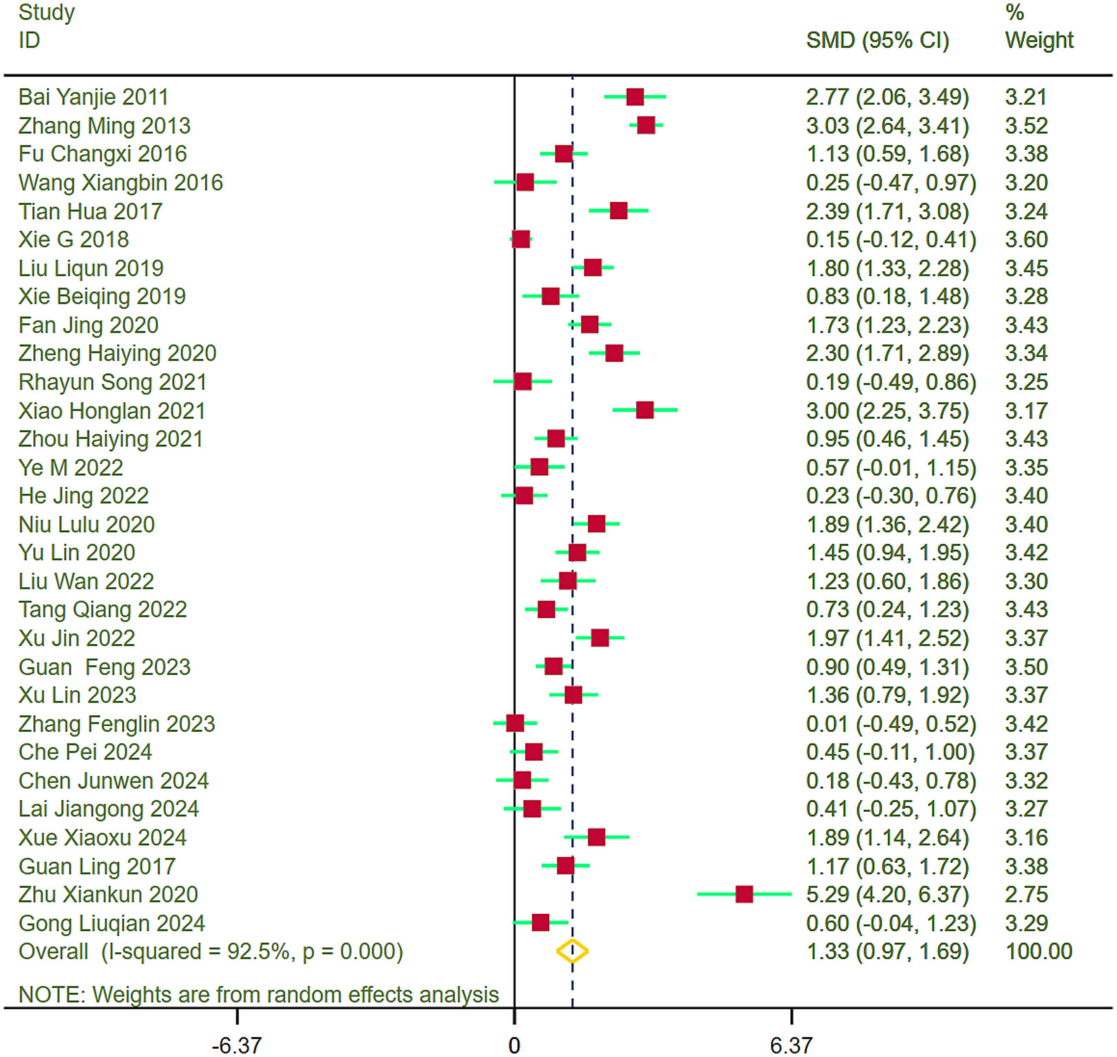
Comparison of balance function between the experimental group and the control group.

Forest plots were generated to evaluate balance function across the included studies, unveiling heterogeneity (I^2^ = 92.5%). Subsequently, a random-effects model was adopted for the meta-analysis. Outcomes revealed that stroke patients in the exercise group demonstrated substantially better balance function recovery than those in the control group (SMD = 1.33, 95% CI = 0.97–1.69) (Figure 5).

#### 3.4.4 ADL

**Figure 6:**
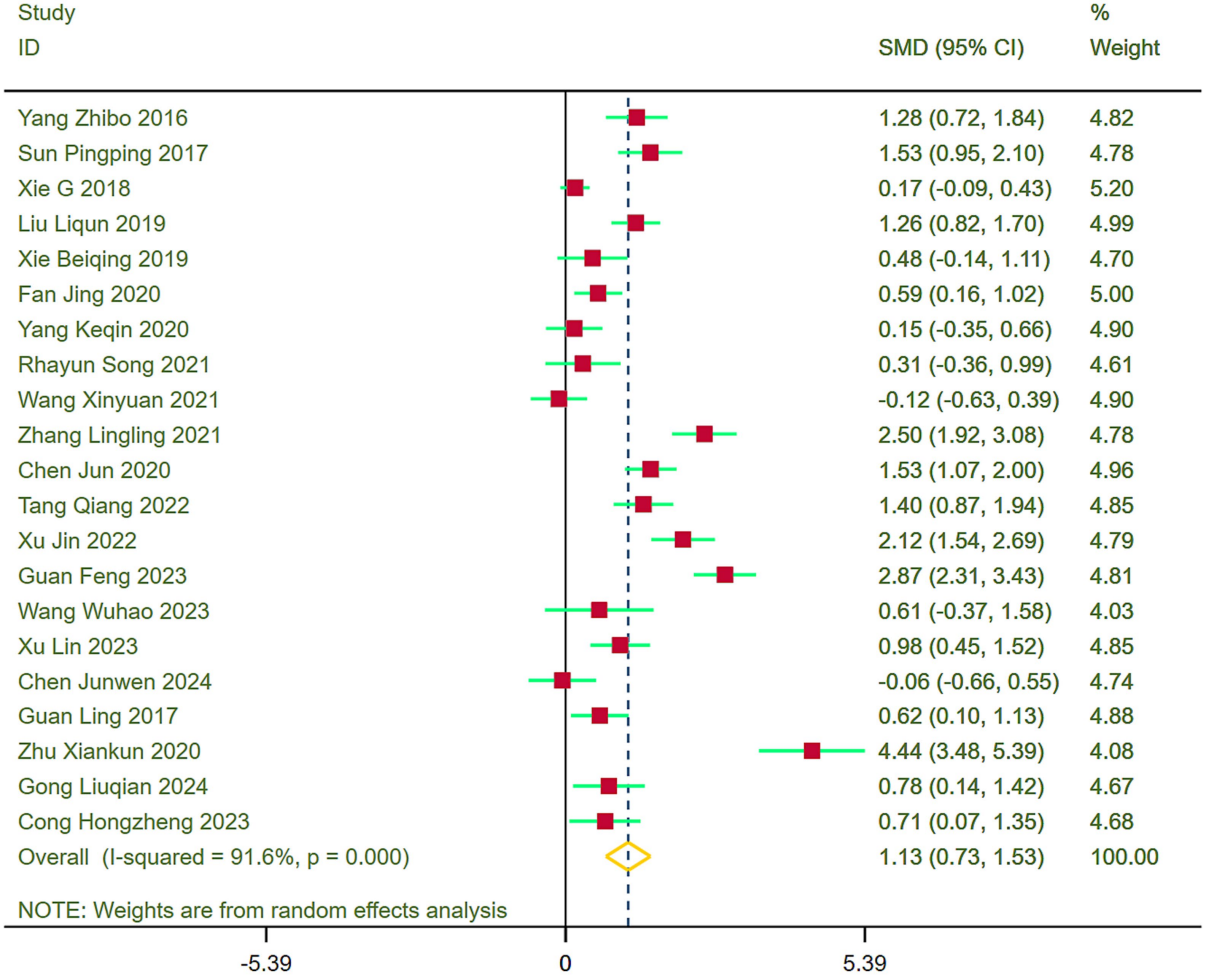
Comparison of ADL between the experimental group and the control group.

Forest plots were formulated to evaluate ADL across the included studies, disclosing heterogeneity (I^2^ = 91.6%). Thereupon, a random-effects model was deployed for the meta-analysis. Results established that stroke patients in the exercise group exhibited markedly enhanced ADL recovery compared to those in the control group (SMD = 1.13, 95% CI = 0.73–1.53) (Figure 6).

### 3.5 Network evidence plots

**Figure 7:**
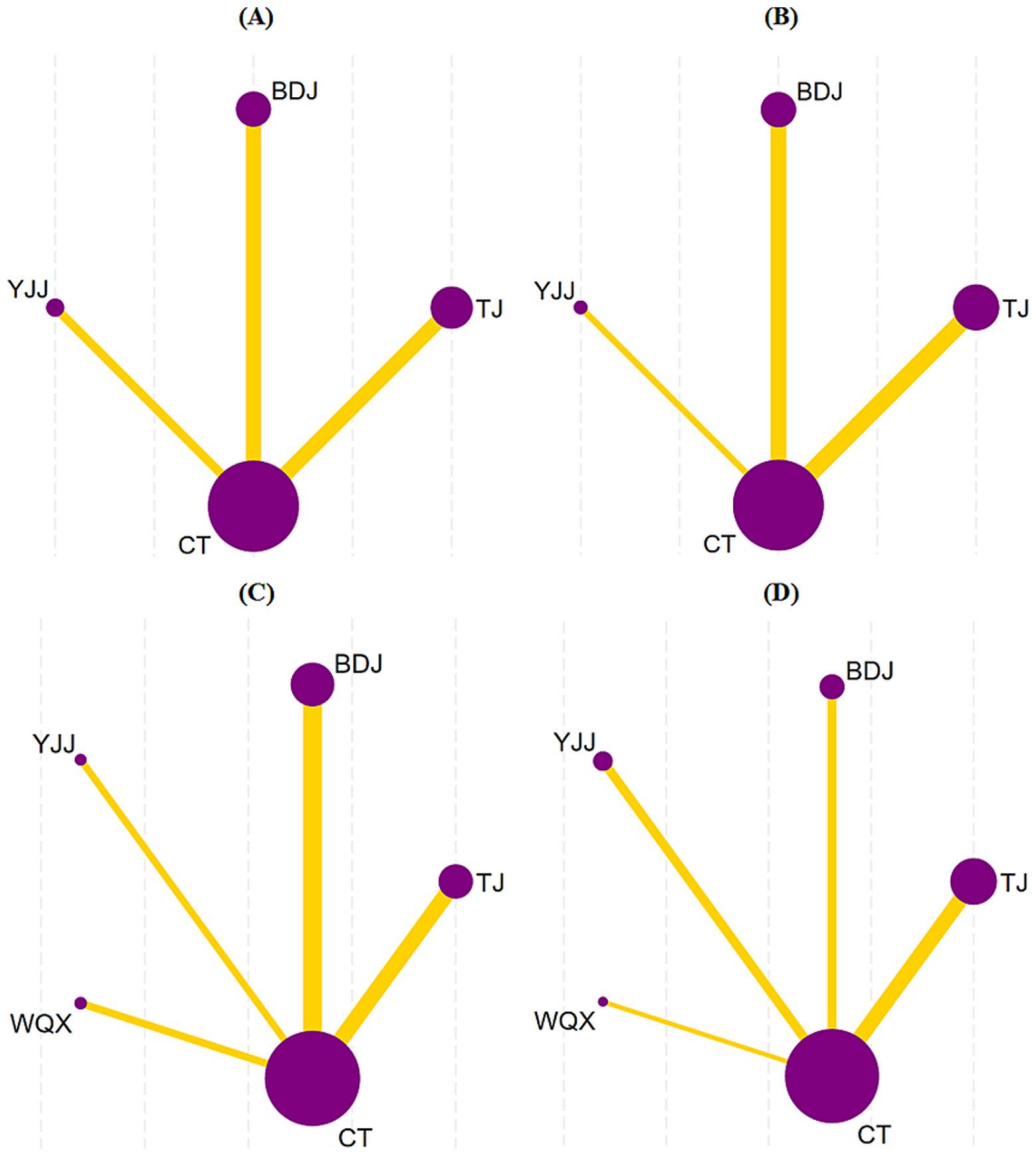
Reticulated evidence map. **(A)** Upper limb function, **(B)** Lower limb function, **(C)** balance function, **(D)** ADL.

The network structures for the respective outcomes were as follows: 23 studies evaluated upper limb functional rehabilitation, encompassing three types of TCM mind-body exercises (TJ, BDJ, and YJJ) and CT; 26 studies assessed lower limb functional rehabilitation involving the same three exercises and CT; 30 studies analyzed balance function, incorporating four TCM mind-body exercises (TJ, BDJ, YJJ, and WQX) and CT; and 21 studies evaluated the recovery of ADL, also involving all four exercises and CT. The network evidence diagram visualizes the comparisons among the different interventions. In these plots, the nodes represent the individual therapies, and the connecting lines between them indicate direct comparisons. The absence of a line signifies a lack of direct comparison, indicating that relative efficacy is estimated through indirect comparisons. Furthermore, the thickness of each line corresponds to the number of studies directly comparing the two connected therapies, while the size of each node reflects the total sample size of individuals receiving that specific intervention (Figure 7).

### 3.6 Inconsistency testing

This study was an indirect comparison of TCM mind-body exercise on upper limb function, lower limb function, balance function, and ADL in stroke patients, and the reticulated evidence relationship diagrams all met the criteria for determining acyclic networks without the need for consistency tests.

### 3.7 Network meta-analysis

**Figure 8:**
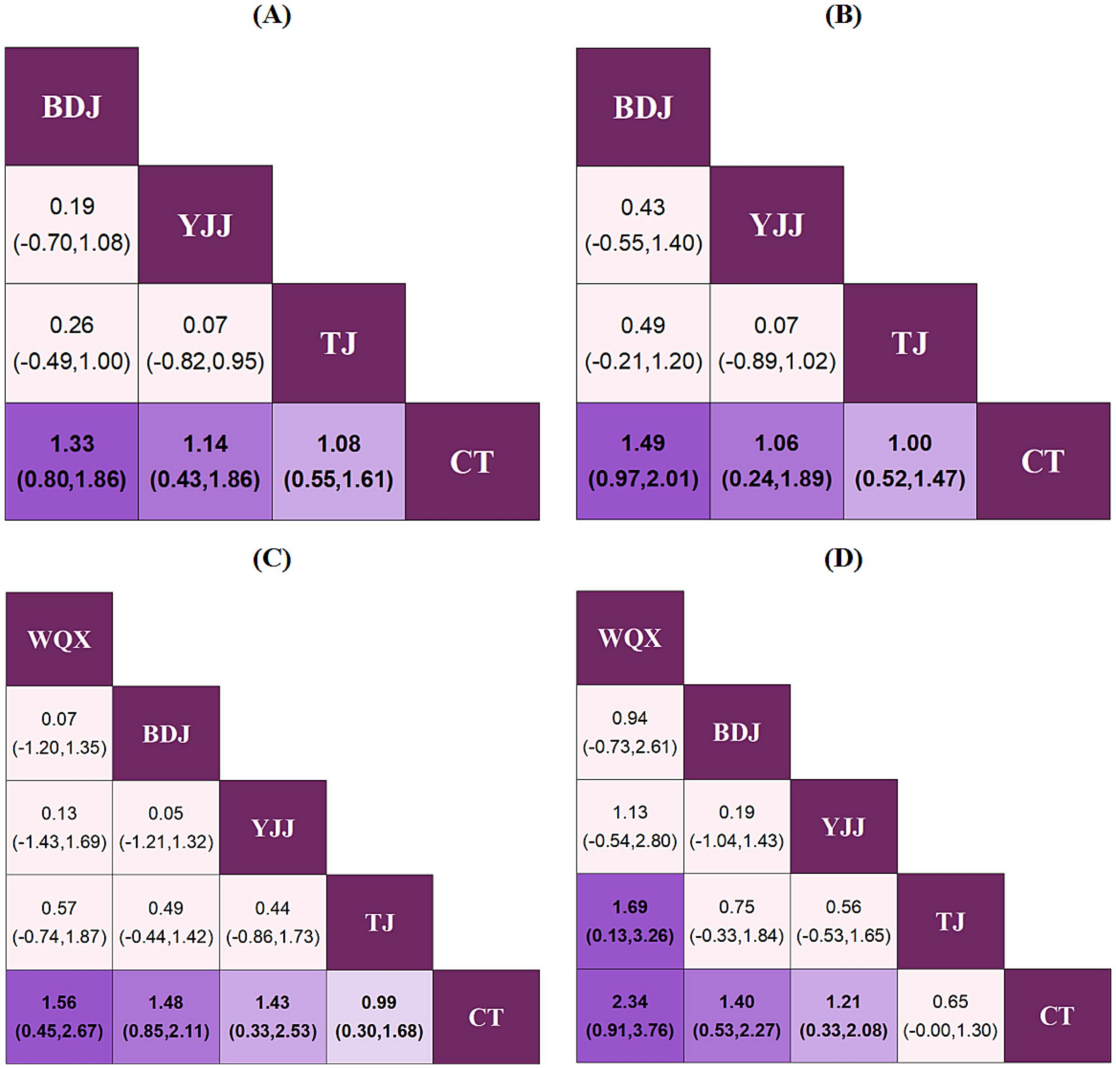
League diagrams under each outcome indicator. **(A)** Upper limb function, **(B)** lower limb function, **(C)** Balance function, **(D)** ADL.

**Figure 9:**
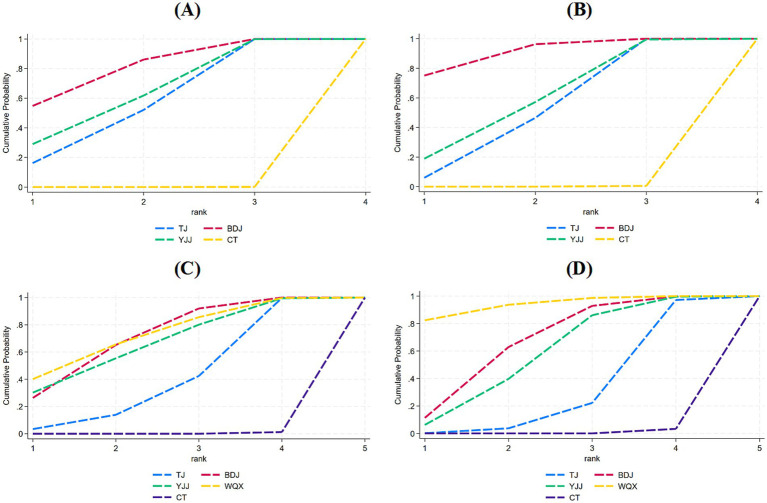
Surface under the cumulative ranking curve values for different interventions **(A–D)**. **(A)** Upper limb function; **(B)** Lower limb function; **(C)** Balance function; and **(D)** ADL.

Regarding upper limb function, BDJ (SMD = 1.33, 95% CI = 0.80–1.86), YJJ (SMD = 1.14, 95% CI = 0.43–1.86), and TJ (SMD = 1.08, 95% CI = 0.55–1.61) all demonstrated significantly superior efficacy compared to CT (*p* < 0.05). The SUCRA probability ranking indicated that BDJ was the optimal therapy: BDJ (80.3%) > YJJ (63.6%) > TJ (56.1%) > CT (0.0%) (Figures 8A, 9A; Supplementary Figure 20).

Similarly, for lower limb function, BDJ (SMD = 1.49, 95% CI = 0.97–2.01), YJJ (SMD = 1.06, 95% CI = 0.24–1.89), and TJ (SMD = 1.00, 95% CI = 0.52–1.47) all exhibited significantly superior efficacy compared to CT (*p* < 0.05). The SUCRA ranking revealed BDJ as the most effective intervention: BDJ (90.5%) > YJJ (58.5%) > TJ (50.8%) > CT (0.2%) (Figures 8B, 9B; Supplementary Figure 20).

In terms of balance function, WQX (SMD = 1.56, 95% CI = 0.45–2.67), BDJ (SMD = 1.48, 95% CI = 0.85–2.11), YJJ (SMD = 1.43, 95% CI = 0.33–2.53), and TJ (SMD = 0.99, 95% CI = 0.30–1.68) all showed significantly superior efficacy over CT (*p* < 0.05). According to the comprehensive SUCRA ranking, WQX emerged as the potentially optimal therapy: WQX (72.8%) > BDJ (70.8%) > YJJ (66.3%) > TJ (39.9%) > CT (0.3%) (Figures 8C, 9C; Supplementary Figure 20).

Finally, concerning ADL, WQX (SMD = 2.34, 95% CI = 0.91–3.76), BDJ (SMD = 1.40, 95% CI = 0.53–2.27), and YJJ (SMD = 1.21, 95% CI = 0.33–2.08) all demonstrated significantly superior efficacy compared to CT. The SUCRA ranking indicated that WQX was the most effective intervention for ADL: WQX (93.7%) > BDJ (66.7%) > YJJ (57.9%) > TJ (30.8%) > CT (0.8%) (Figures 8D, 9D; Supplementary Figure 20).

### 3.8 Subgroup analyses of moderator variables

Subgroup analyses of four outcome measures were performed by stratifying with moderator variables: Intervention cycle, exercise frequency, and patient age (Supplementary Table 4). Results indicated that when categorized by intervention cycle (≤4 weeks, 5–8 weeks, >8 weeks), moderate cycles (5–8 weeks) yielded the most favorable outcomes for upper limb function and balance function; extended cycles (>8 weeks) demonstrated optimal therapeutic effects for lower limb function; and short cycles (≤4 weeks) showed the highest efficacy for ADL.

Stratified into two subgroups by exercise frequency (≤three times a week, >three times a week), the frequency associated with optimal efficacy was consistently >three times a week for upper limb function, lower limb function, balance function, and ADL.

Stratified into two subgroups by patient age (<60 years old, ≥60 years old), TCM mind-body exercise demonstrated the most pronounced therapeutic effects for upper limb function, lower limb function, and ADL in patients aged ≥60 years old; conversely, it yielded optimal efficacy for balance function in patients aged <60 years old.

### 3.9 Publication bias

**Figure 10:**
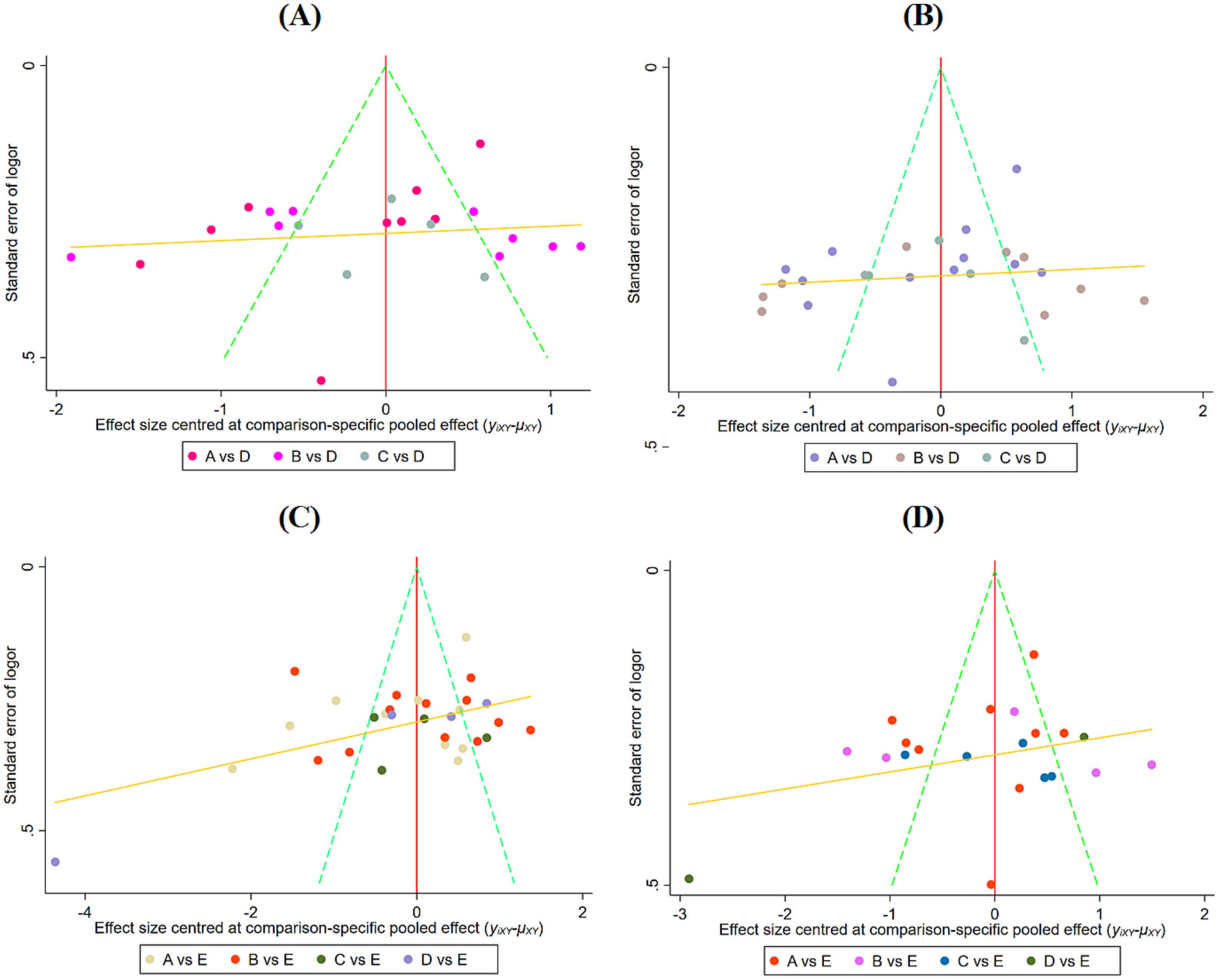
Publication bias test for different outcome indicators. **(A)** Upper limb function; **(B)** Lower limb function; **(C)** Balance function; and **(D)** ADL.

Funnel plots were generated for the visual inspection of the four outcome measures. The results indicated that while most study points clustered in the upper-middle region of the plot, they did not exhibit a perfectly symmetric distribution pattern centered around the pooled effect size (Figure 10). Subsequently, egger’s test was conducted, revealing no significant publication bias across any of the four outcome measures (*p* > 0.05) (Supplementary Table 3).

### 3.10 Sensitivity analysis

Sensitivity analyses were performed using a leave-one-out approach for each of the four outcome measures. The results demonstrated that the exclusion of any single study did not significantly alter the pooled effect size estimates, and all recalculated point estimates remained within the 95% confidence intervals of the original pooled effect sizes. These results confirm that the findings of the network meta-analysis are highly robust (Supplementary Figures 21–24).

## 4 Discussion

This study included a total of 46 RCT. Meta-analysis findings demonstrated that TCM mind-body exercises significantly improved limb function and ADL in stroke patients. Network meta-analysis revealed that three TCM mind-body exercises (BDJ, YJJ, and TJ) significantly optimized both upper and lower limb functional outcomes, with BDJ emerging as the optimal therapy for both. Four exercises (WQX, BDJ, YJJ, and TJ) significantly improved balance function, with WQX being the potentially optimal choice. Additionally, three exercises (WQX, BDJ, and YJJ) significantly enhanced ADL, with WQX likely being the most effective. Subgroup analyses based on moderator variables indicated that for upper limb and balance functions, exercises performed >3 times/week and sustained for 5–8 weeks yielded superior therapeutic effects. For lower limb function, interventions conducted >3 times/week for >8 weeks produced more favorable outcomes. Regarding ADL, exercises implemented >3 times/week for ≤4 weeks generated optimal efficacy. Furthermore, TCM mind-body exercises exhibited enhanced efficacy in restoring upper and lower limb function, as well as ADL, among patients aged ≥60 years, whereas they provided more clinically meaningful benefits for balance function in patients aged <60 years.

### 4.1 Comparison with other related studies

TCM mind-body exercises (e.g., TJ, BDJ, YJJ, and WQX) have been practiced for thousands of years (23). Current evidence shows that neurorehabilitation interventions, particularly TJ and BDJ, provide significant clinical benefits in improving static and dynamic postural control after stroke (18, 24). Although existing studies have established the efficacy of TCM mind-body exercises in stroke rehabilitation, methodological limitations remain in constructing a multidimensional efficacy assessment system. Specifically, there is a lack of systematic comparative studies utilizing network meta-analysis across four critical dimensions of post-stroke functional reconstruction (upper limb function, lower limb function, balance function, and ADL), leaving the optimal intervention modalities unclear.

Existing meta-analyses have primarily focused on the effects of various TCM mind-body exercises on chronic heart failure and blood pressure regulation (25, 26), or on interventions involving a single modality (e.g., TJ) (27). Significant gaps remain in determining the optimal TCM mind-body exercise protocols for rehabilitating upper limb, lower limb, and balance functions, as well as improving ADL in stroke patients. Using a network meta-analysis approach, we systematically integrated evidence from RCT on TCM mind-body exercises (such as TJ, BDJ, YJJ, and WQX) targeting limb function and ADL in stroke patients. By constructing a multiple-treatment comparison network, we quantitatively ranked their relative efficacies. This study not only provides high-quality evidence for developing clinical exercise strategies in traditional Chinese medicine but also helps establish a methodological paradigm for modernizing traditional medicine research.

### 4.2 Mechanistic insights into key findings

The results of this study indicate that BDJ ranked as the most effective intervention for improving both upper and lower limb motor function in stroke patients. As a TCM mind-body exercise originating from the Northern Song Dynasty, BDJ is characterized by its core philosophy of “balancing movement with stillness and firmness with flexibility”. Its therapeutic mechanism emphasizes the deep integration of “mind, breath, and posture”—specifically, the synergistic effects of body adjustment (rhythmic limb control), breath adjustment (respiratory regulation), and mind adjustment (conscious mental guidance) to facilitate the systematic recovery of neurological function (28). From a biomechanical perspective, BDJ encompasses multi-dimensional upper limb rotations, cross-joint synergistic flexion and extension, and core trunk engagement. This whole-body kinetic chain model offers significant advantages for reconstructing motor control in the upper and lower limbs of stroke patients, a finding highly consistent with the research of Zou et al. (29) and Yuen et al. (30). Furthermore, from a physiological standpoint, BDJ serves as a gentle aerobic exercise. Its slow muscle stretching and controlled rhythmic breathing not only effectively improve local cerebral blood flow perfusion but also upregulate the expression of brain-derived neurotrophic factor (BDNF). This optimization of the biochemical environment further induces significant neuroplastic changes, ultimately accelerating the compensation and repair of damaged motor nerve conduction pathways (31).

As a typical TCM mind-body exercise, WQX demonstrates significant advantages in improving ADL and balance in stroke patients. Biomechanically, by imitating the whole-body, multi-axial movements of five animal forms and incorporating specific mental guidance, this exercise effectively enhances the body’s proprioceptive feedback (32). Its multi-postural and multi-modal training pattern not only improves muscle strength in the upper and lower extremities and fine motor skills in the fingers, but also enhances spinal core stability. This provides a solid mechanical foundation for the recovery of patients’ balance and daily activity capabilities, which is corroborated by the findings of Yu et al. (33). Neurophysiologically, WQX integrates breath control with mind-body regulation. As a low-to-moderate-intensity aerobic intervention, its multi-planar, complex movement stimuli effectively induce the remodeling of the peri-infarct motor cortex and distal neural networks. This alters neuronal synaptic morphology and dendritic length, thereby enhancing the structural plasticity of the nervous system and ultimately accelerating the restoration of impaired motor functions (34). Furthermore, regular WQX practice can effectively regulate blood pressure levels, further reducing the risk of recurrent cardiovascular and cerebrovascular events in these patients.

Although TJ and YJJ did not achieve the highest rankings for improving upper limb function, lower limb function, balance function, and ADL in stroke patients, they still showed significant positive modulatory effects on the relevant outcome measures by virtue of the neuromodulatory mechanisms of multimodal movement patterns, multisensory inputs, and multisystemic integration, which is in line with the results of the latest study by Luo et al. (35).

### 4.3 Clinical and scientific significance

The study advocates for the introduction of convenient, economical, and well-established TCM mind-body exercises into the field of limb function and ADL rehabilitation for stroke survivors, as well as the development of targeted implementation programs based on specific areas of dysfunction. Specifically, BDJ is preferred based on its SUCRA rankings for improving upper and lower limb function; WQX is recommended for improving ADL and rehabilitating balance dysfunction; and, given the beneficial effects of TJ and YJJ, they should be integrated into comprehensive TCM mind-body exercise programs to provide a new paradigm for the modernization of traditional medicine.

### 4.4 Strengths and limitations

This study is characterized by its use of network meta-analysis to comprehensively evaluate the effects of multiple TCM mind-body exercises on stroke survivors’ limb function and ADL. This approach effectively overcomes the methodological limitations of traditional meta-analyses in multiple-intervention comparison scenarios and generates efficacy hierarchies for each outcome measure based on SUCRA rankings (36). Furthermore, analyses were conducted to examine the optimal intervention duration, frequency, and age applicability. These findings provide an evidence-based foundation for rehabilitation professionals when tailoring programs to target specific functional deficits, thereby filling the evidence gap regarding the comparative efficacy of various TCM mind-body exercises in post-stroke limb function and ADL rehabilitation.

However, this study has the following limitations: (1) some included studies bear a high risk of bias due to the lack of rigorous blinding designs, which limits the overall quality of the evidence; therefore, the results should be interpreted with caution; (2) no restrictions were imposed on patients’ disease duration or intervention length, and a relatively small number of studies on certain individual techniques (e.g., WQX) were included.

Future studies should pay closer attention to the disease course of stroke patients, further optimize experimental designs, and advocate for large-sample, multicenter, rigorously designed randomized controlled trials to refine the efficacy profiles of different TCM mind-body exercises. Additionally, future research should stratify the duration and intensity of these interventions to explore their dose–response relationships.

## 5 Conclusion

This study fully substantiates the core value of multiple TCM mind-body exercises (TJ, BDJ, YJJ, and WQX) in improving limb function and ADLs among stroke patients. Furthermore, it provides optimal choices regarding intervention selection, duration, frequency, and age applicability when developing clinical rehabilitation programs. Given the economical and convenient nature of TCM mind-body exercises, alongside their profound historical heritage, future practice and promotion should focus on two main directions. First, from a disease prevention perspective, it is recommended to promote TCM mind-body exercises of appropriate intensity to healthy populations to effectively reduce the risk of cardiovascular and cerebrovascular diseases. Second, from a clinical rehabilitation perspective, these exercises should be actively integrated as standard complementary therapies for stroke survivors to further facilitate the restoration of impaired motor functions and comprehensively enhance their overall quality of life.

## Data Availability

The original contributions presented in the study are included in the article/Supplementary material, further inquiries can be directed to the corresponding author.

## References

[1] Purrahman D Shojaeian A Poniatowski ŁA Piechowski-Jóźwiak B Mahmoudian-Sani M-R. The role of progranulin (PGRN) in the pathogenesis of ischemic stroke. Cell Mol Neurobiol. (2023) 43:3435–47. doi: 10.1007/s10571-023-01396-8, 37561339 PMC11410000

[2] Feigin VL Stark BA Johnson CO Roth GA Bisignano C Abady GG . Global, regional, and national burden of stroke and its risk factors, 1990–2019: a systematic analysis for the global burden of disease study 2019. Lancet Neurol. (2021) 20:795–820. doi: 10.1016/S1474-4422(21)00252-0, 34487721 PMC8443449

[3] Oliveira CB Medeiros IRT Greters MG Frota NAF Lucato LT Scaff M . Abnormal sensory integration affects balance control in hemiparetic patients within the first year after stroke. Clinics. (2011) 66:2043–8. doi: 10.1590/S1807-59322011001200008, 22189728 PMC3226598

[4] Billinger SA Arena R Bernhardt J Eng JJ Franklin BA Johnson CM . Physical activity and exercise recommendations for stroke survivors a statement for healthcare professionals from the American heart association/american stroke association. Stroke. (2014) 45:2532–53. doi: 10.1161/STR.0000000000000022, 24846875

[5] Kyu HH Bachman VF Alexander LT Mumford JE Afshin A Estep K . Physical activity and risk of breast cancer, colon cancer, diabetes, ischemic heart disease, and ischemic stroke events: systematic review and dose-response meta-analysis for the global burden of disease study 2013. BMJ. (2016) 354:i3857. doi: 10.1136/bmj.i3857, 27510511 PMC4979358

[6] Gorna S Domaszewska K. The effect of endurance training on serum BDNF levels in the chronic post-stroke phase: current evidence and qualitative systematic review. J Clin Med. (2022) 11:3556. doi: 10.3390/jcm11123556, 35743624 PMC9225034

[7] Middleton LE Corbett D Brooks D Sage MD MacIntosh BJ McIlroy WE . Physical activity in the prevention of ischemic stroke and improvement of outcomes: a narrative review. Neurosci Biobehav Rev. (2013) 37:133–7. doi: 10.1016/j.neubiorev.2012.11.011 23201860

[8] Mascitelli L Pezzetta F. Anti-inflammatory effect of physical activity. Arch Intern Med. (2004) 164:460–13. doi: 10.1001/archinte.164.4.460-a 14981000

[9] Saunders DH Sanderson M Hayes S Johnson L Kramer S Carter DD . Physical fitness training for stroke patients. Cochrane Database Syst Rev. (2020) 3:CD003316. doi: 10.1002/14651858.CD003316.pub7, 32196635 PMC7083515

[10] Wang X-Q Li L Zou L Chen KW Liu J. Traditional Chinese exercise for chronic diseases. Evid Based Complement Altern Med. (2022) 2022:9842104. doi: 10.1155/2022/9842104, 35126607 PMC8813252

[11] García-Muñoz C González-García P Casuso-Holgado MJ Martínez-Calderón J Heredia-Rizo AM. Are movement-based mindful exercises (QIGONG, TAI CHI, AND YOGA) beneficial for stroke and parkinson’s disease? A scoping review. Complement Ther Med. (2023) 72:102912. doi: 10.1016/j.ctim.2022.102912 36565791

[12] Desai R Tailor A Bhatt T. Effects of yoga on brain waves and structural activation: a review. Complement Ther Clin Pract. (2015) 21:112–8. doi: 10.1016/j.ctcp.2015.02.002, 25824030

[13] Zhao J Zang Y Chau JPC He R Thompson DR. Chinese stroke survivors’ perceptions of participation in exercise or sitting tai chi. Eur J Cardiovasc Nurs. (2022) 21:143–51. doi: 10.1093/eurjcn/zvab036, 34008005

[14] Fogaça LZ Portella CFS Ghelman R Abdala CVM Schveitzer MC. Mind-body therapies from traditional Chinese medicine: evidence map. Front Public Health. (2021) 9:659075. doi: 10.3389/fpubh.2021.659075, 34988045 PMC8722380

[15] Winser SJ Tsang WWN Krishnamurthy K Kannan P. Does tai chi improve balance and reduce falls incidence in neurological disorders? A systematic review and meta-analysis. Clin Rehabil. (2018) 32:1157–68. doi: 10.1177/0269215518773442, 29737198

[16] Zheng G Zheng X Li J Duan T Tao J Chen L. Effect of tai chi on cardiac and static pulmonary function in older community-dwelling adults at risk of ischemic stroke: a randomized controlled trial. Chin J Integr Med. (2019) 25:582–9. doi: 10.1007/s11655-018-3056-5, 30519872

[17] Feng F Luo X-C Chen Y-J Li J-J Kang H Yan B-H. Effects of tai chi yunshou on upper-limb function and balance in stroke survivors: a systematic review and meta-analysis. Complement Ther Clin Pract. (2023) 51:101741. doi: 10.1016/j.ctcp.2023.101741 36868000

[18] Ye M Zheng Y Xiong Z Ye B Zheng G. Baduanjin exercise ameliorates motor function in patients with post-stroke cognitive impairment: a randomized controlled trial. Complement Ther Clin Pract. (2022) 46:101506. doi: 10.1016/j.ctcp.2021.101506, 34742096

[19] Tian J Gao Y Zhang J Yang Z Dong S Zhang T . Progress and challenges of network meta-analysis. J Evid Based Med. (2021) 14:218–31. doi: 10.1111/jebm.12443, 34463038

[20] Hutton B Salanti G Caldwell DM Chaimani A Schmid CH Cameron C . The PRISMA extension statement for reporting of systematic reviews incorporating network meta-analyses of health care interventions: checklist and explanations. Ann Intern Med. (2015) 162:777–84. doi: 10.7326/M14-2385 26030634

[21] Xu C Niu Y Wu J Gu H Zhang C. Software and package applicating for network meta-analysis: a usage-based comparative study. J Evid Based Med. (2018) 11:176–83. doi: 10.1111/jebm.12264, 29266878

[22] Yin Y Wang Z Huang L Zhao Y Guan Q Xu H . Orthodontic maximum anchorages in malocclusion treatment: a systematic review and network meta-analysis. J Evid Based Med. (2021) 14:295–302. doi: 10.1111/jebm.12453, 34904788

[23] Liu T. (1994). A discussion on the definition of medical qigong. J Beijing Univ Chin Med. Available online at: https://kns.cnki.net/KCMS/detail/detail.aspx?dbcode=CJFQ&dbname=CJFD9495&filename=JZYB405.007 (accessed April 28, 2025).

[24] Li GY Wang W Liu GL Zhang Y. Effects of tai chi on balance and gait in stroke survivors: a systematic meta-analysis of randomized controlled trials. J Rehabil Med. (2018) 50:582–8. doi: 10.2340/16501977-2346, 29736553

[25] Niu J-F Zhao X-F Hu H-T Wang J-J Liu Y-L Lu D-H. Should acupuncture, biofeedback, massage, qi gong, relaxation therapy, device-guided breathing, yoga and tai chi be used to reduce blood pressure?: recommendations based on high-quality systematic reviews. Complement Ther Med. (2019) 42:322–31. doi: 10.1016/j.ctim.2018.10.017 30670261

[26] Xu J Zhang Z Liu J Li Y Yu L Wan J . Effect of traditional asian exercise on patients with chronic heart failure: a protocol for network meta-analysis of randomised controlled trials. BMJ Open. (2021) 11:e048891. doi: 10.1136/bmjopen-2021-048891, 34452962 PMC8404441

[27] Li RY Chen K-Y Wang X-R Yu Q Xu L. Comparison of different rehabilitation techniques of traditional Chinese and western medicine in the treatment of motor dysfunction after stroke based on frequency method a network meta-analysis. Am J Phys Med Rehabil. (2023) 102:504–12. doi: 10.1097/PHM.0000000000002130, 36731006 PMC10184820

[28] Xie B Yang M Bai Y (2019) Clinical study on the effect of Baduanjin on motor rehabilitation of stroke patients. West China Med 34: 515–519. Available online at: https://kns.cnki.net/KCMS/detail/detail.aspx?dbcode=CJFQ&dbname=CJFDLAST2019&filename=HXYX201905009 (accessed April 28, 2025).

[29] Zou L Wang C Chen X Wang H. Baduanjin exercise for stroke rehabilitation: a systematic review with meta-analysis of randomized controlled trials. Int J Environ Res Public Health. (2018) 15:600. doi: 10.3390/ijerph15040600, 29584623 PMC5923642

[30] Yuen M Ouyang HX Miller T Pang MYC. Baduanjin qigong improves balance, leg strength, and mobility in individuals with chronic stroke: a randomized controlled study. Neurorehabil Neural Repair. (2021) 35:444–56. doi: 10.1177/15459683211005020, 33825587

[31] Ashcroft SK Ironside DD Johnson L Kuys SS Thompson-Butel AG. Effect of exercise on brain-derived neurotrophic factor in stroke survivors: a systematic review and meta-analysis. Stroke. (2022) 53:3706–16. doi: 10.1161/STROKEAHA.122.039919, 36278401

[32] Che P Bao Y Ji Y Chen L Wu M Chen Y (2024) Precise assessment and analysis of the effects of the modified Wuqinxi on balance function in stroke patients. Zhongguo Kangfu Yixue Zazhi 39, 828–834. Available online at: https://kns.cnki.net/KCMS/detail/detail.aspx?dbcode=CJFQ&dbname=CJFDLAST2024&filename=ZGKF202406022 (accessed April 28, 2025).

[33] Yu Y Wu T Wu M Liu S Chen X Wu J . Evidence map of traditional Chinese exercises. Front Public Health. (2024) 12:1347201. doi: 10.3389/fpubh.2024.1347201, 39360254 PMC11445016

[34] Zhou Y Ren H Hou X Dong X Zhang S Lv Y . The effect of exercise on balance function in stroke patients: a systematic review and meta-analysis of randomized controlled trials. J Neurol. (2024) 271:4751–68. doi: 10.1007/s00415-024-12467-1, 38834700

[35] Luo B Zhang L Bai Y Zhang F Liu Y. Therapeutic effect of traditional Chinese exercise in stroke patients: an umbrella review of systematic reviews and meta-analyses of clinical trials. Eur J Integr Med. (2025) 74:102438. doi: 10.1016/j.eujim.2025.102438

[36] Cooksey K Veettil SK Chaiyakunapruk N Dhippayom T. The potential benefits of the TIP framework to characterize health services interventions for network meta-analysis. J Evid Based Med. (2023) 16:123–5. doi: 10.1111/jebm.12536, 37272326

