# Supplementary material for: Revealing mind-body interaction mechanisms: a network meta-analysis of the effects of traditional Chinese medicine mind-body exercise interventions on stroke rehabilitation
Source: Front Neurol. 2026 Mar 13;17:1759127. doi: 10.3389/fneur.2026.1759127 (PMC13021452; doi:10.3389/fneur.2026.1759127)
Supplement: Supplementary file 1 [file Table_1.docx]

# *Supplementary Material*

# Abbreviations

Activities Of Daily Living:ADL

Traditional Chinese Medicine:TCM

Tai Ji:TJ

Ba Duan Jin:BDJ

Yi Jin Jing:YJJ

Wu Qin Xi:WQX

Conventional Treatment:CT

# 1 Supplementary Figures and Tables

# 1.1 Supplementary Figures

# Supplementary Figure 1. Formulas for calculating baseline-to-endpoint change scores when standard deviations (SD) are unreported

$$\mathrm{SD}_{\mathrm{change}}=\sqrt{\mathrm{SD}_{\mathrm{baseline}}^{2}+\mathrm{SD}_{\mathrm{final}}^{2}-(2r\times\mathrm{SD}_{\mathrm{baseline}}\times\mathrm{SD}_{\mathrm{final}})}$$

# Supplementary Figure 2. Risk of Bias Graph


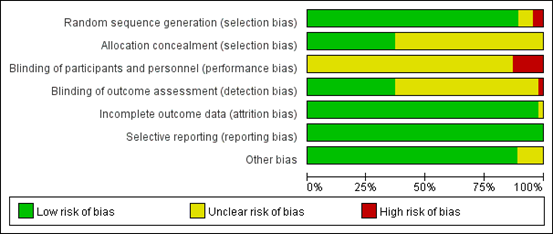


# Supplementary Figure 3. Summary table of risk of bias assessment


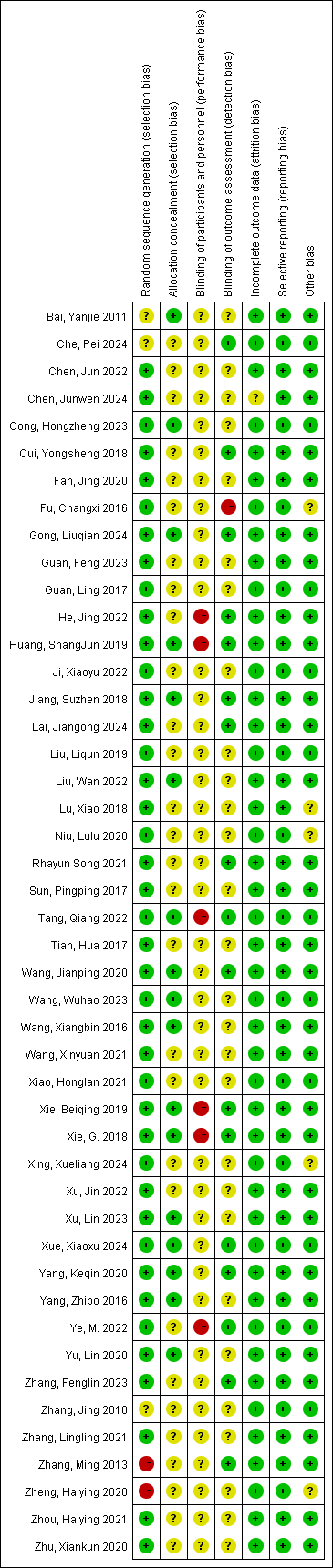


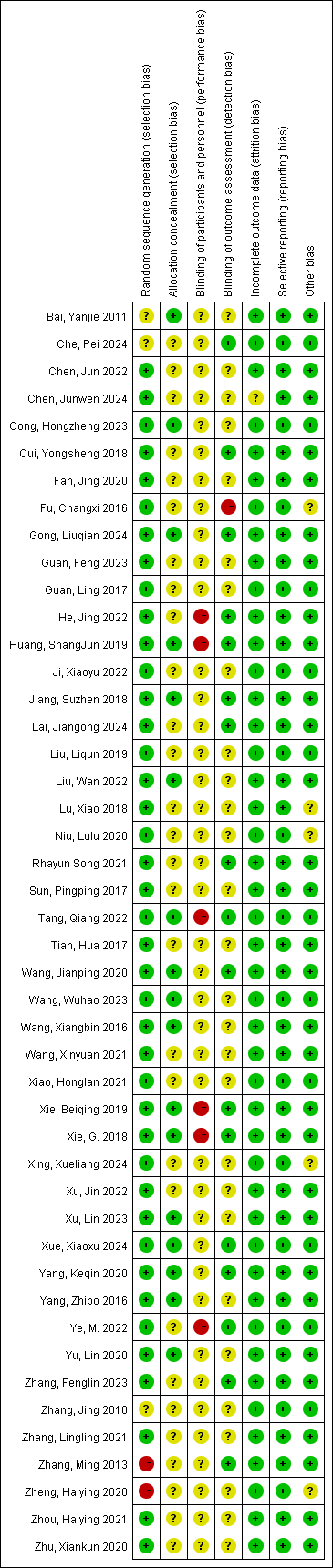


# Supplementary Figure 4. Forest plots of upper extremity function and two-by-two comparative forest plots

**A**

**
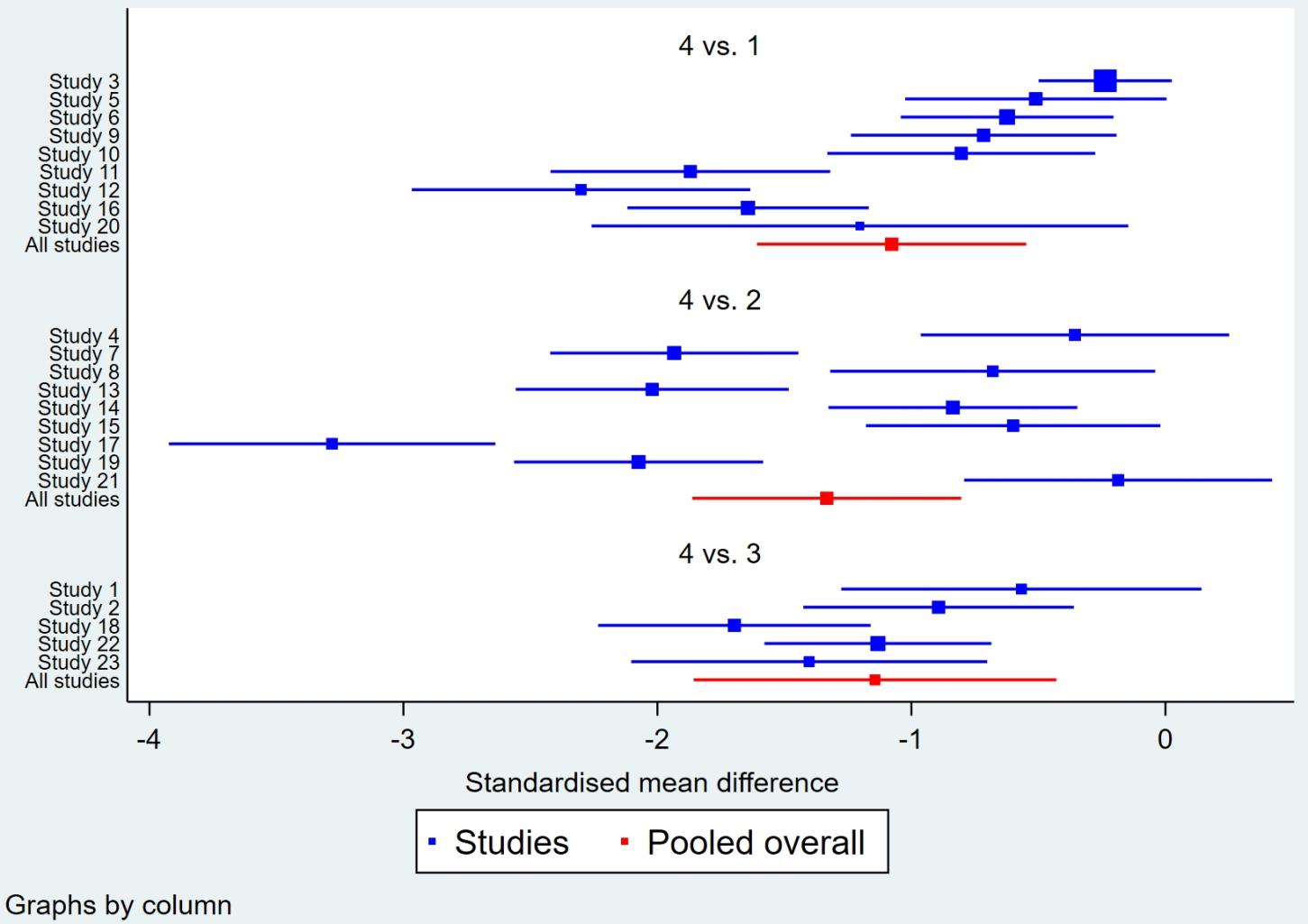
**

**B**

**
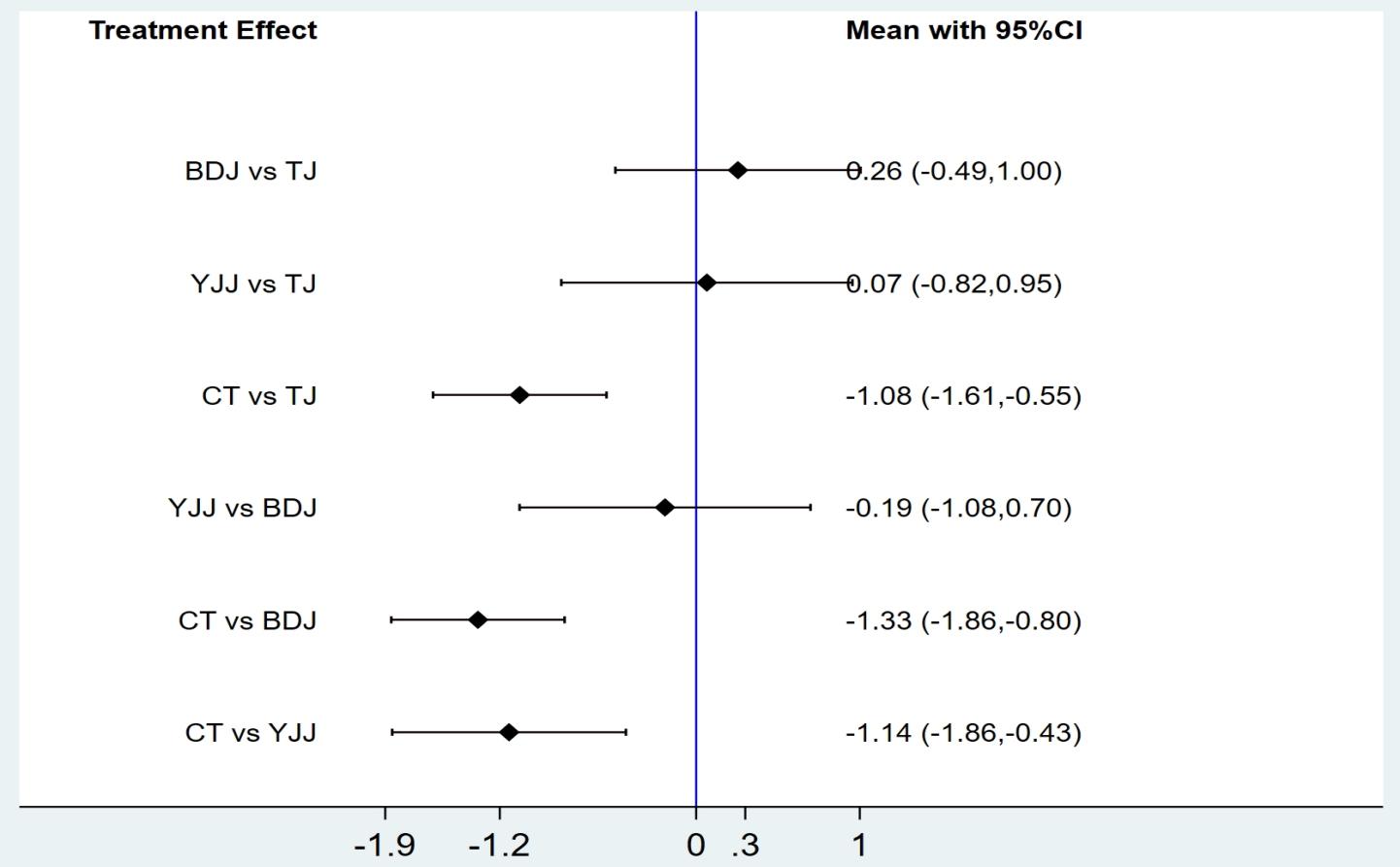
**

Notes:A:forest map；B:Two-by-two comparisons of forest maps.

# Supplementary Figure 5. Forest plots of lower extremity function and two-by-two comparative forest plots

**A**

**
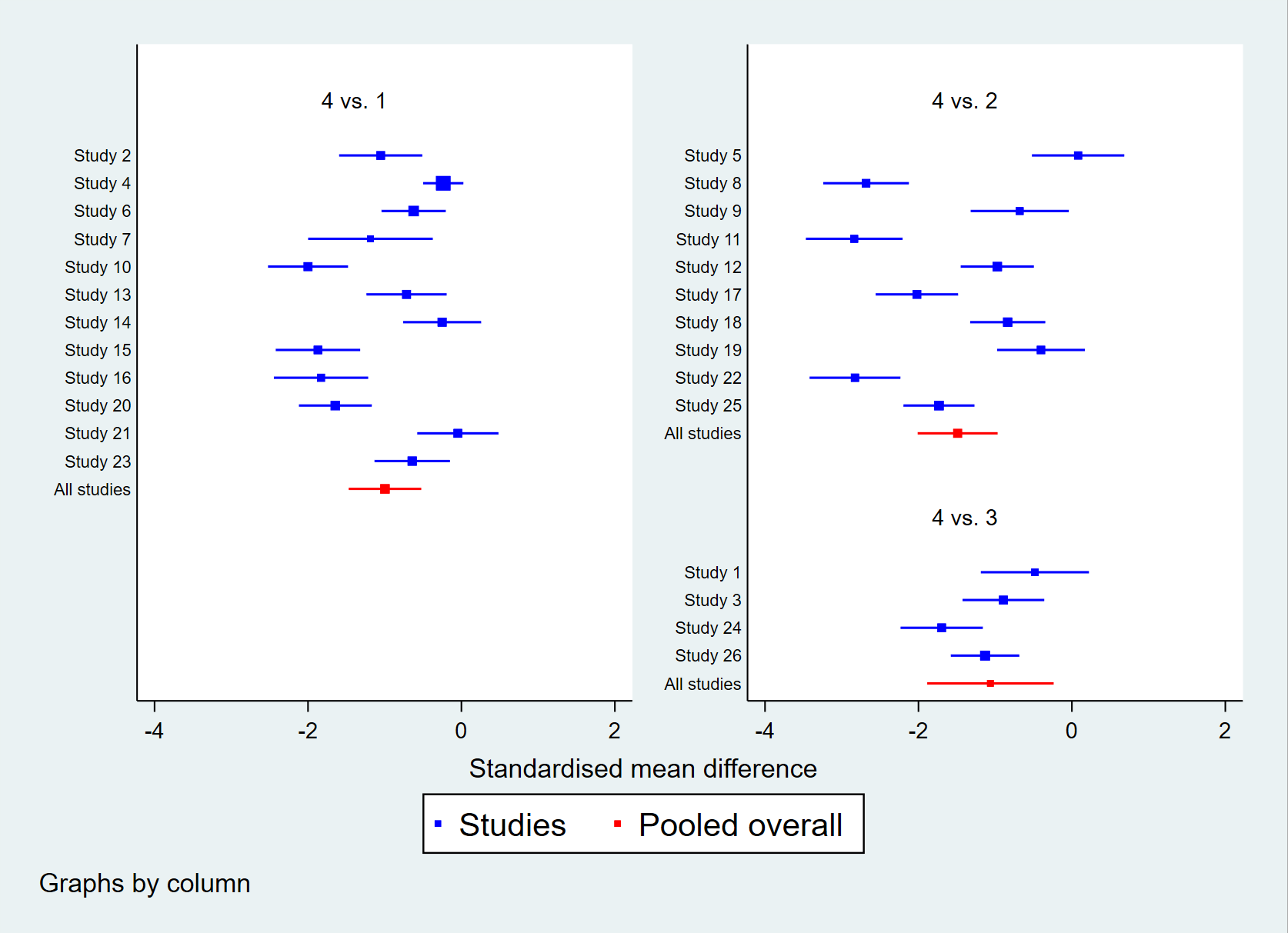
**

**B**

**
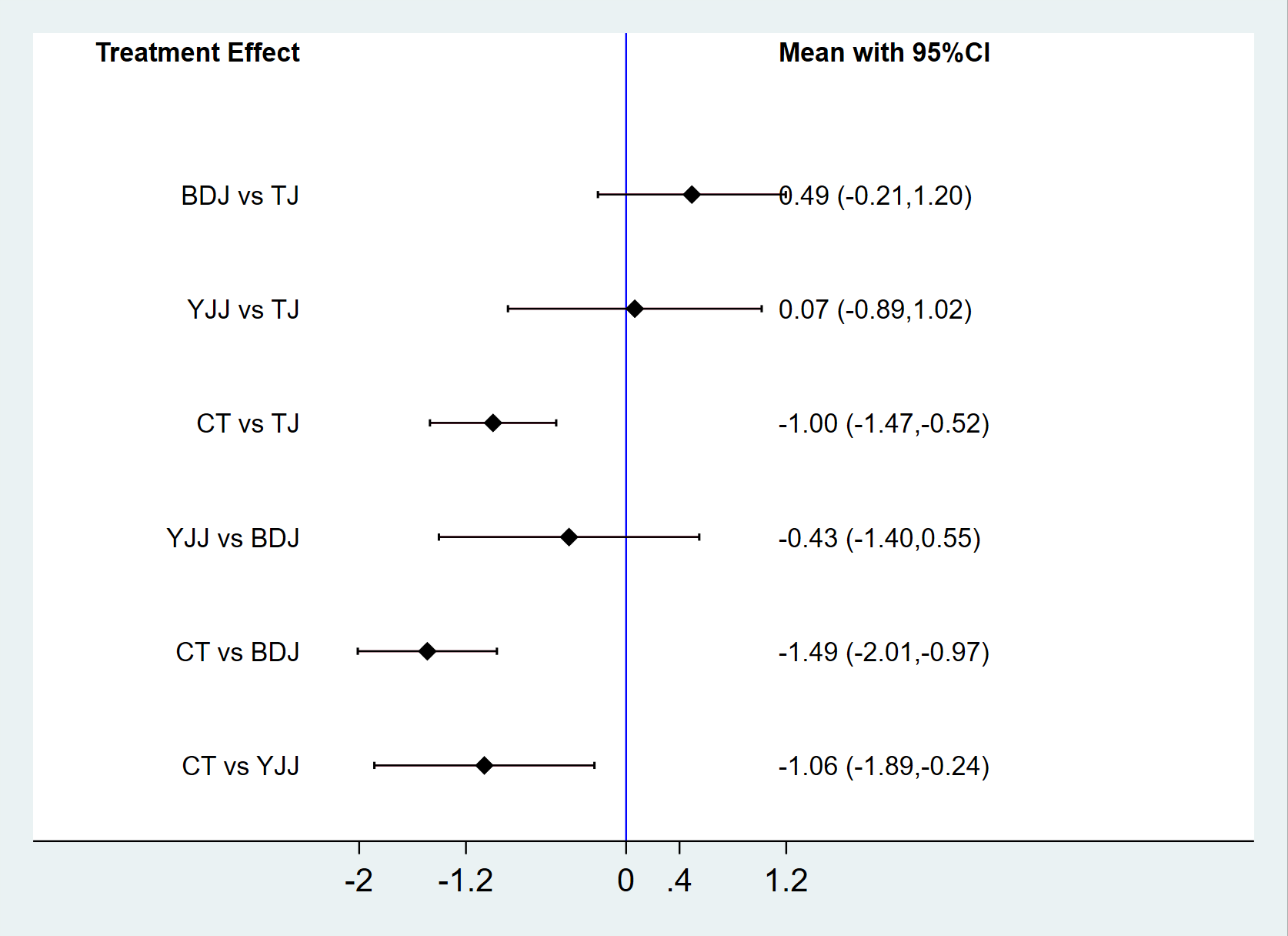
**

Notes:A:forest map；B:Two-by-two comparisons of forest maps.

# Supplementary Figure 6. Forest plots of balanced functions and two-by-two comparative forest plots

**A**

**
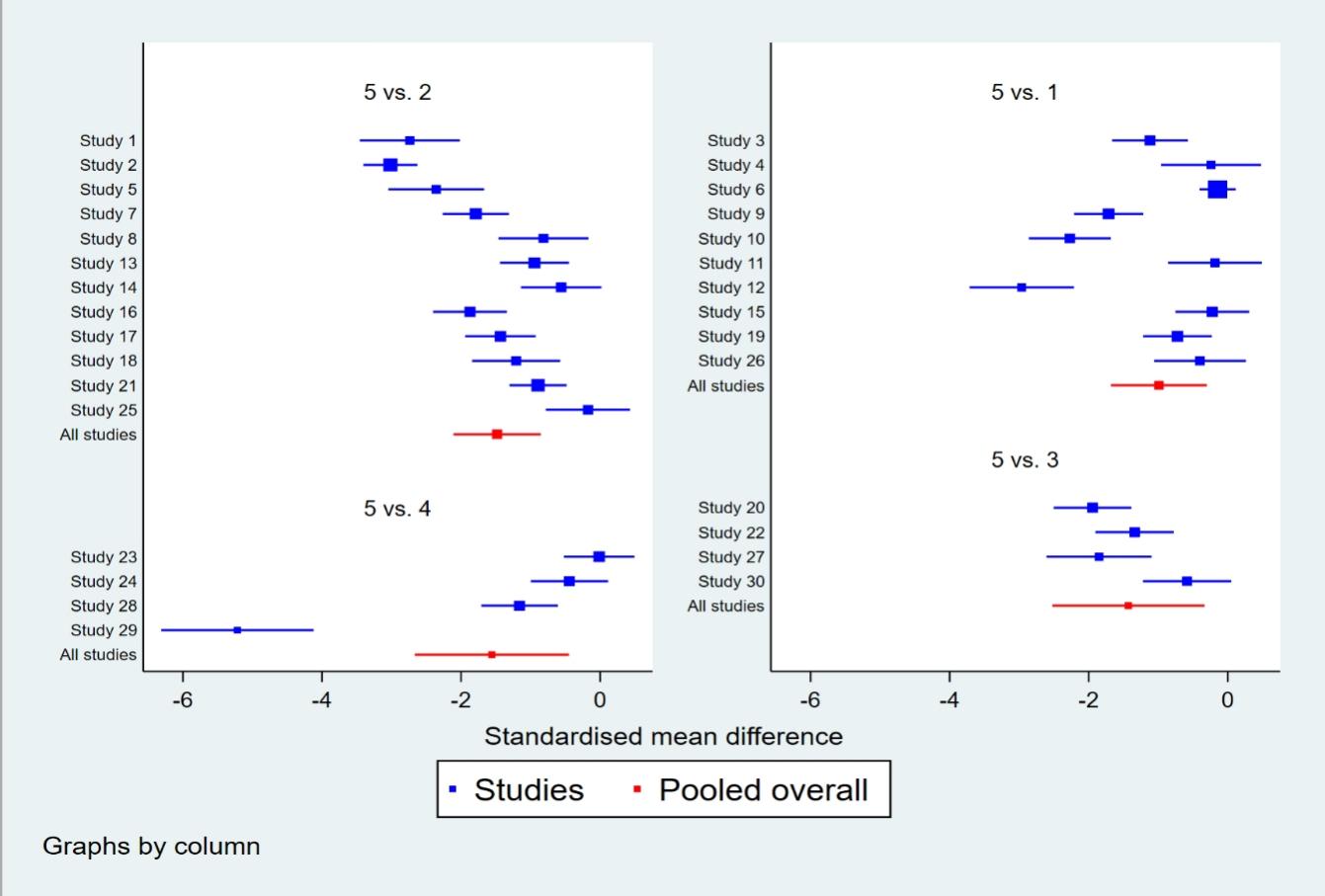
**

**B**

**
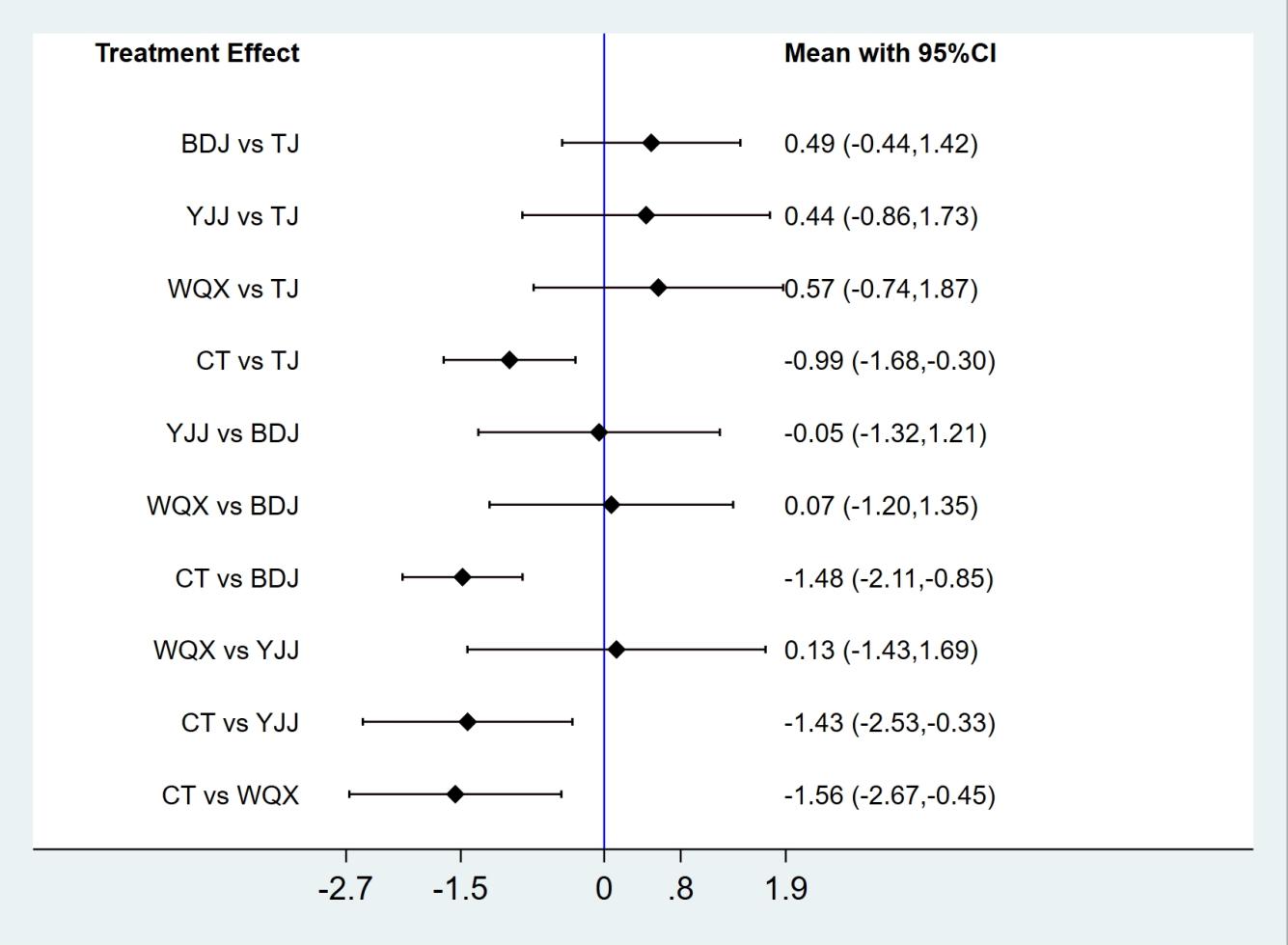
**

Notes:A:forest map；B:Two-by-two comparisons of forest maps.

# Supplementary Figure 7. Forest plots for ADL and two-by-two comparisons of forest plots

**A**

**
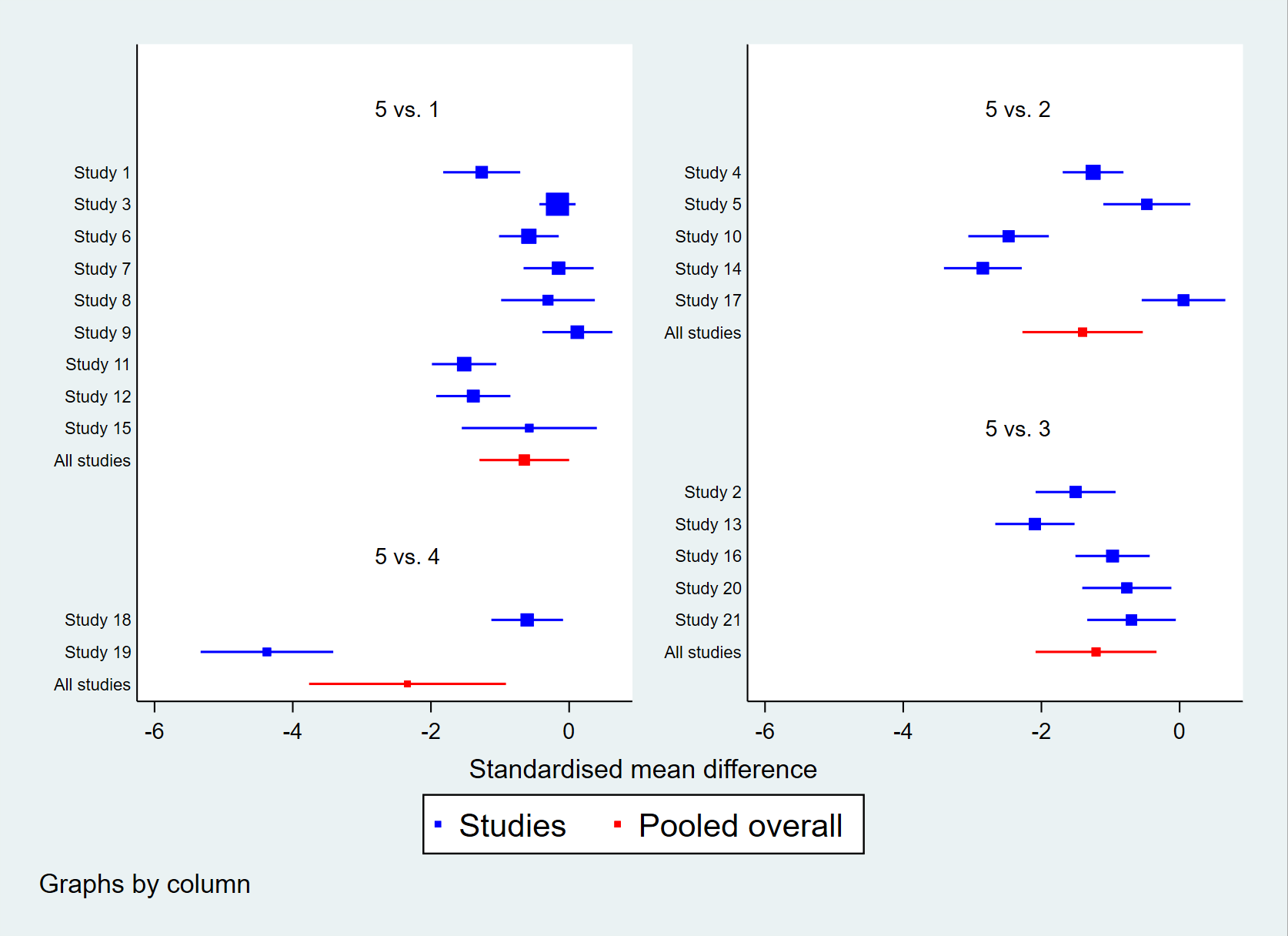
**

**B**

**
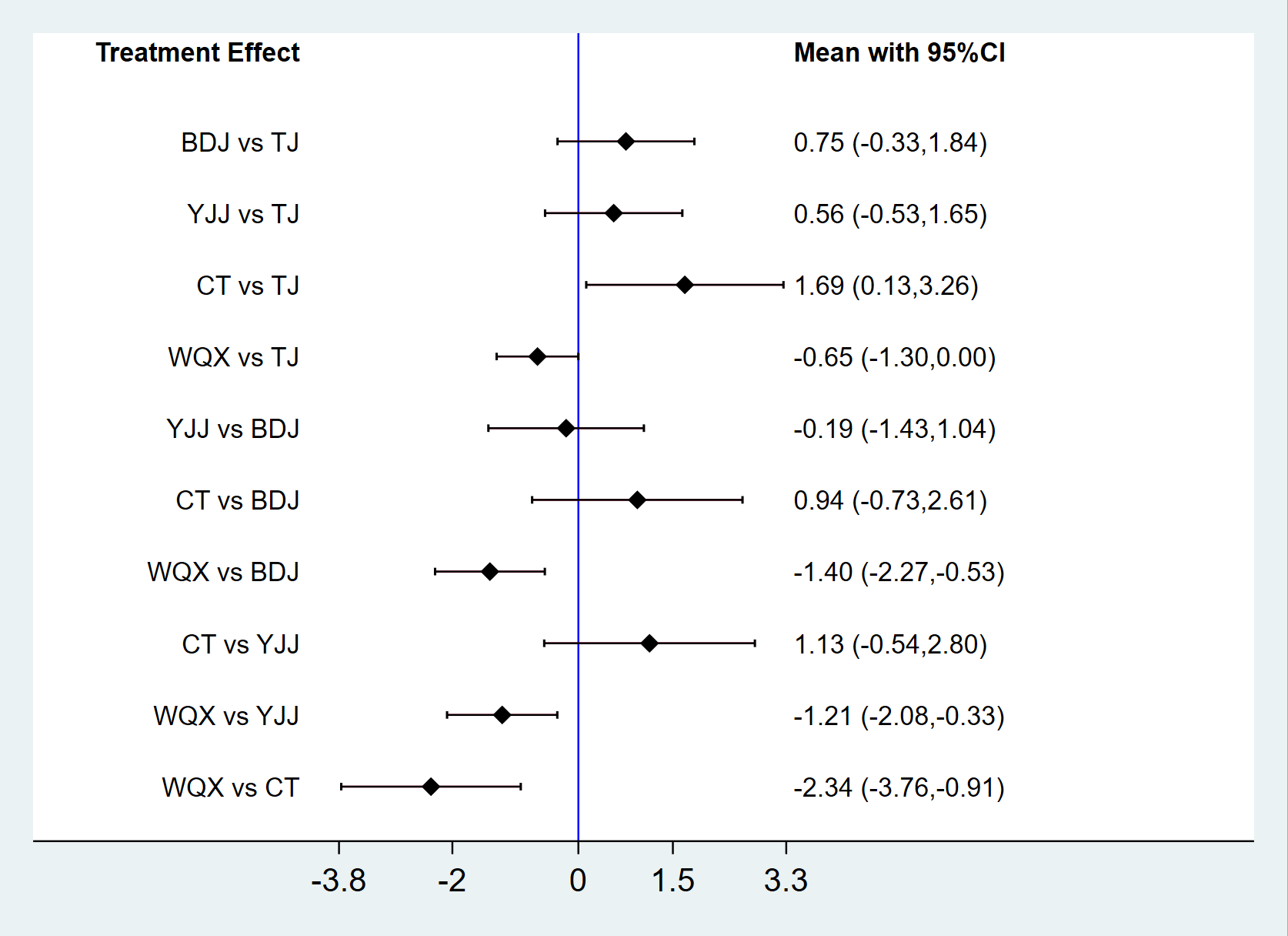
**

Notes:A:forest map；B:Two-by-two comparisons of forest maps.

# Supplementary Figure 8. Forest plot of subgroup analysis of upper limb function stratified by intervention cycle


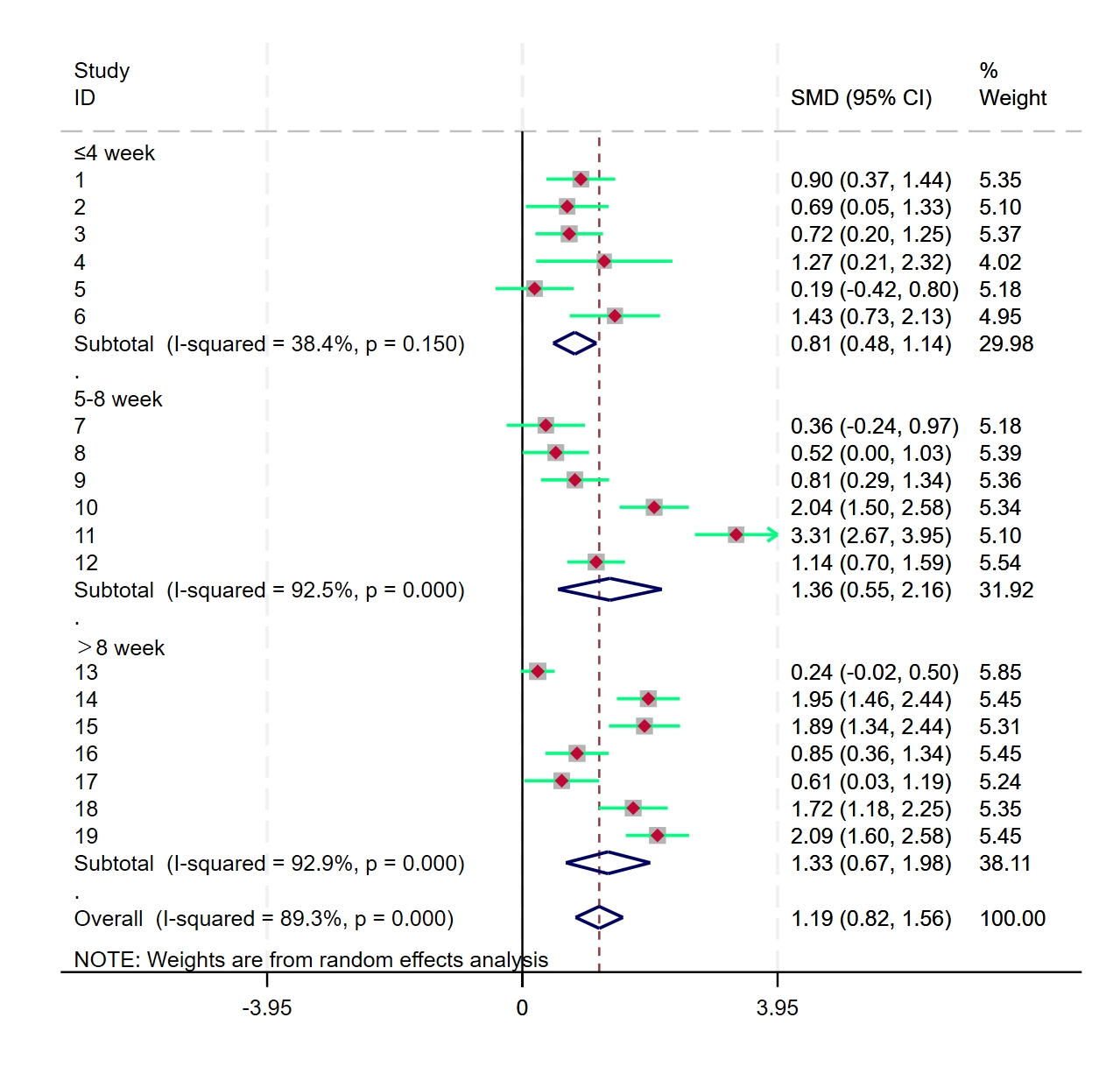


# Supplementary Figure 9. Forest plot of subgroup analysis of lower limb function stratified by intervention cycle

**
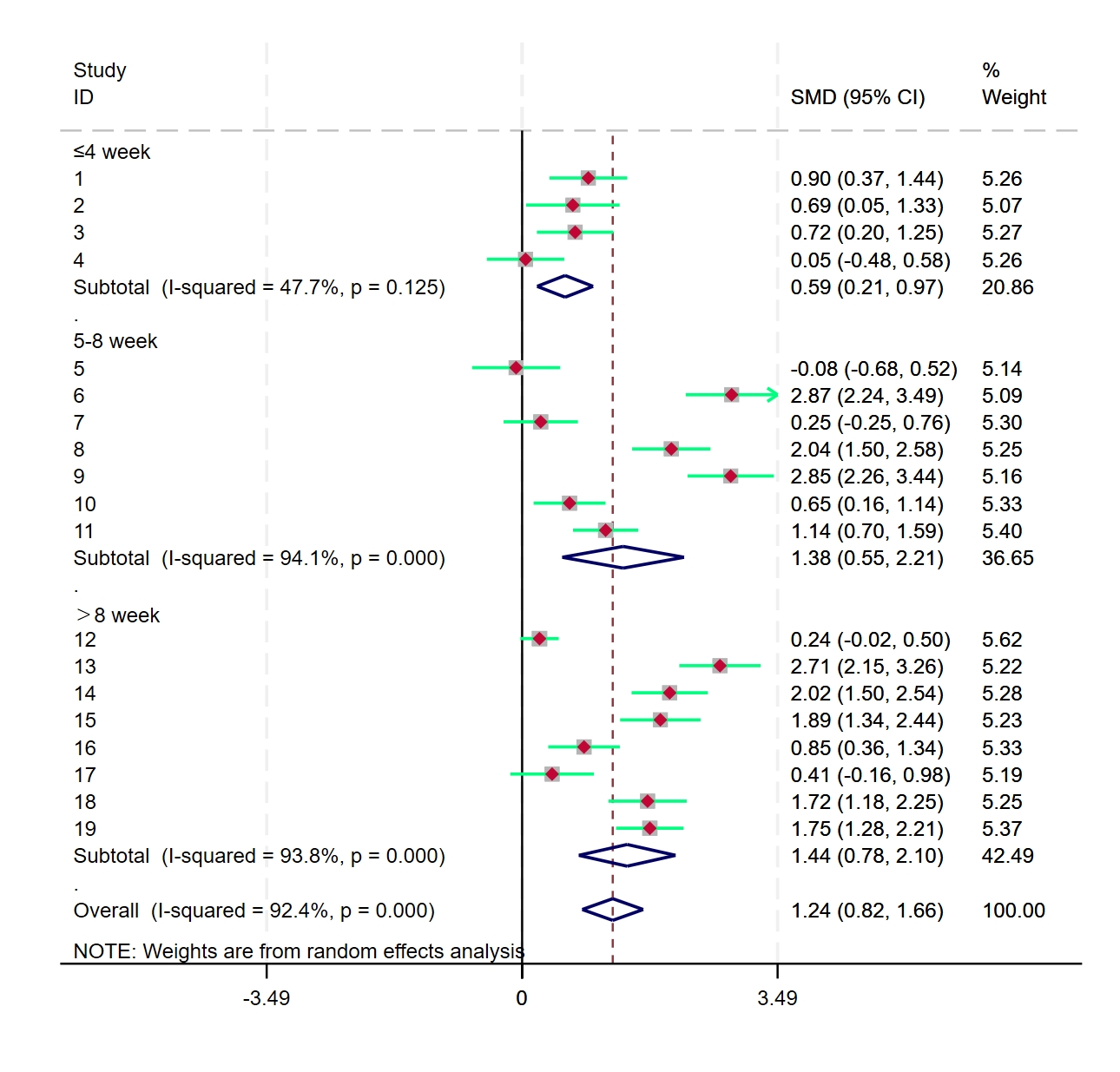
**

# Supplementary Figure 10. Forest plot of subgroup analysis of balance function stratified by intervention cycle

**
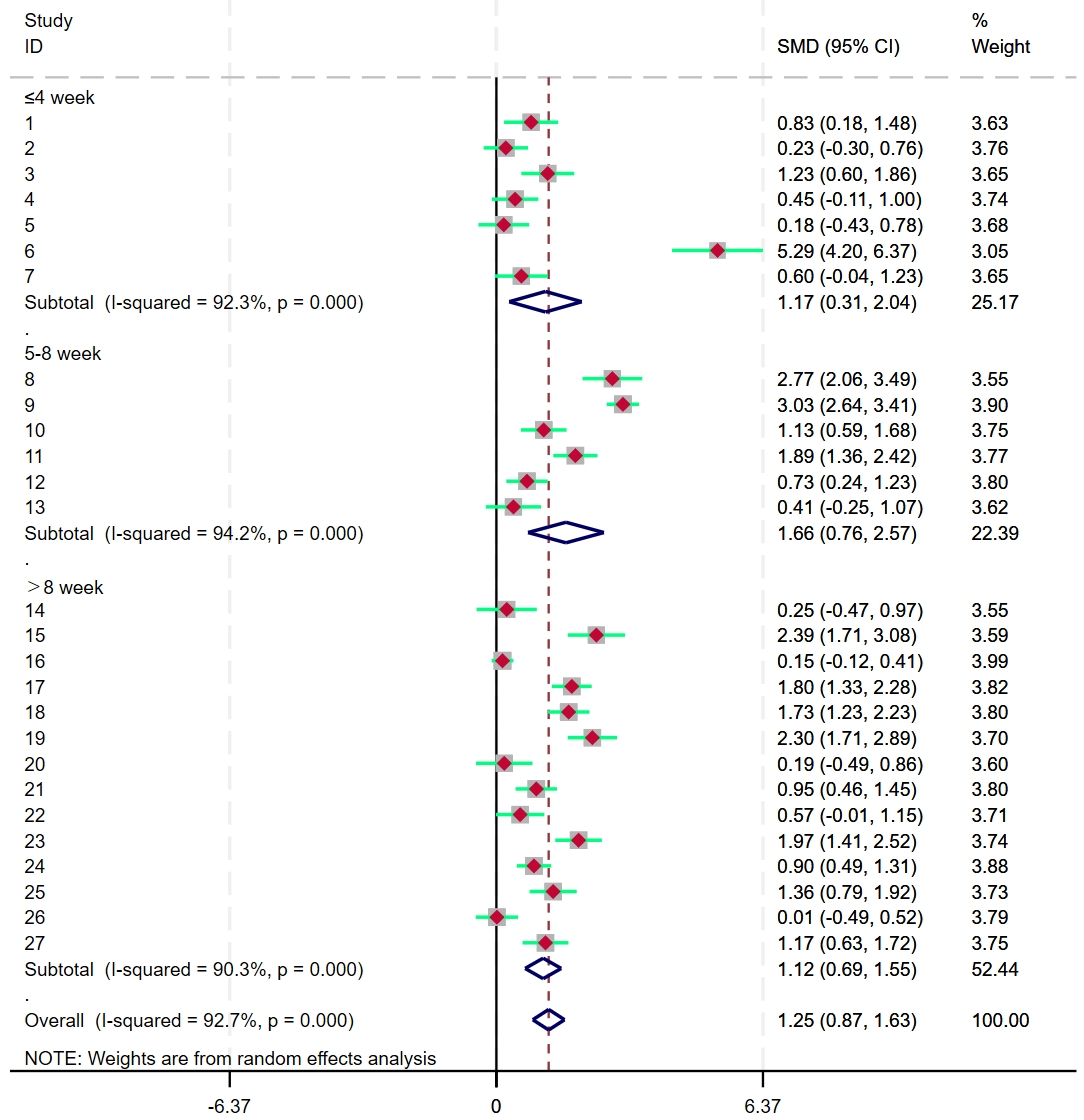
**

# Supplementary Figure 11. Forest plot of subgroup analysis of ADL stratified by intervention cycle

**
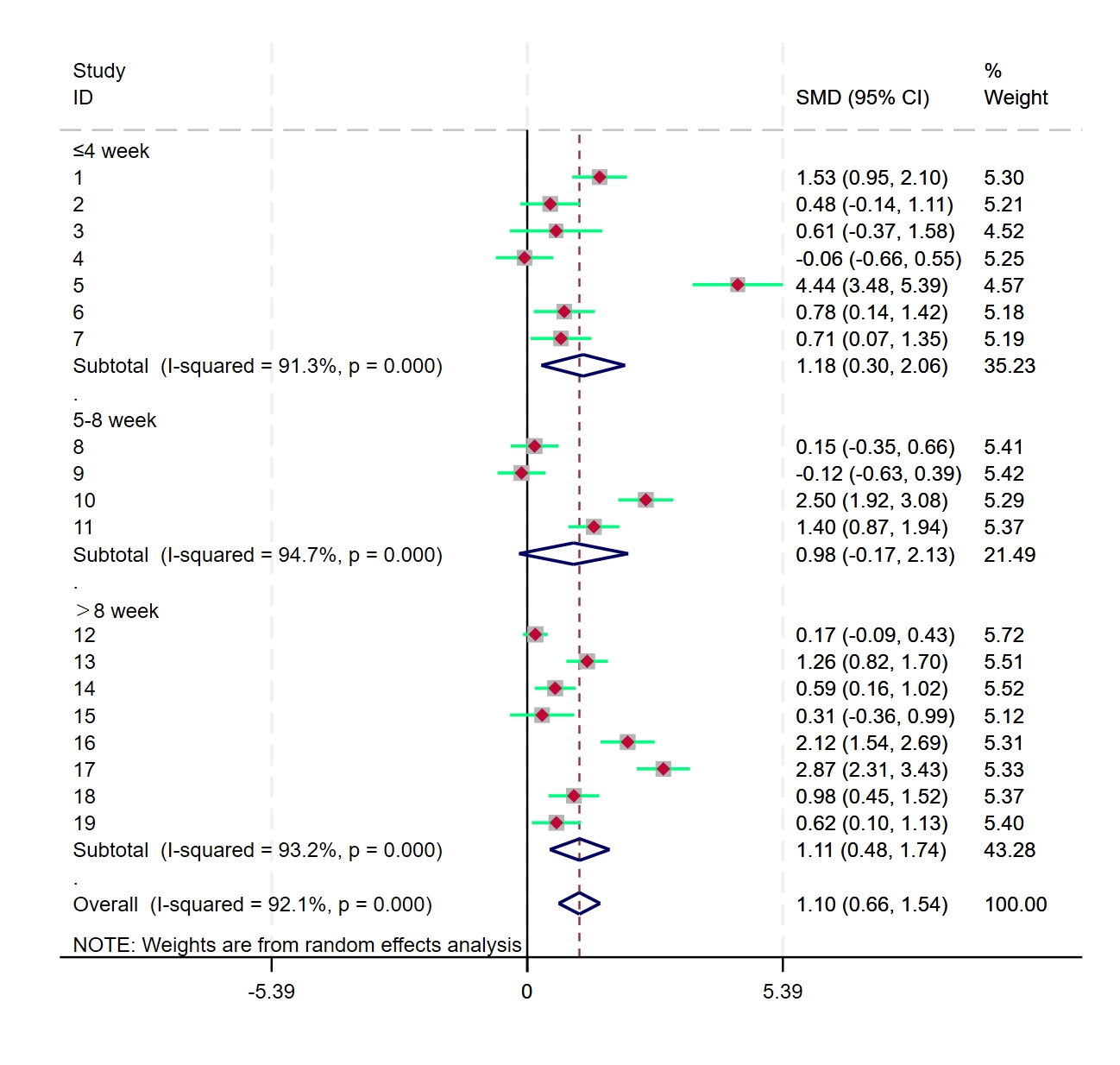
**

# Supplementary Figure 12. Forest plot of subgroup analysis of upper limb function stratified by exercise frequency


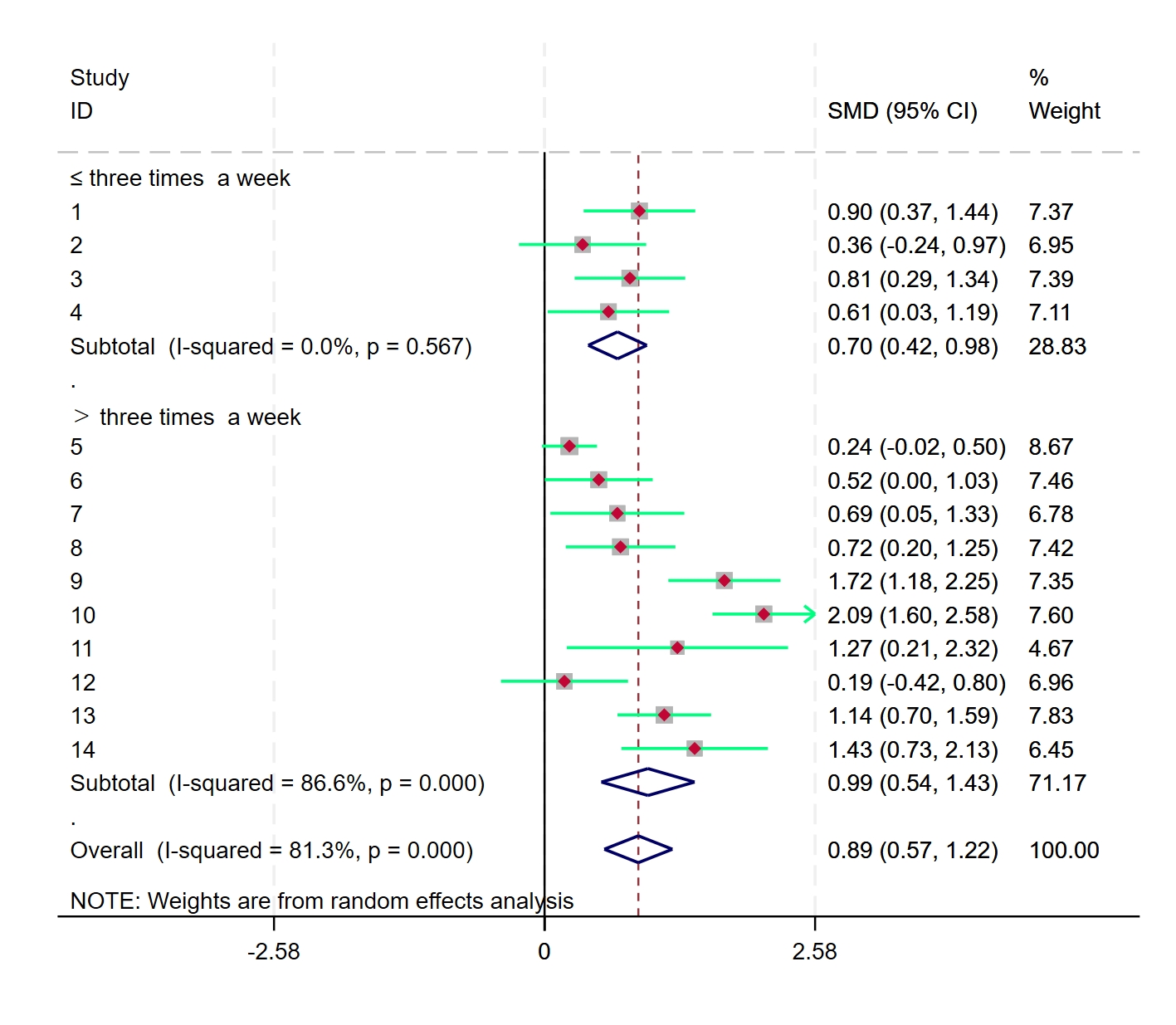


# Supplementary Figure 13. Forest plot of subgroup analysis of lower limb function stratified by exercise frequency


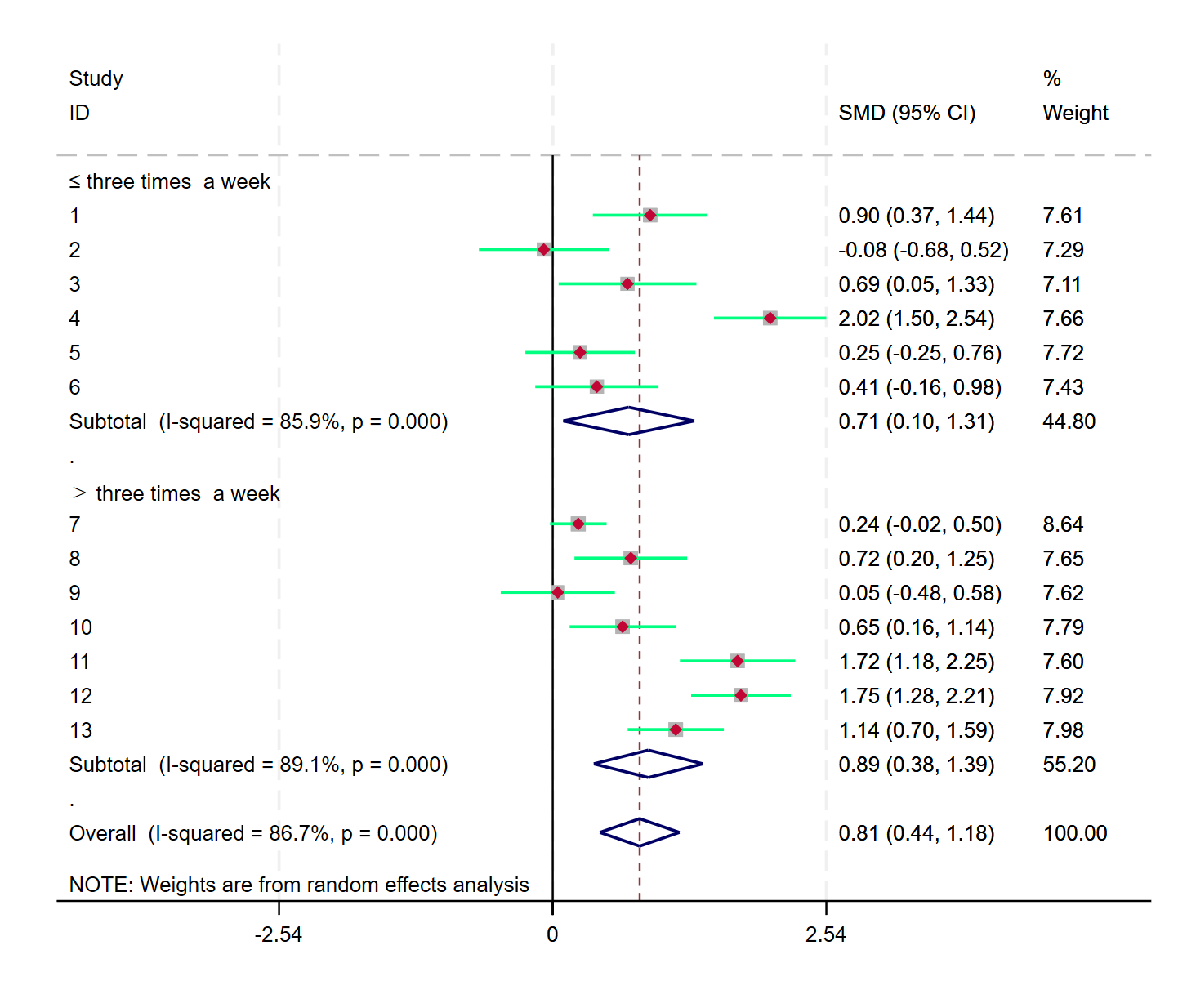


# Supplementary Figure 14. Forest plot of subgroup analysis of balance function stratified by exercise frequency


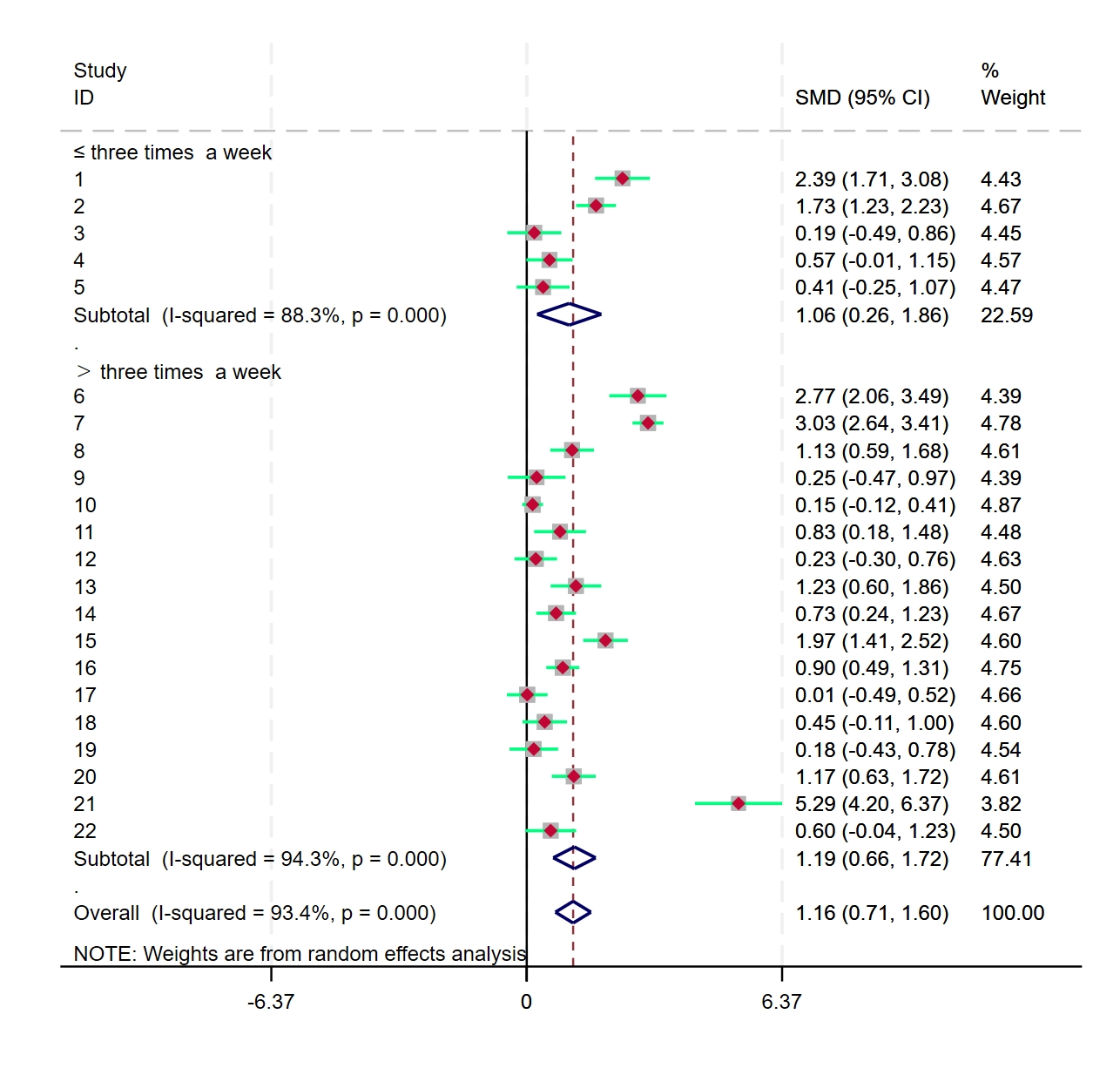


# Supplementary Figure 15. Forest plot of subgroup analysis of ADL stratified by exercise frequency


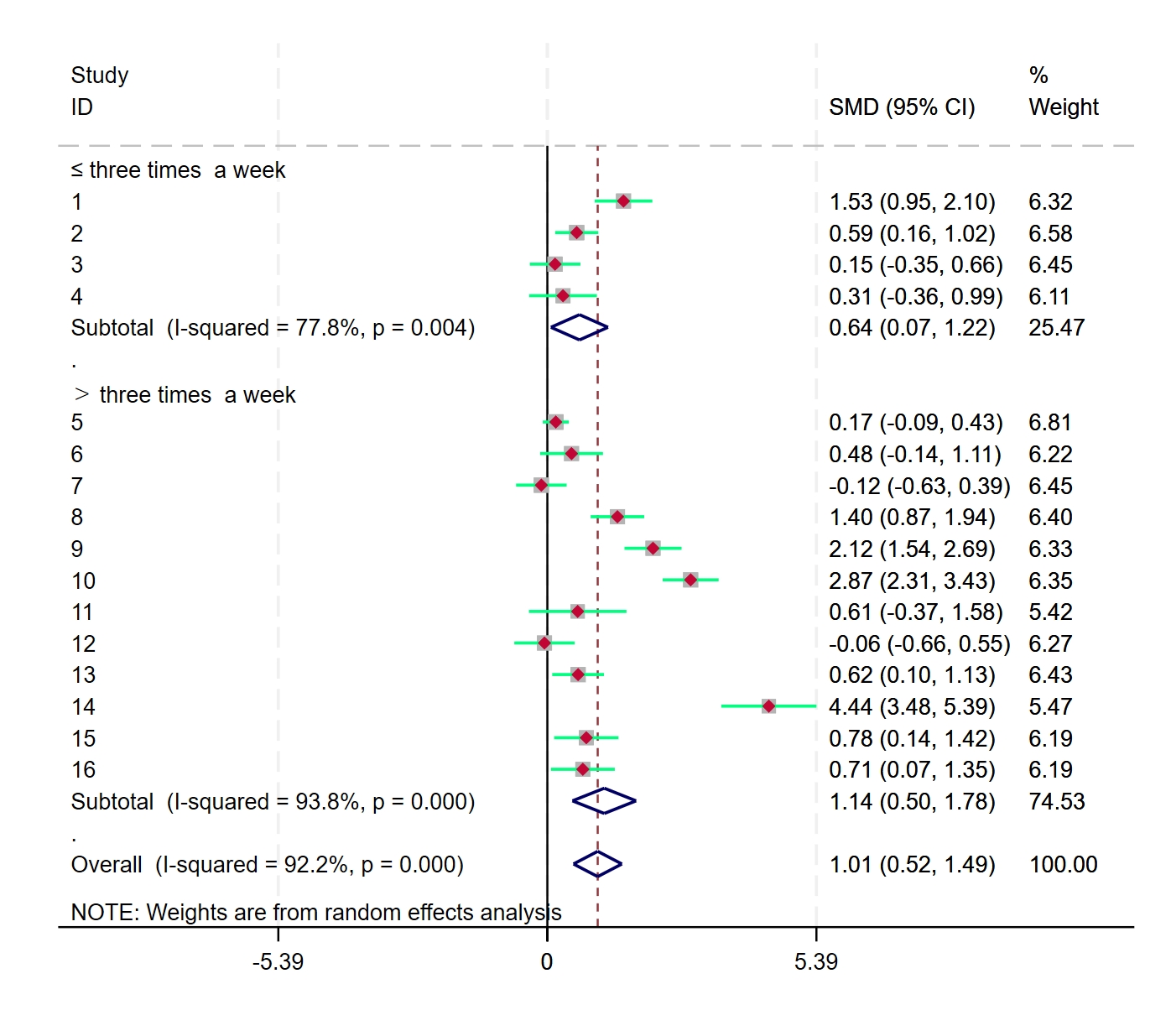


# Supplementary Figure 16. Forest plot of subgroup analysis of upper limb function stratified by patient age


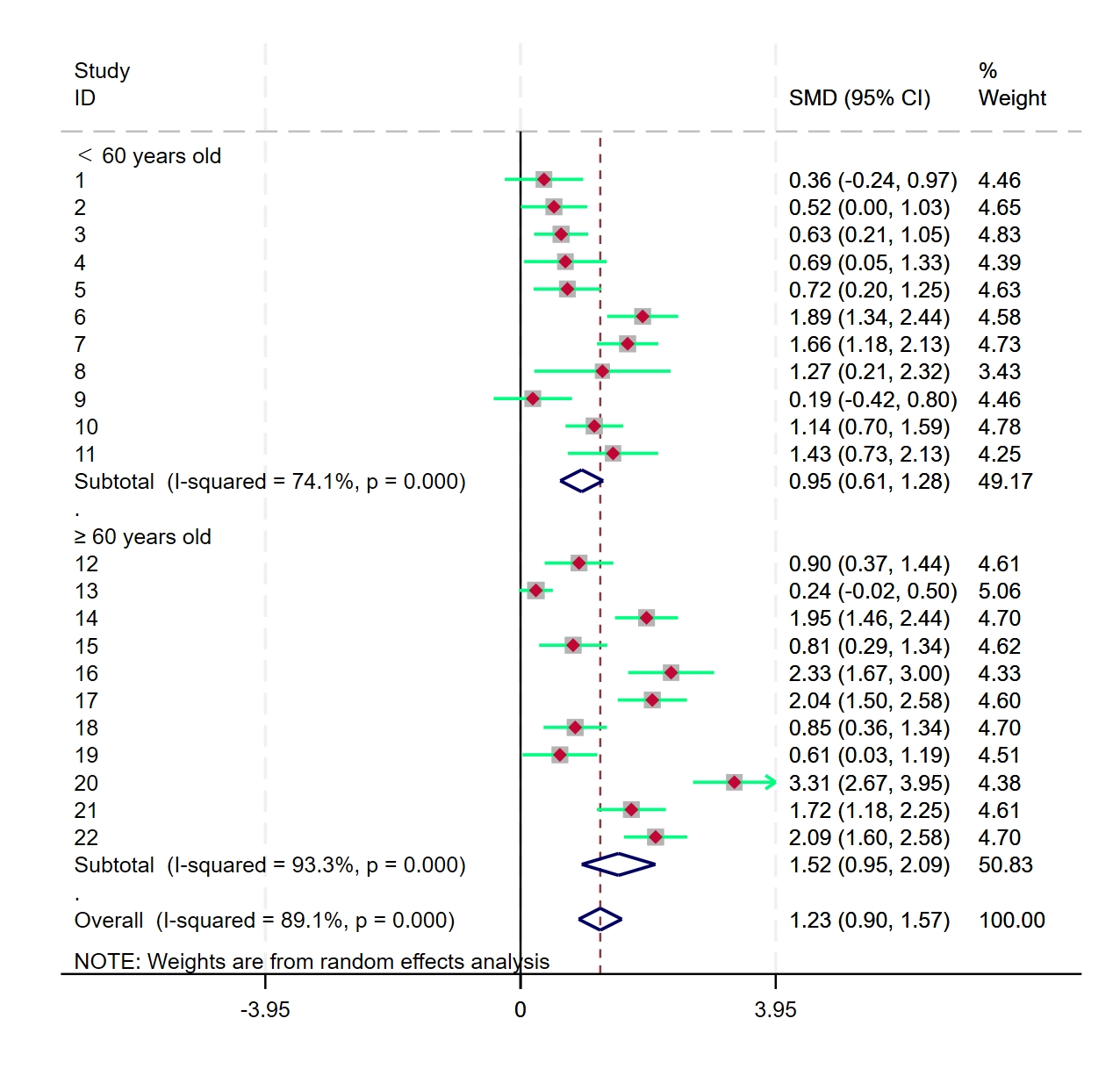


# Supplementary Figure 17. Forest plot of subgroup analysis of lower limb function stratified by patient age


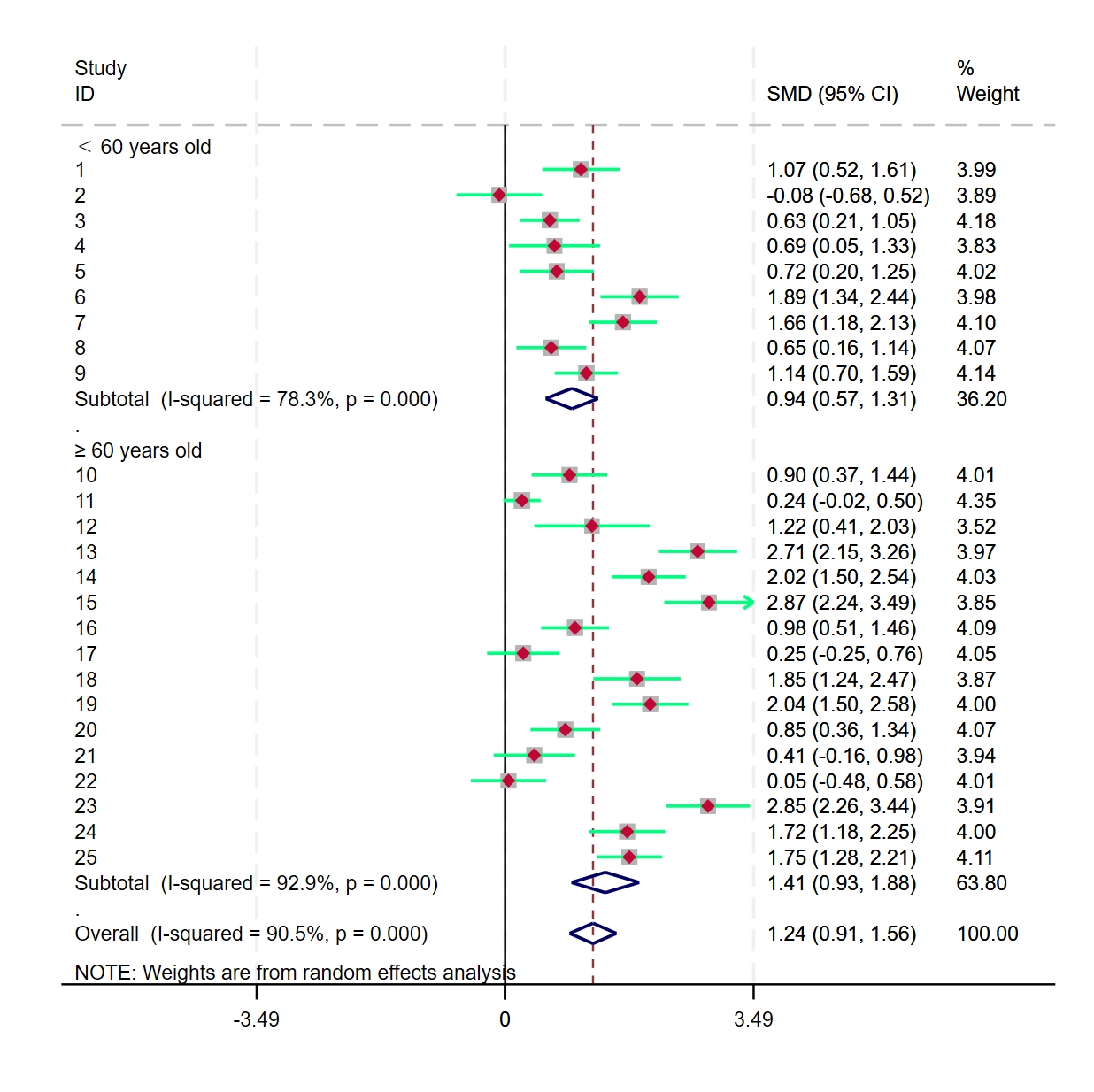


# Supplementary Figure 18. Forest plot of subgroup analysis of balance function stratified by patient age


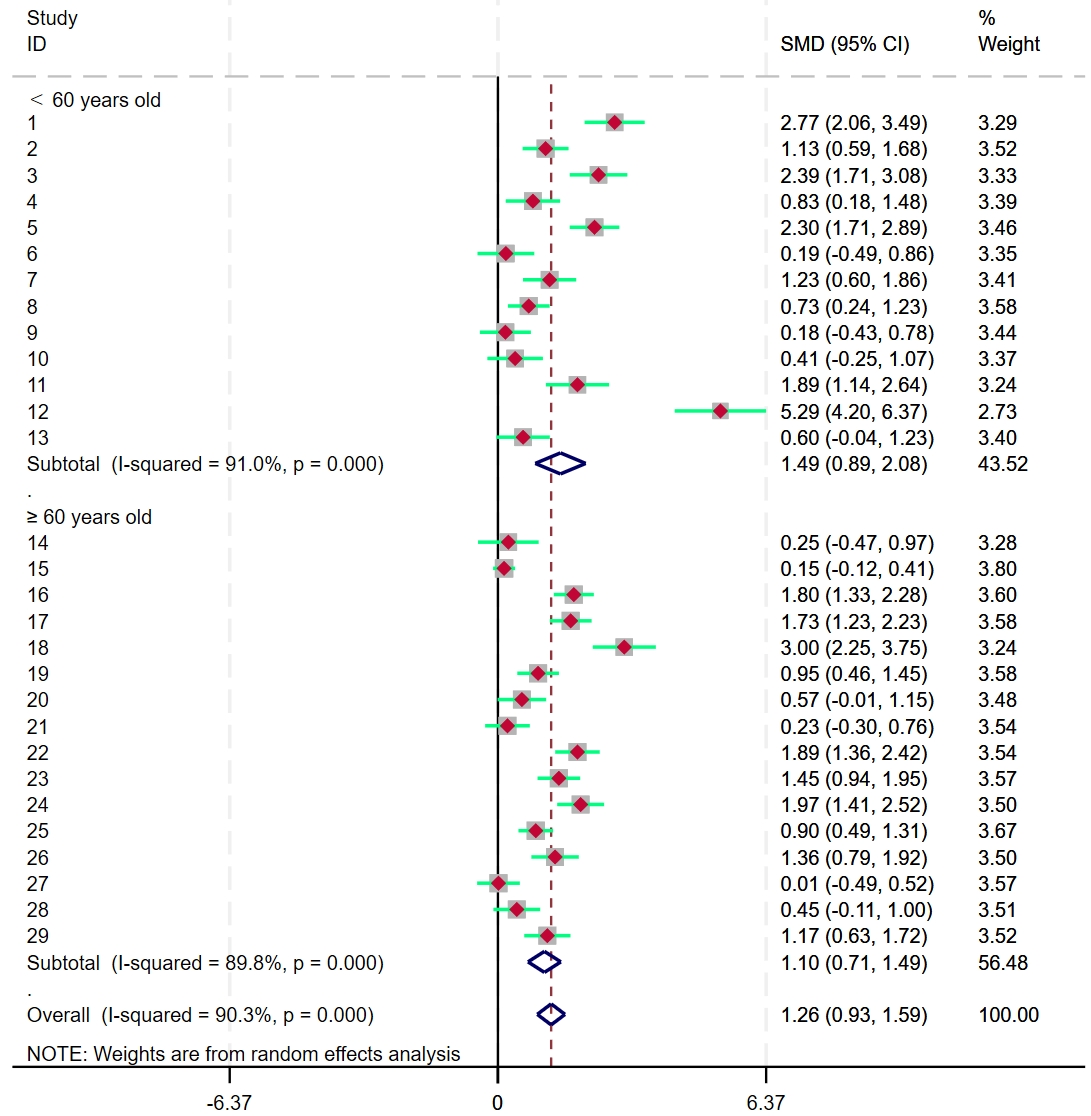


# Supplementary Figure 19. Forest plot of subgroup analysis of ADL stratified by patient age


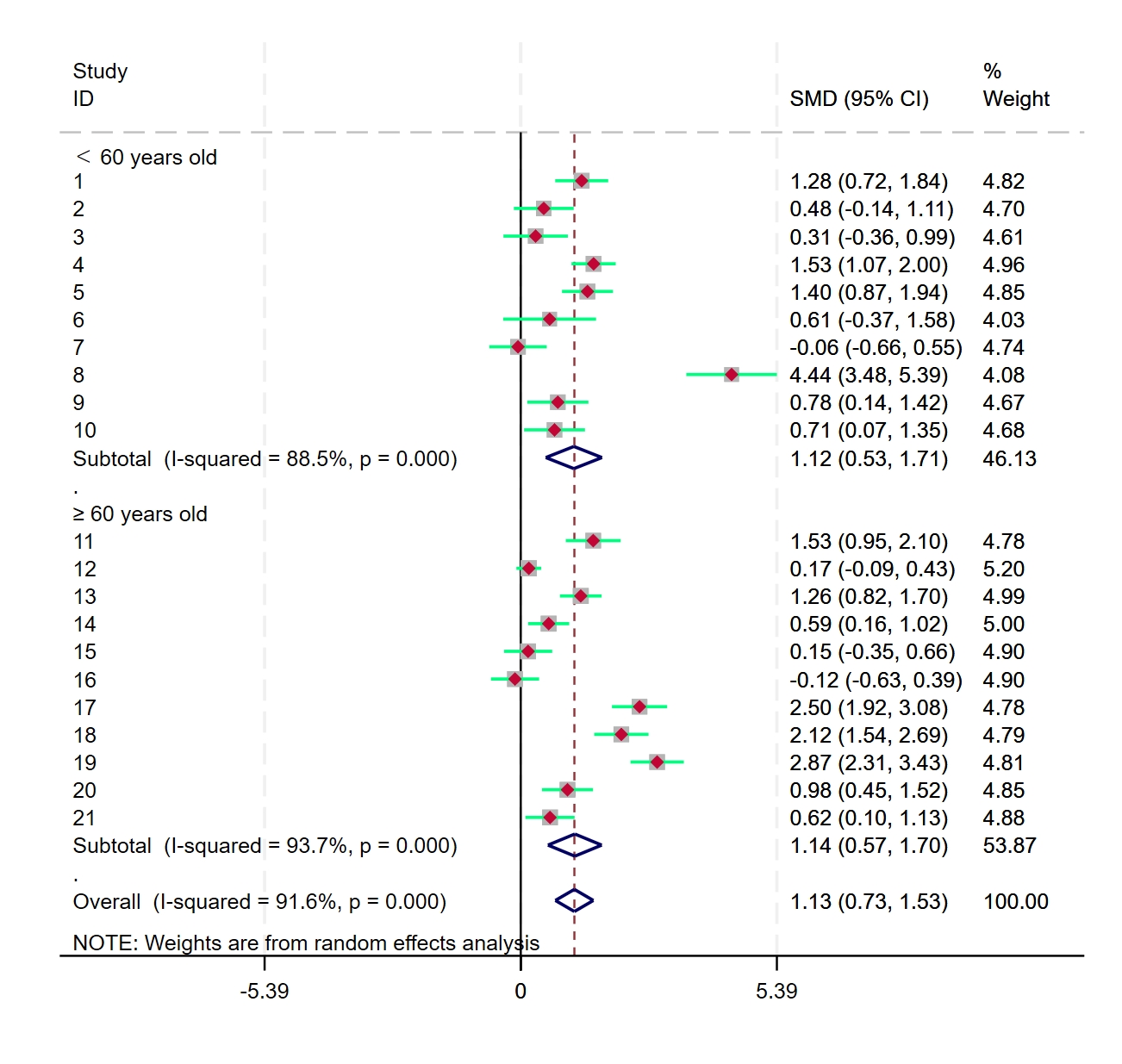


# Supplementary Figure 20. SUCRA table of different Chinese traditional exercise feats


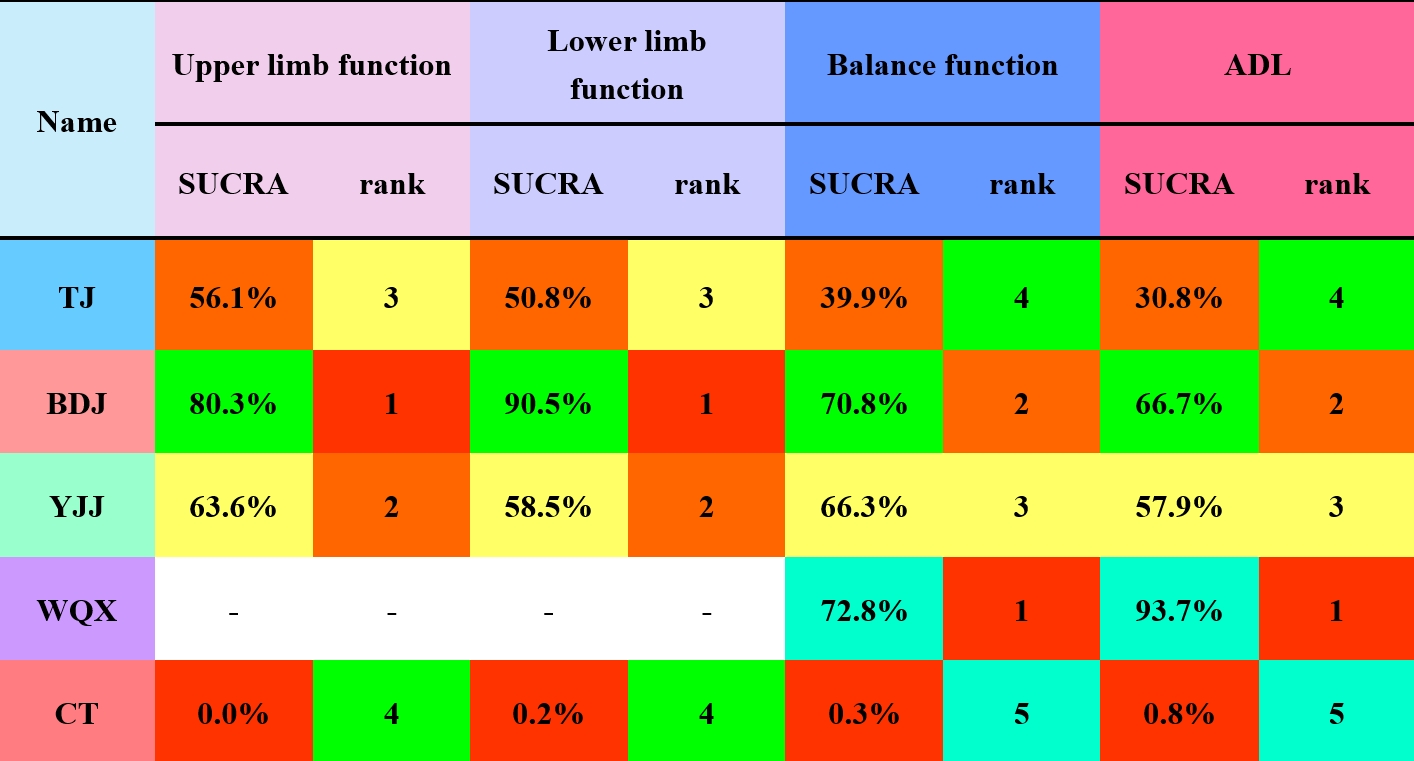


# Supplementary Figure 21. When upper limb function was used as the outcome indicator, robustness was tested using the one-by-one elimination method


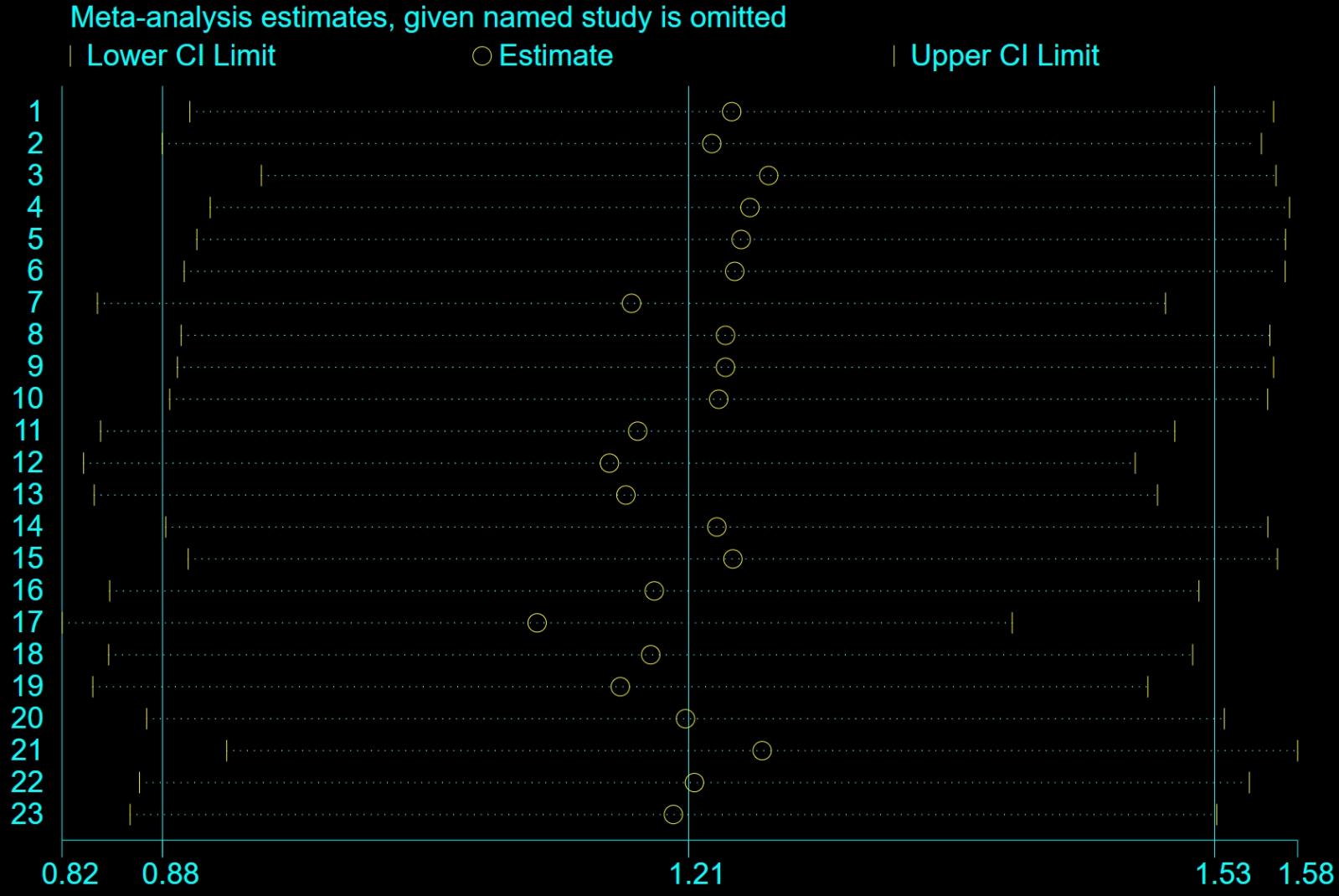


# Supplementary Figure 22. When lower limb function was used as the outcome indicator, robustness was tested using the one-by-one elimination method


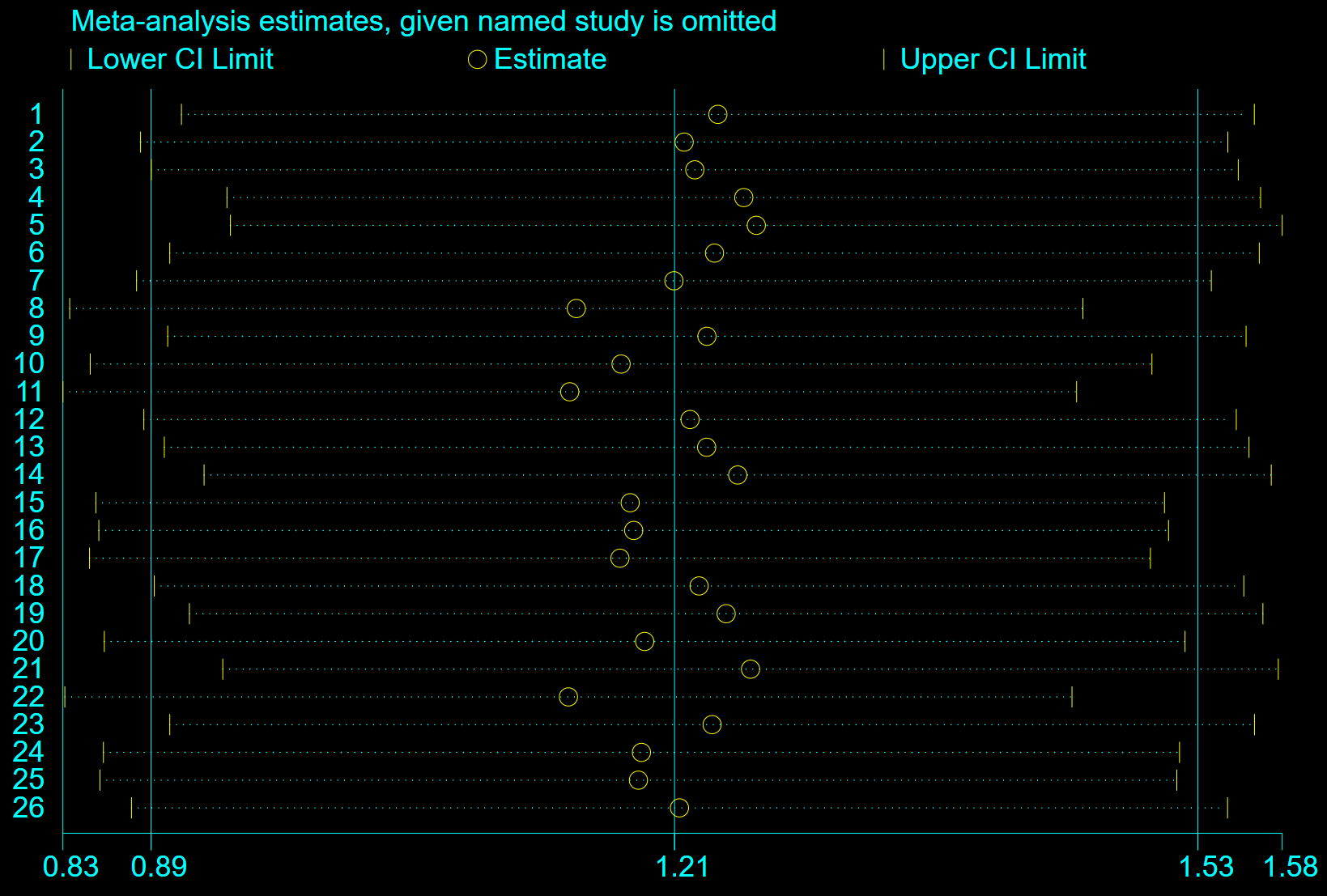


# Supplementary Figure 23. When the balance function is used as the ending indicator, the one-by-one elimination method is used to test for robustness


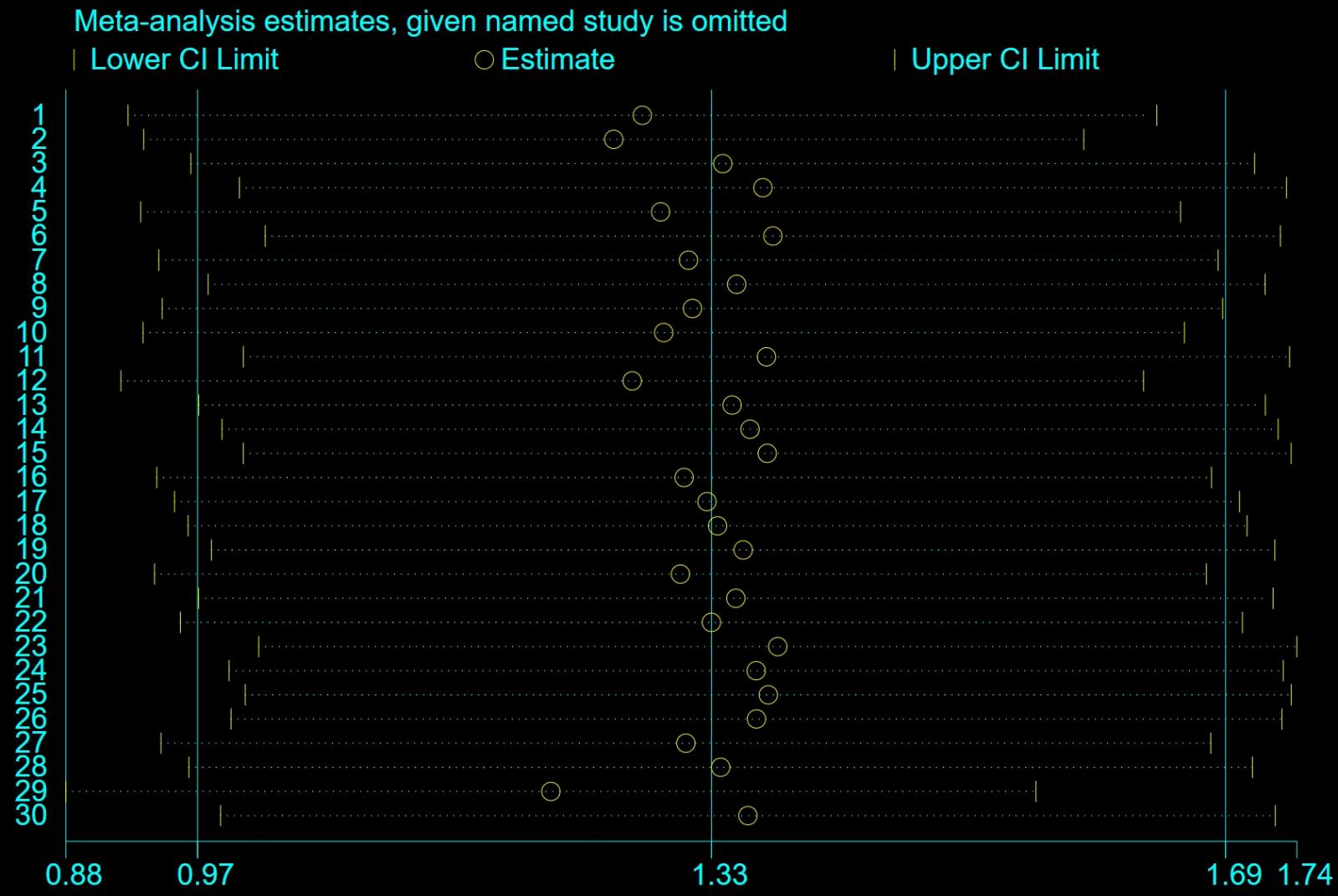


# Supplementary Figure 24. When ADL is used as the ending indicator, the robustness is tested using the one-by-one elimination method


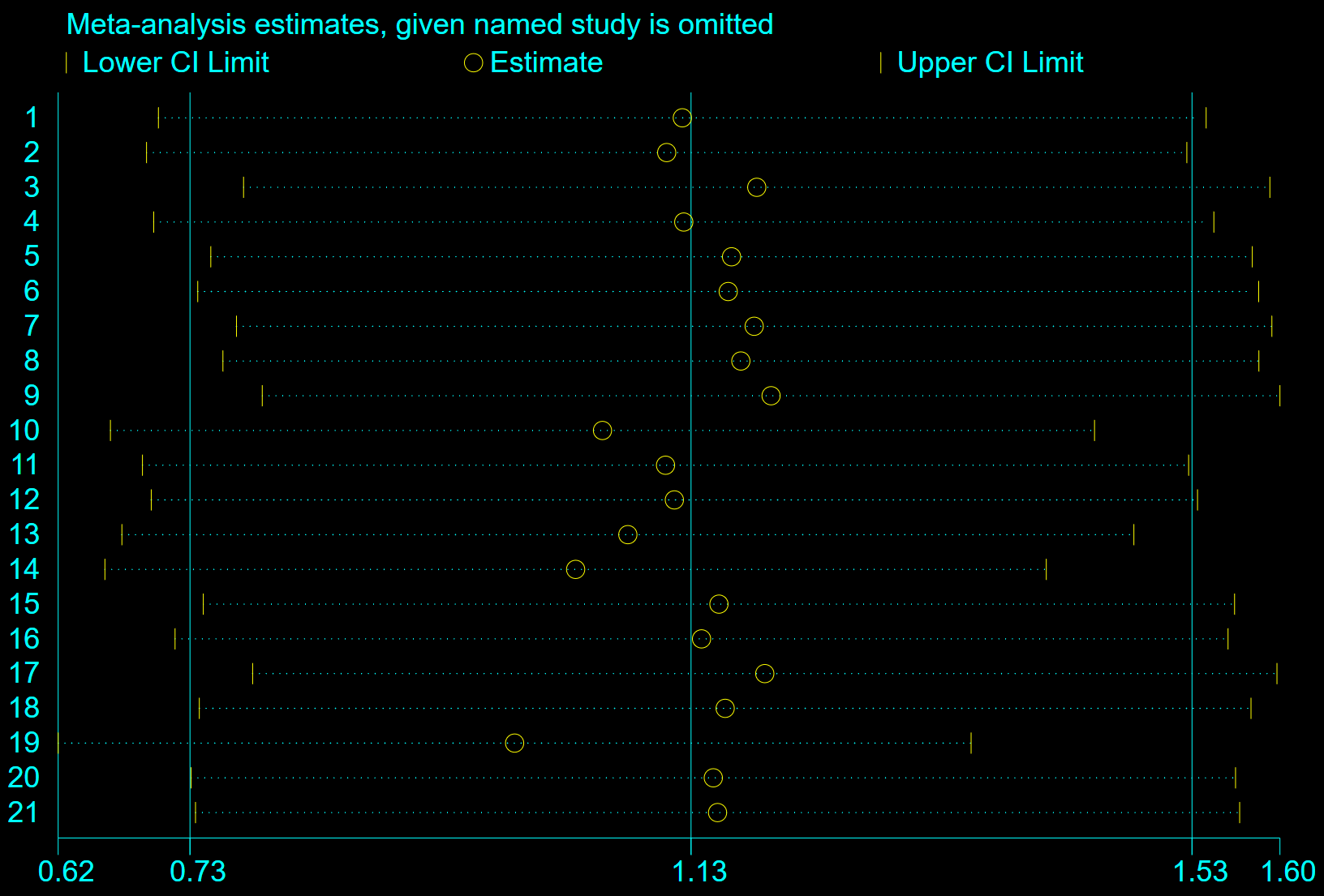


# Supplementary Figure 25. Network contribution maps under the upper limb functional indicators


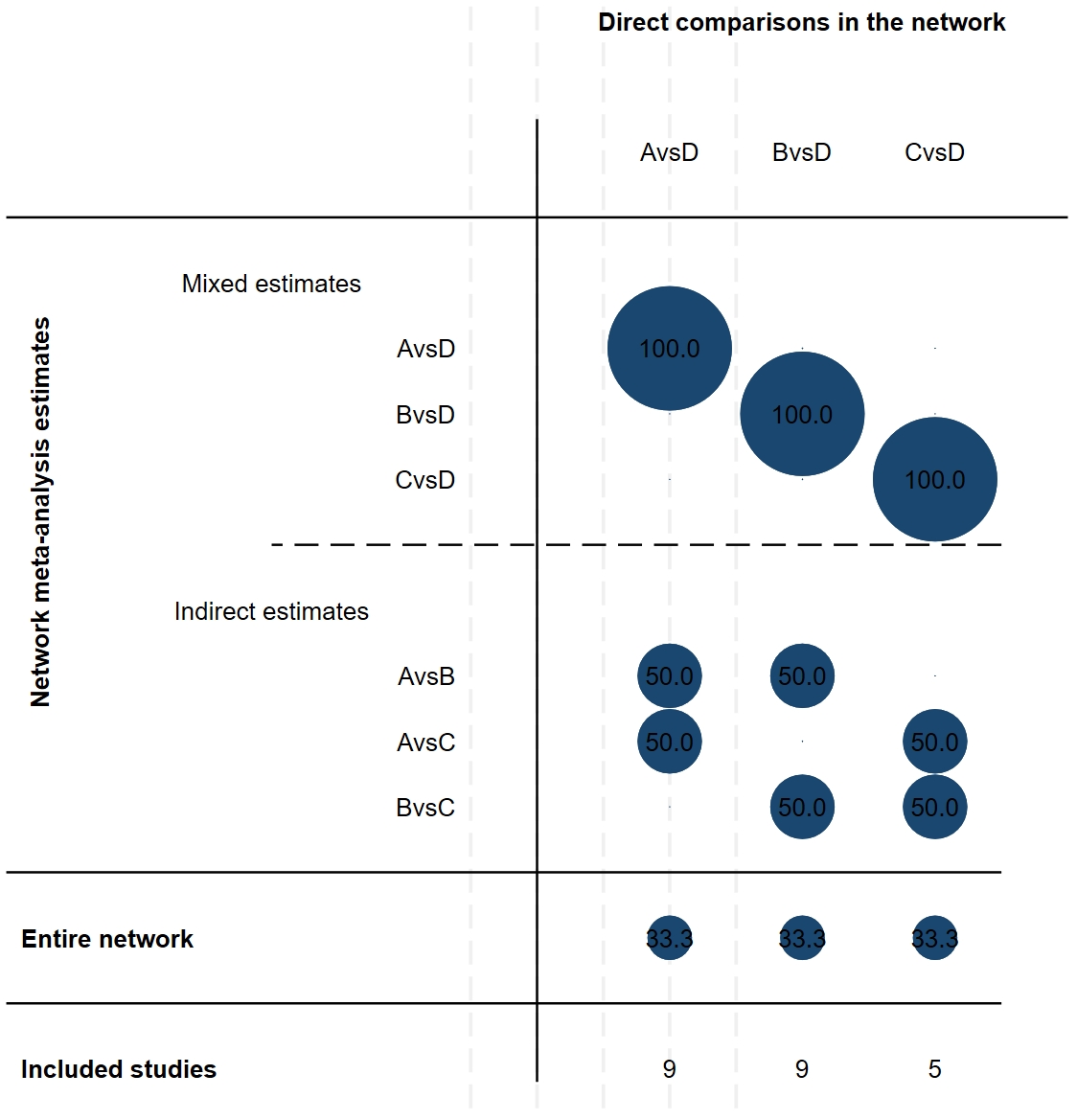


# Supplementary Figure 26. Network contribution map under lower limb functional indicators


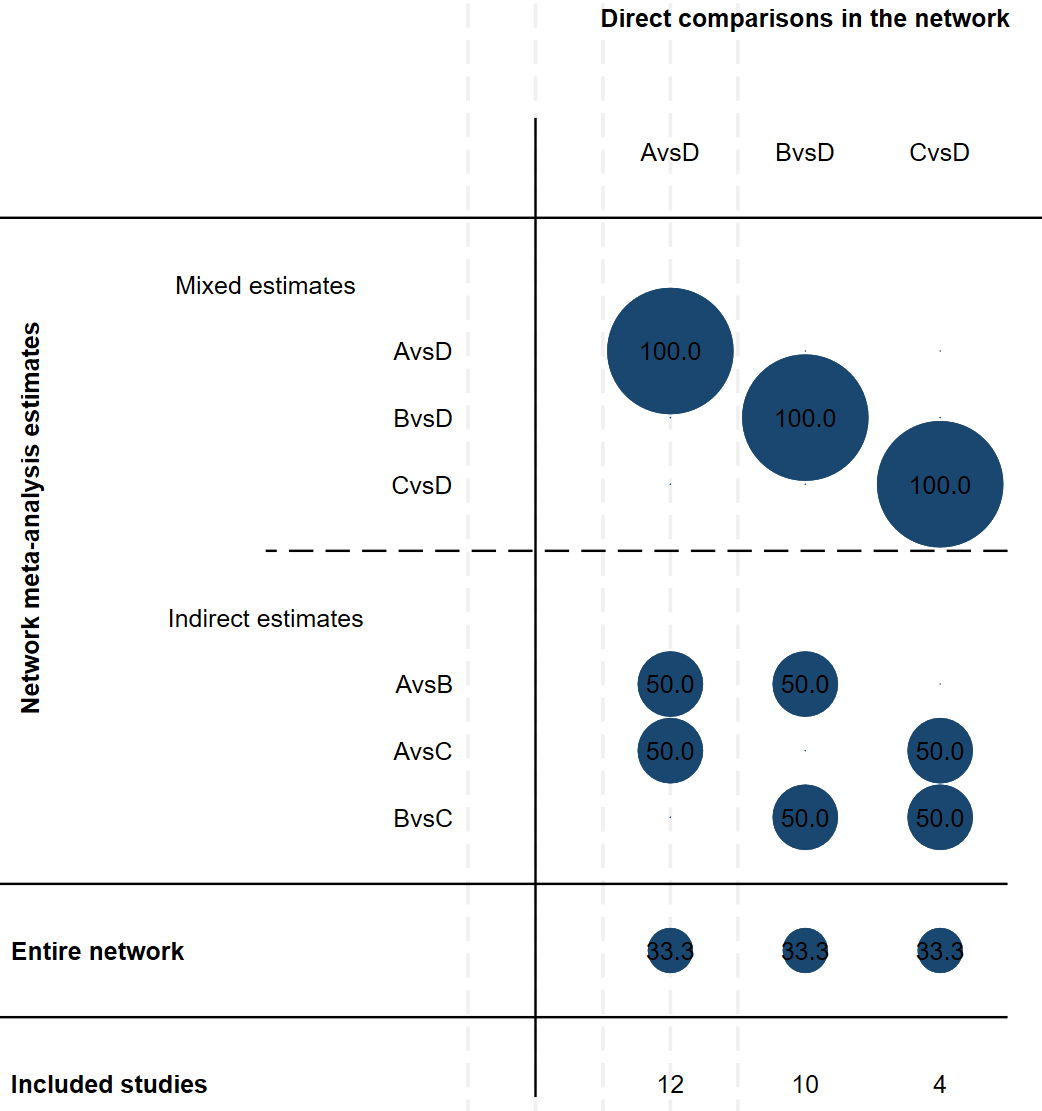


# Supplementary Figure 27. Network contributions under the balanced functional indicators


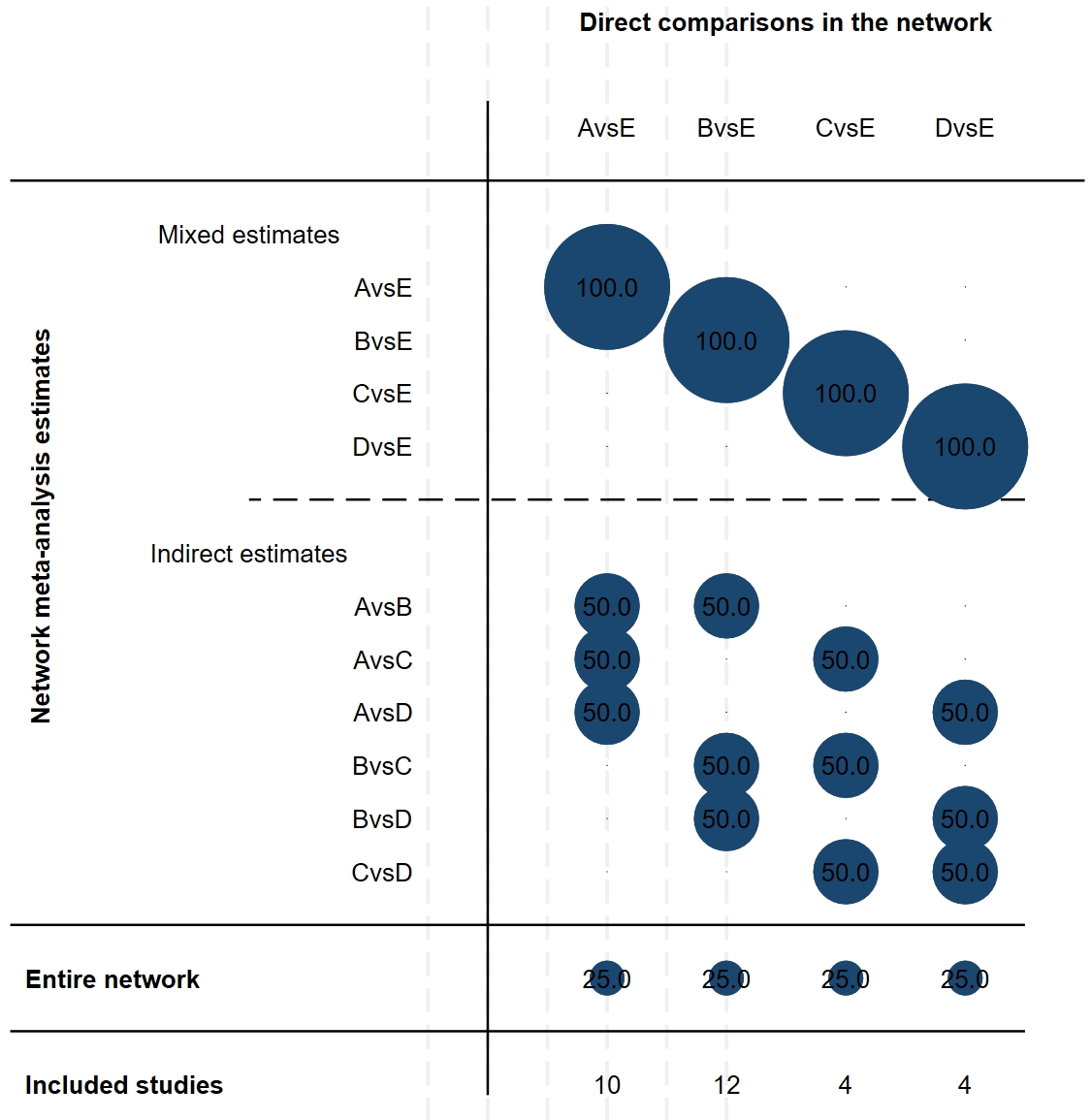


# Supplementary Figure 28. Network contribution map under ADL metrics


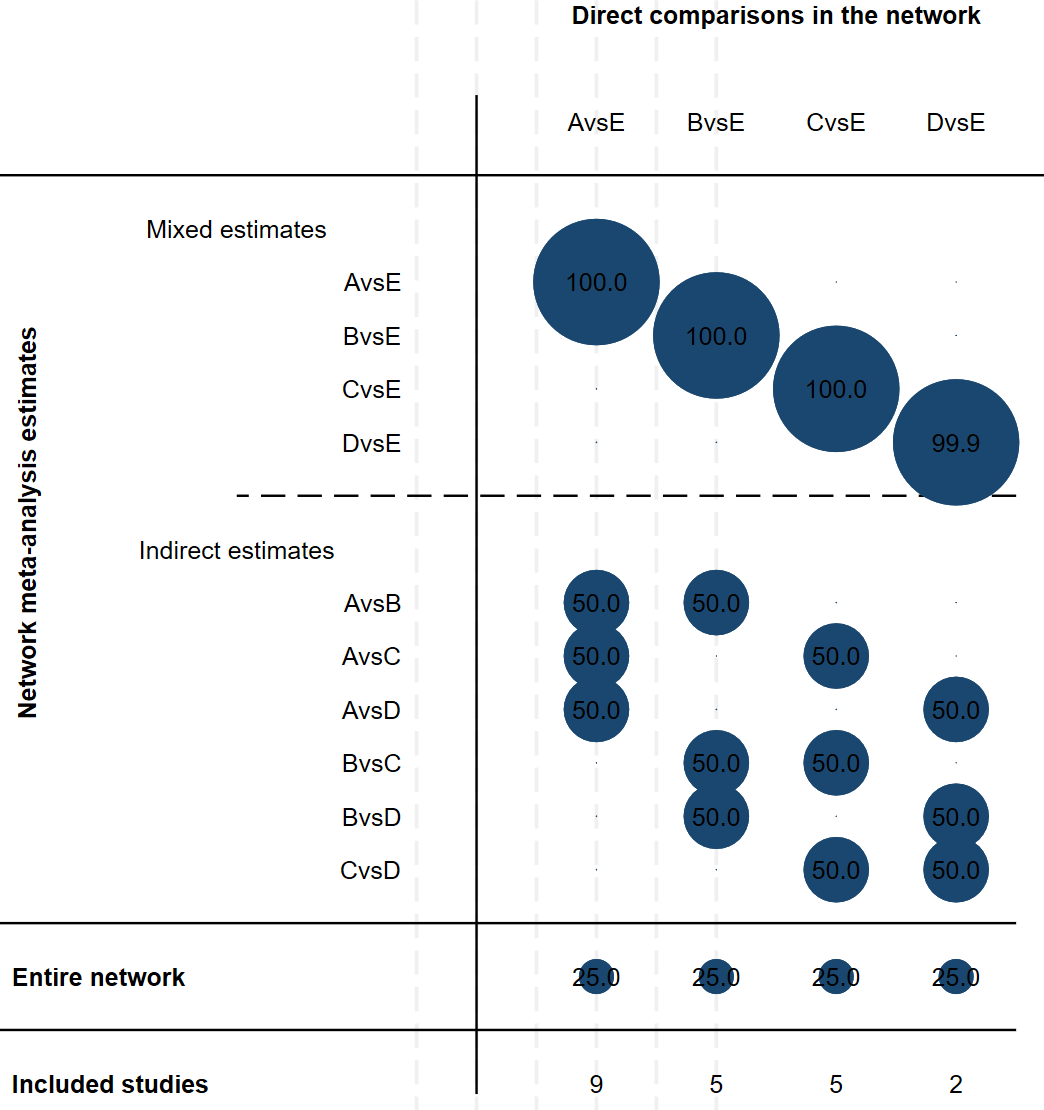


# 1.2 Supplementary Tables

# Supplementary Table 1. Checklist

| **Section/Topic** | **Item** | **Checklist Item** |  |
| --- | --- | --- | --- |
| **TITLE** |  |  |  |
| Title | 1 | Identify the report as a systematic review incorporating a network meta-analysis (or related form of meta-analysis). | Yes |
| **ABSTRACT** |  |  |  |
| Structured summary | 2 | Provide a structured summary including, as applicable: Background: main objectives Methods: data sources; study eligibility criteria, participants, and interventions; study appraisal; and synthesis methods, such as network meta-analysis. Results: number of studies and participants identified; summary estimates with corresponding confidence/credible intervals; treatment rankings may also be discussed. Authors may choose to summarize pairwise comparisons against a chosen treatment included in their analyses for brevity. Discussion/Conclusions: limitations; conclusions and implications of findings. Other: primary source of funding; systematic review registration number with registry name. | Yes |
| **INTRODUCTION** |  |  |  |
| Rationale | 3 | Describe the rationale for the review in the context of what is already known, including mention of why a network meta analysis has been conducted. | Yes |
| Objectives | 4 | Provide an explicit statement of questions being addressed, with reference to participants, interventions, comparisons, outcomes, and study design (PICOS). | Yes |
| **METHODS** |  |  |  |
| Protocol and registration | 5 | Indicate whether a review protocol exists and if and where it can be accessed (e.g., Web address); and, if available, provide registration information, including registration number. | Yes |
| Eligibility criteria | 6 | Specify study characteristics (e.g., PICOS, length of follow-up) and report characteristics (e.g., years considered, language, publication status) used as criteria for eligibility, giving rationale. Clearly describe eligible treatments included in the treatment network, and note whether any have been clustered or merged into the same node (with justification). | Yes |
| Information sources | 7 | Describe all information sources (e.g., databases with dates of coverage, contact with study authors to identify additional studies) in the search and date last searched. | Yes |
| Search | 8 | Present full electronic search strategy for at least one database, including any limits used, such that it could be repeated. | Yes |
| Study selection | 9 | State the process for selecting studies (i.e., screening, eligibility, included in systematic review, and, if applicable, included in the meta-analysis). | Yes |
| Data collection process | 10 | Describe method of data extraction from reports (e.g., piloted forms, independently, in duplicate) and any processes for obtaining and confirming data from investigators. | Yes |
| **Geometry of the network** | **S1** | Describe methods used to explore the geometry of the treatment network under study and potential biases related to it. This should include how the evidence base has been graphically summarized for presentation, and what characteristics were compiled and used to describe the evidence base to readers. | Yes |
| Risk of bias within individual studies | 12 | Describe methods used for assessing risk of bias of individual studies (including specification of whether this was done at the study or outcome level), and how this information is to be used in any data synthesis. | Yes |
| Summary measures | 13 | State the principal summary measures (e.g., risk ratio, difference in means). Also describe the use of additional summary measures assessed, such as treatment rankings and surface under the cumulative ranking curve (SUCRA) values, as well as modified approaches used to present summary findings from meta-analyses. | Yes |
| Planned methods of analysis | 14 | Describe the methods of handling data and combining results of studies for each network meta-analysis. This should include, but not be limited to: • Handling of multi-arm trials; • Selection of variance structure; • Selection of prior distributions in Bayesian analyses; and • Assessment of model fit. | Yes |
| **Assessment of Inconsistency** | **S2** | Describe the statistical methods used to evaluate the agreement of direct and indirect evidence in the treatment network(s) studied. Describe efforts taken to address its presence when found. | Yes |
| Risk of bias across studies | 15 | Specify any assessment of risk of bias that may affect the cumulative evidence (e.g., publication bias, selective reporting within studies). | Yes |
| Additional analyses | 16 | Describe methods of additional analyses if done, indicating which were pre-specified. This may include, but not be limited to, the following: • Sensitivity or subgroup analyses; • Meta-regression analyses; • Alternative formulations of the treatment network; and • Use of alternative prior distributions for Bayesian analyses (if applicable). | Yes |
| Study selection | 17 | Give numbers of studies screened, assessed for eligibility, and included in the review, with reasons for exclusions at each stage, ideally with a flow diagram. | Yes |
| **Presentation of network structure** | **S3** | Provide a network graph of the included studies to enable visualization of the geometry of the treatment network. | Yes |
| **Summary of network geometry** | **S4** | Provide a brief overview of characteristics of the treatment network. This may include commentary on the abundance of trials and randomized patients for the different interventions and pairwise comparisons in the network, gaps of evidence in the treatment network, and potential biases reflected by the network structure. | Yes |
| Study characteristics | 18 | For each study, present characteristics for which data were extracted (e.g., study size, PICOS, follow-up period. | Yes |
| Risk of bias within studies | 19 | Present data on risk of bias of each study and, if available, any outcome level assessment. | Yes |
| Results of individual studies | 20 | For all outcomes considered (benefits or harms), present, for each study: 1) simple summary data for each intervention group, and 2) effect estimates and confidence intervals. Modified approaches may be needed to deal with information from larger networks. | Yes |
| Synthesis of results | 21 | Present results of each meta-analysis done, including confidence/credible intervals. In larger networks, authors may focus on comparisons versus a particular comparator (e.g. placebo or standard care), with full findings presented in an appendix. League tables and forest plots may be considered to summarize pairwise comparisons. If additional summary measures were explored (such as treatment rankings), these should also be presented. | Yes |
| **Exploration for inconsistency** | **S5** | Describe results from investigations of inconsistency. This may include such information as measures of model fit to compare consistency and inconsistency models, P values from statistical tests, or summary of inconsistency estimates from different parts of the treatment network. | Yes |
| Risk of bias across studies | 22 | Present results of any assessment of risk of bias across studies for the evidence base being studied. | Yes |
| Results of additional analyses | 23 | Give results of additional analyses, if done (e.g., sensitivity or subgroup analyses, meta-regression analyses, alternative network geometries studied, alternative choice of prior distributions for Bayesian analyses, and so forth). | Yes |
| **DISCUSSION** |  |  |  |
| Summary of evidence | 24 | Summarize the main findings, including the strength of evidence for each main outcome; consider their relevance to key groups (e.g., healthcare providers, users, and policy makers). | Yes |
| Limitations | 25 | Discuss limitations at study and outcome level (e.g., risk of bias), and at review level (e.g., incomplete retrieval of identified research, reporting bias). Comment on the validity of the assumptions, such as transitivity and consistency. Comment on any concerns regarding network geometry (e.g., avoidance of certain comparisons). | Yes |
| Conclusions | 26 | Provide a general interpretation of the results in the context of other evidence, and implications for future research. | Yes |
| **FUNDING** |  |  |  |
| Funding | 27 | Describe sources of funding for the systematic review and other support (e.g., supply of data); role of funders for the systematic review. This should also include information regarding whether funding has been received from manufacturers of treatments in the network and/or whether some of the authors are content experts with professional conflicts of interest that could affect use of treatments in the network. | Yes |

# Supplementary Table 2. Searching strategies

| **PubMed** | |
| --- | --- |
| #1 | "Stroke"[MeSH Terms] |
| #2 | "tai ji"[MeSH Terms] |
| #3 | "Tai-ji"[Title/Abstract] OR "Tai Chi"[Title/Abstract] OR "chi tai"[Title/Abstract] OR "Tai Chi Chuan"[Title/Abstract] OR "Taiji"[Title/Abstract] OR "Taijiquan"[Title/Abstract] OR "T'ai Chi"[Title/Abstract] OR "Tai Ji Quan"[Title/Abstract] OR "ji quan tai"[Title/Abstract] OR "quan tai ji"[Title/Abstract] |
| #4 | "baduanjin"[Title/Abstract] |
| #5 | "qigong"[MeSH Terms] |
| #6 | (("qigong"[MeSH Terms] OR "qigong"[All Fields] OR ("ch i"[All Fields] AND "kung"[All Fields]) OR "ch i kung"[All Fields]) AND "qi gong"[Title/Abstract]) OR "qi gong"[Title/Abstract] |
| #7 | "yijinjing"[Title/Abstract] |
| #8 | "wuqinxi"[Title/Abstract] |
| #9 | "martial arts"[MeSH Terms] |
| #10 | "arts martial"[Title/Abstract] OR "Judo"[Title/Abstract] OR "Karate"[Title/Abstract] OR "Kung Fu"[Title/Abstract] OR "Gongfu"[Title/Abstract] OR "Gong Fu"[Title/Abstract] OR "fu gong"[Title/Abstract] OR "Tae Kwon Do"[Title/Abstract] OR "Wushu"[Title/Abstract] OR "Aikido"[Title/Abstract] OR "Jujitsu"[Title/Abstract] |
| #11 | "Tai-ji"[MeSH Terms] OR ("Tai-ji"[Title/Abstract] OR "Tai Chi"[Title/Abstract] OR "chi tai"[Title/Abstract] OR "Tai Chi Chuan"[Title/Abstract] OR "Taiji"[Title/Abstract] OR "Taijiquan"[Title/Abstract] OR "T'ai Chi"[Title/Abstract] OR "Tai Ji Quan"[Title/Abstract] OR "ji quan tai"[Title/Abstract] OR "quan tai ji"[Title/Abstract]) OR "baduanjin"[Title/Abstract] OR "qigong"[MeSH Terms] OR ((("qigong"[MeSH Terms] OR "qigong"[All Fields] OR ("ch i"[All Fields] AND "kung"[All Fields]) OR "ch i kung"[All Fields]) AND "qi gong"[Title/Abstract]) OR "qi gong"[Title/Abstract]) OR "yijinjing"[Title/Abstract] OR "wuqinxi"[Title/Abstract] OR "martial arts"[MeSH Terms] OR ("arts martial"[Title/Abstract] OR "Judo"[Title/Abstract] OR "Karate"[Title/Abstract] OR "Kung Fu"[Title/Abstract] OR "Gongfu"[Title/Abstract] OR "Gong Fu"[Title/Abstract] OR "fu gong"[Title/Abstract] OR "Tae Kwon Do"[Title/Abstract] OR "Wushu"[Title/Abstract] OR "Aikido"[Title/Abstract] OR "Jujitsu"[Title/Abstract]) |
| #12 | "Strokes"[Title/Abstract] OR "Cerebrovascular Accident"[Title/Abstract] OR "Cerebrovascular Accidents"[Title/Abstract] OR "Cerebral Stroke"[Title/Abstract] OR "Cerebral Strokes"[Title/Abstract] OR "stroke cerebral"[Title/Abstract] OR "strokes cerebral"[Title/Abstract] OR "Cerebrovascular Apoplexy"[Title/Abstract] OR "apoplexy cerebrovascular"[Title/Abstract] OR "vascular accident brain"[Title/Abstract] OR "Brain Vascular Accident"[Title/Abstract] OR "Brain Vascular Accidents"[Title/Abstract] OR "Cerebrovascular Stroke"[Title/Abstract] OR "Cerebrovascular Strokes"[Title/Abstract] OR "stroke cerebrovascular"[Title/Abstract] OR "strokes cerebrovascular"[Title/Abstract] OR "Apoplexy"[Title/Abstract] OR "cva cerebrovascular accident"[Title/Abstract] OR "stroke acute"[Title/Abstract] OR "Acute Stroke"[Title/Abstract] OR "Acute Strokes"[Title/Abstract] OR "strokes acute"[Title/Abstract] OR "cerebrovascular accident acute"[Title/Abstract] OR "Acute Cerebrovascular Accident"[Title/Abstract] OR "Acute Cerebrovascular Accidents"[Title/Abstract] OR "cerebrovascular accidents acute"[Title/Abstract] |
| #13 | "Strokes"[Title/Abstract] OR "Cerebrovascular Accident"[Title/Abstract] OR "Cerebrovascular Accidents"[Title/Abstract] OR "Cerebral Stroke"[Title/Abstract] OR "Cerebral Strokes"[Title/Abstract] OR "stroke cerebral"[Title/Abstract] OR "strokes cerebral"[Title/Abstract] OR "Cerebrovascular Apoplexy"[Title/Abstract] OR "apoplexy cerebrovascular"[Title/Abstract] OR "vascular accident brain"[Title/Abstract] OR "Brain Vascular Accident"[Title/Abstract] OR "Brain Vascular Accidents"[Title/Abstract] OR "Cerebrovascular Stroke"[Title/Abstract] OR "Cerebrovascular Strokes"[Title/Abstract] OR "stroke cerebrovascular"[Title/Abstract] OR "strokes cerebrovascular"[Title/Abstract] OR "Apoplexy"[Title/Abstract] OR "cva cerebrovascular accident"[Title/Abstract] OR "stroke acute"[Title/Abstract] OR "Acute Stroke"[Title/Abstract] OR "Acute Strokes"[Title/Abstract] OR "strokes acute"[Title/Abstract] OR "cerebrovascular accident acute"[Title/Abstract] OR "Acute Cerebrovascular Accident"[Title/Abstract] OR "Acute Cerebrovascular Accidents"[Title/Abstract] OR "cerebrovascular accidents acute"[Title/Abstract] OR "Stroke"[MeSH Terms] |
| #14 | "randomized controlled trial"[Publication Type] OR "randomized"[Title/Abstract] OR "placebo"[Title/Abstract] |
| #15 | ("randomized controlled trial"[Publication Type] OR "randomized"[Title/Abstract] OR "placebo"[Title/Abstract]) AND ("Strokes"[Title/Abstract] OR "Cerebrovascular Accident"[Title/Abstract] OR "Cerebrovascular Accidents"[Title/Abstract] OR "Cerebral Stroke"[Title/Abstract] OR "Cerebral Strokes"[Title/Abstract] OR "stroke cerebral"[Title/Abstract] OR "strokes cerebral"[Title/Abstract] OR "Cerebrovascular Apoplexy"[Title/Abstract] OR "apoplexy cerebrovascular"[Title/Abstract] OR "vascular accident brain"[Title/Abstract] OR "Brain Vascular Accident"[Title/Abstract] OR "Brain Vascular Accidents"[Title/Abstract] OR "Cerebrovascular Stroke"[Title/Abstract] OR "Cerebrovascular Strokes"[Title/Abstract] OR "stroke cerebrovascular"[Title/Abstract] OR "strokes cerebrovascular"[Title/Abstract] OR "Apoplexy"[Title/Abstract] OR "cva cerebrovascular accident"[Title/Abstract] OR "stroke acute"[Title/Abstract] OR "Acute Stroke"[Title/Abstract] OR "Acute Strokes"[Title/Abstract] OR "strokes acute"[Title/Abstract] OR "cerebrovascular accident acute"[Title/Abstract] OR "Acute Cerebrovascular Accident"[Title/Abstract] OR "Acute Cerebrovascular Accidents"[Title/Abstract] OR "cerebrovascular accidents acute"[Title/Abstract] OR "Stroke"[MeSH Terms]) AND ("Tai-ji"[MeSH Terms] OR ("Tai-ji"[Title/Abstract] OR "Tai Chi"[Title/Abstract] OR "chi tai"[Title/Abstract] OR "Tai Chi Chuan"[Title/Abstract] OR "Taiji"[Title/Abstract] OR "Taijiquan"[Title/Abstract] OR "T'ai Chi"[Title/Abstract] OR "Tai Ji Quan"[Title/Abstract] OR "ji quan tai"[Title/Abstract] OR "quan tai ji"[Title/Abstract]) OR "baduanjin"[Title/Abstract] OR "qigong"[MeSH Terms] OR ((("qigong"[MeSH Terms] OR "qigong"[All Fields] OR ("ch i"[All Fields] AND "kung"[All Fields]) OR "ch i kung"[All Fields]) AND "qi gong"[Title/Abstract]) OR "qi gong"[Title/Abstract]) OR "yijinjing"[Title/Abstract] OR "wuqinxi"[Title/Abstract] OR "martial arts"[MeSH Terms] OR ("arts martial"[Title/Abstract] OR "Judo"[Title/Abstract] OR "Karate"[Title/Abstract] OR "Kung Fu"[Title/Abstract] OR "Gongfu"[Title/Abstract] OR "Gong Fu"[Title/Abstract] OR "fu gong"[Title/Abstract] OR "Tae Kwon Do"[Title/Abstract] OR "Wushu"[Title/Abstract] OR "Aikido"[Title/Abstract] OR "Jujitsu"[Title/Abstract])) |
| **Web of Science** | |
| #1 | stroke (Topic) OR Cerebrovascular Accident (Topic) OR Cerebrovascular Accidents (Topic) AND Brain Vascular Accident (Topic) AND Cerebrovascular Apoplexy (Topic) AND Acute Cerebrovascular Accidents (Topic) AND CVA (Cerebrovascular Accident) (Topic) and Preprint Citation Index (Exclude – Database) |
| #2 | Tai Ji (Topic) OR Tai-ji (Topic) OR Tai Chi (Topic) AND Tai Chi Chuan (Topic) AND Ji Quan, Tai (Topic) AND Quan, Tai Ji (Topic) AND Taijiquan (Topic) and Preprint Citation Index (Exclude – Database) |
| #3 | baduanjin (Topic) and Preprint Citation Index (Exclude – Database) |
| #4 | Qigong (Topic) OR Ch'i Kung (Topic) OR Qi Gong (Topic) and Preprint Citation Index (Exclude – Database) |
| #5 | yijinjing (Topic) and Preprint Citation Index (Exclude – Database) |
| #6 | wuqinxi (Topic) and Preprint Citation Index (Exclude – Database) |
| #7 | Martial Arts (Topic) OR Arts, Martial (Topic) OR Judo (Topic) AND Karate (Topic) AND Kung Fu (Topic) AND Gongfu (Topic) AND Tae Kwon Do (Topic) AND Wushu (Topic) AND Hap Ki Do (Topic) AND Aikido (Topic) AND Jujitsu (Topic) and Preprint Citation Index (Exclude – Database) |
| #8 | randomized controlled trial (Topic) OR controlled trial, randomized (Topic) OR randomised controlled study (Topic) AND randomised controlled trial (Topic) AND randomized controlled study (Topic) AND trial, randomized controlled (Topic) AND randomized controlled trial (Topic) and Preprint Citation Index (Exclude – Database) |
| #9 | #2 OR #3 OR #4 OR #5 OR #6 OR #7 and Preprint Citation Index (Exclude – Database) |
| #10 | #9 AND #8 AND #1 and Preprint Citation Index (Exclude – Database) |
| **Embase** | |
| #1 | 'cerebrovascular accident'/exp |
| #2 | 'accident, cerebrovascular':ti,ab,kw OR 'acute cerebrovascular lesion':ti,ab,kw OR 'acute focal cerebral vasculopathy':ti,ab,kw OR 'acute stroke':ti,ab,kw OR 'apoplectic stroke':ti,ab,kw OR 'apoplexia':ti,ab,kw OR 'apoplexy':ti,ab,kw OR 'blood flow disturbance, brain':ti,ab,kw OR 'brain accident':ti,ab,kw OR 'brain attack':ti,ab,kw OR 'brain blood flow disturbance':ti,ab,kw OR 'brain insult':ti,ab,kw OR 'brain insultus':ti,ab,kw OR 'brain vascular accident':ti,ab,kw OR 'cerebral apoplexia':ti,ab,kw OR 'cerebral insult':ti,ab,kw OR 'cerebral stroke':ti,ab,kw OR 'cerebral vascular accident':ti,ab,kw OR 'cerebral vascular insufficiency':ti,ab,kw OR 'cerebro vascular accident':ti,ab,kw OR 'cerebrovascular arrest':ti,ab,kw OR 'cerebrovascular failure':ti,ab,kw OR 'cerebrovascular injury':ti,ab,kw OR 'cerebrovascular insufficiency':ti,ab,kw OR 'cerebrovascular insult':ti,ab,kw OR 'cerebrum vascular accident':ti,ab,kw OR 'cryptogenic stroke':ti,ab,kw OR 'cva':ti,ab,kw OR 'insultus cerebralis':ti,ab,kw OR 'ischaemic seizure':ti,ab,kw OR 'ischemic seizure':ti,ab,kw OR 'stroke':ti,ab,kw OR 'thrombotic stroke':ti,ab,kw OR 'cerebrovascular accident':ti,ab,kw |
| #3 | 'tai chi'/exp |
| #4 | 'tai chi chuan':ti,ab,kw OR 'tai ji':ti,ab,kw OR 'taiji quan':ti,ab,kw OR 'taijiquan':ti,ab,kw OR 'tai chi':ti,ab,kw |
| #5 | 'buduanjin' |
| #6 | 'qigong'/exp |
| #7 | 'chi kung':ti,ab,kw OR 'chigung':ti,ab,kw OR 'qi gong':ti,ab,kw OR 'qigong':ti,ab,kw |
| #8 | 'yi jinjing' |
| #9 | 'wu qinxi' |
| #10 | 'martial art'/exp |
| #11 | 'martial arts':ti,ab,kw OR 'martial sport':ti,ab,kw OR 'martial art':ti,ab,kw |
| #12 | 'randomized controlled trial'/exp |
| #13 | 'controlled trial, randomized':ti,ab,kw OR 'randomised controlled study':ti,ab,kw OR 'randomised controlled trial':ti,ab,kw OR 'randomized controlled study':ti,ab,kw OR 'trial, randomized controlled':ti,ab,kw OR 'randomized controlled trial':ti,ab,kw |
| #14 | #1 OR #2 |
| #15 | #3 OR #4 OR #5 OR #6 OR #7 OR #8 OR #9 OR #10 OR #11 |
| #16 | #12 OR #13 |
| #17 | #14 AND #15 AND #16 |
| **scopus** | |
|  | ( TITLE-ABS-KEY ( "Stroke" OR "Strokes" OR "Cerebrovascular Accident" OR "Cerebrovascular Accidents" OR "Cerebral Stroke" OR "Cerebral Strokes" OR "Stroke, Cerebral" OR "Strokes, Cerebral" OR "Cerebrovascular Apoplexy" OR "Apoplexy, Cerebrovascular" OR "Vascular Accident, Brain" OR "Brain Vascular Accident" OR "Brain Vascular Accidents" OR "Vascular Accidents, Brain" OR "Cerebrovascular Stroke" OR "Cerebrovascular Strokes" OR "Stroke, Cerebrovascular" OR "Strokes, Cerebrovascular" OR "Apoplexy" OR "CVA (Cerebrovascular Accident)" OR "CVAs (Cerebrovascular Accident)" OR "Stroke, Acute" OR "Acute Stroke" OR "Acute Strokes" OR "Strokes, Acute" OR "Cerebrovascular Accident, Acute" OR "Acute Cerebrovascular Accident" OR "Acute Cerebrovascular Accidents" OR "Cerebrovascular Accidents, Acute" ) ) AND ( TITLE-ABS-KEY ( "RCT" OR "randomised controlled trial" OR "randomised controlled trials" OR "randomised controlled" ) ) AND ( ( TITLE-ABS-KEY ( "Martial Arts" OR "Arts, Martial" OR "Judo" OR "Karate" OR "Kung Fu" OR "Gongfu" OR "Gong Fu" OR "Fu, Gong" OR "Tae Kwon Do" OR "Wushu" OR "Hap Ki Do" OR "Aikido" OR "Jujitsu" ) ) OR ( TITLE-ABS-KEY ( "wuqinxi" ) ) OR ( TITLE-ABS-KEY ( "yijinjing" ) ) OR ( TITLE-ABS-KEY ( "Qigong" OR "Ch'i Kung" OR "Qi Gong" ) ) OR ( TITLE-ABS-KEY ( "baduanjin" ) ) OR ( TITLE-ABS-KEY ( "Tai Ji" OR "Tai-ji" OR "Tai Chi" OR "Chi, Tai" OR "Tai Chi Chuan" OR "Taiji" OR "Taijiquan" OR "T'ai Chi" OR "Tai Ji Quan" OR "Ji Quan, Tai" OR "Quan, Tai Ji" ) ) ) |
| **The Cochrane Library** | |
| #1 | MeSH descriptor: [Stroke] explode all trees |
| #2 | ('Cerebrovascular Stroke' OR 'Cerebrovascular Accident' OR 'Apoplexy' OR 'CVA (Cerebrovascular Accident)' OR 'Cerebral Stroke' OR 'Apoplexy, Cerebrovascular' OR 'Vascular Accidents, Brain' OR 'Brain Vascular Accident' OR 'Cerebrovascular Strokes' OR 'Cerebrovascular Apoplexy' OR 'Cerebrovascular Accidents' OR 'Strokes, Cerebrovascular' OR 'Vascular Accident, Brain' OR 'Strokes' OR 'Brain Vascular Accidents' OR 'Strokes, Cerebral' OR 'Stroke, Cerebral' OR 'CVAs (Cerebrovascular Accident)' OR 'Cerebral Strokes' OR 'Stroke, Cerebrovascular' OR 'Stroke, Acute' OR 'Acute Cerebrovascular Accident' OR 'Strokes, Acute' OR 'Acute Strokes' OR 'Cerebrovascular Accident, Acute' OR 'Acute Stroke' OR 'Acute Cerebrovascular Accidents' OR 'Cerebrovascular Accidents, Acute'):ti,kw,ab |
| #3 | MeSH descriptor: [Tai Ji] explode all trees |
| #4 | ('T'ai Chi' OR 'Chi, Tai' OR 'Tai Chi Chuan' OR 'Ji Quan, Tai' OR 'Taijiquan' OR 'Tai Ji Quan' OR 'Tai-ji' OR 'Tai Chi' OR 'Quan, Tai Ji'):ti,kw,ab |
| #5 | (baduanjin):ti,kw,ab |
| #6 | MeSH descriptor: [Qigong] explode all trees |
| #7 | ('Ch'i Kung' OR 'Qi Gong '):ti,kw,ab |
| #8 | ('yi jinjing'):ti,kw,ab |
| #9 | ('wu qinxi'):ti,kw,ab |
| #10 | MeSH descriptor: [Martial Arts] explode all trees |
| #11 | ('Karate' OR 'Arts, Martial' OR 'Judo' OR 'Aikido' OR 'Tae Kwon Do' OR 'Jujitsu' OR 'Gongfu' OR 'Fu, Gong' OR 'Kung Fu' OR 'Gong Fu' OR 'Hap Ki Do' OR 'Wushu '):ti,kw,ab |
| #12 | ('randomized controlled trial' OR 'randomized' OR 'placebo'):ti,kw,ab |
| #13 | #1 OR #2 |
| #14 | #3 OR #4 OR #5 OR #6 OR #7 OR #8 OR #9 OR #10 OR #11 |
| #15 | #13 AND #14 AND #12 |
| **China National Knowledge Infrastructure** | |
|  | （篇关摘：太极 + 八段锦 + 气功 + 易筋经 + 五禽戏 + 武术 + 国术(模糊)）AND（篇关摘：中风 + 脑卒中 + 缺血性脑卒中(模糊)）AND（篇关摘：随机对照试验 + 随机对照研究 + 随机对照(模糊)） |
| **Wanfang Data** | |
|  | 全部:(太极 or 八段锦 or 气功 or 易筋经 or 五禽戏 or 武术 or 国术) and 全部:(中风 or 脑卒中 or 缺血性脑卒中) and 全部:(随机对照试验 or 随机对照研究) |
| **VIP** | |
|  | ((((((((任意字段=太极 OR 任意字段=八段锦) OR 任意字段=气功) OR 任意字段=易筋经) OR 任意字段=五禽戏) OR 任意字段=武术) OR 任意字段=国术) AND ((任意字段=中风 OR 任意字段=脑卒中) OR 任意字段=缺血性脑卒中)) AND ((任意字段=随机对照试验 OR 任意字段=随机对照研究) OR 任意字段=rct)) |
| **Chinese Biomedical Literature Database** | |
|  | ( "太极"[全部字段:智能] OR "八段锦"[全部字段:智能] OR "气功"[全部字段:智能] OR "易筋经"[全部字段:智能] OR "五禽戏"[全部字段:智能] OR "国术"[全部字段:智能]) AND ( "中风"[全部字段:智能] OR "脑卒中"[全部字段:智能] OR "缺血性脑卒中"[全部字段:智能]) AND ( "随机对照试验"[全部字段:智能] OR "随机对照研究"[全部字段:智能]) |

# Supplementary Table 3. Results of egger’s test

| **Outcomes** | **Egger’s test** |
| --- | --- |
| Upper limb function | *P=*0.0789 |
| Lower limb function | *P=*0.0863 |
| Balance function | *P=*0.1098 |
| ADL | *P=*0.2918 |

# Supplementary Table 4. Conduct subgroup analysis based on prespecified moderating variables (Intervention cycle, Exercise frequency, Patient age)

| **Control variables** | | **Endpoint Indicator** | **Studies** | **SMD [95%CI]** | **I^2^** | ***P*** |
| --- | --- | --- | --- | --- | --- | --- |
| **Intervention cycle** | ≤4 weeks | Upper limb function | 6 | 0.81[0.48-1.14] | 38.4% | ＜0.05 |
|  | 5-8 weeks | Upper limb function | 6 | 1.36[0.55-2.16] | 92.5% | ＜0.05 |
|  | ＞8 weeks | Upper limb function | 7 | 1.33[0.67-1.98] | 92.9% | ＜0.05 |
|  | ≤4 weeks | Lower limb function | 4 | 0.59[0.21-0.97] | 47.7% | ＜0.05 |
|  | 5-8 weeks | Lower limb function | 7 | 1.38[0.55-2.21] | 94.1% | ＜0.05 |
|  | ＞8 weeks | Lower limb function | 8 | 1.44[0.78-2.10] | 93.8% | ＜0.05 |
|  | ≤4 weeks | Balance function | 7 | 1.17[0.31-2.04] | 92.3% | ＜0.05 |
|  | 5-8 weeks | Balance function | 6 | 1.66[0.76-2.57] | 94.2% | ＜0.05 |
|  | ＞8 weeks | Balance function | 14 | 1.12[0.69-1.55] | 90.3% | ＜0.05 |
|  | ≤4 weeks | ADL | 7 | 1.18[0.30-2.06] | 91.3% | ＜0.05 |
|  | 5-8 weeks | ADL | 4 | 0.98[-0.17-2.13] | 94.7% | ＞0.05 |
|  | ＞8 weeks | ADL | 8 | 1.11[0.48-1.74] | 93.2% | ＜0.05 |
| **Exercise frequency** | ≤ three times a week | Upper limb function | 4 | 0.70[0.42-0.98] | 0.0% | ＜0.05 |
|  | ＞ three times a week | Upper limb function | 10 | 0.99[0.54-1.43] | 86.6% | ＜0.05 |
|  | ≤ three times a week | Lower limb function | 6 | 0.71[0.10-1.31] | 85.9% | ＜0.05 |
|  | ＞ three times a week | Lower limb function | 7 | 0.89[0.38-1.39] | 89.1% | ＜0.05 |
|  | ≤ three times a week | Balance function | 5 | 1.06[0.26-1.86] | 88.3% | ＜0.05 |
|  | ＞ three times a week | Balance function | 17 | 1.19[0.66-1.72] | 94.3% | ＜0.05 |
|  | ≤ three times a week | ADL | 4 | 0.64[0.07-1.22] | 77.8% | ＜0.05 |
|  | ＞ three times a week | ADL | 12 | 1.14[0.50-1.78] | 93.8% | ＜0.05 |
| **Patient age** | ＜ 60 years old | Upper limb function | 11 | 0.95[0.61-1.28] | 74.1% | ＜0.05 |
|  | ≥ 60 years old | Upper limb function | 11 | 1.52[0.95-2.09] | 93.3% | ＜0.05 |
|  | ＜ 60 years old | Lower limb function | 9 | 0.94[0.57-1.31] | 78.3% | ＜0.05 |
|  | ≥ 60 years old | Lower limb function | 16 | 1.41[0.93-1.88] | 92.9% | ＜0.05 |
|  | ＜ 60 years old | Balance function | 13 | 1.49[0.89-2.08] | 91.0% | ＜0.05 |
|  | ≥ 60 years old | Balance function | 16 | 1.10[0.71-1.49] | 89.8% | ＜0.05 |
|  | ＜ 60 years old | ADL | 10 | 1.12[0.53-1.71] | 88.5% | ＜0.05 |
|  | ≥ 60 years old | ADL | 11 | 1.14[0.57-1.70] | 93.7% | ＜0.05 |

# Supplementary Table 5. Table of included trials in the systematic review with detailed study characteristics

| Number | Author | Year | Patients | | Age(year) | | Treatment（Male/Female） | Treatment | | Intervention period | Outcome indicator |
| --- | --- | --- | --- | --- | --- | --- | --- | --- | --- | --- | --- |
|  |  |  | I | C | I | C |  | I | C |  |  |
| 1 | Zhang, Jing | 2010 | 16 | 16 | —— | | —— | YJJ+CT | CT | —— | FMA-UE、FMA-LE |
| 2 | Bai, Yanjie | 2011 | 30 | 30 | 53.7±4.5 | 51.3±7.5 | I（20/10）；C（22/8） | BDJ | CT | 6W,14T/W | BBS |
| 3 | Zhang, Ming | 2013 | 115 | 106 | 33-82 | | 117，104 | BDJ | CT | 6W,7T/W | BBS |
| 4 | Fu, Changxi | 2016 | 30 | 30 | 59.7±7.6 | 60.3±8.4 | I（19/11）；C（18/12） | TJ+CT | CT | 8W,6T/W | BBS |
| 5 | Wang, Xiangbin | 2016 | 14 | 16 | 60.71±7.32 | 58.56±8.52 | I（9/5）；C（14/2） | TJ+CT | CT | 12W,5T/W | BBS |
| 6 | Yang, Zhibo | 2016 | 30 | 30 | 58.00±11.27 | 60.07±7.87 | I（20/10）；C（11/9） | TJ+CT | CT | —— | FMA-LE、BI |
| 7 | Guan, Ling | 2017 | 30 | 30 | 70.67±5.74 | 70.03±5.92 | I（18/12）；C（20/10） | WQX+CT | CT | 12W,5T/W | BBS、BI |
| 8 | Sun, Pingping | 2017 | 30 | 30 | 63.73±6.37 | 64.37±5.74 | I（16/14）；C（20/10） | YJJ+CT | CT | 3W,3T/W | FMA、BI |
| 9 | Tian, Hua | 2017 | 30 | 30 | 54.3±4.7 | 53±4.3 | I（17/13）；C（19/11） | BDJ | CT | 14W,2T/W | BBS |
| 10 | Xie, G | 2018 | 120 | 124 | 60.9±8.7 | 60.1±8.6 | I（83/37）；C（99/25） | TJ | CT | 12W,5T/W | BBS、FMA、MBI |
| 11 | Cui, Yongsheng | 2018 | 24 | 19 | 53.67±12.98 | 55.33±14.32 | I（15/9）；C（12/7） | BDJ | CT | 8W,3T/W | FMA |
| 12 | Jiang, Suzhen | 2018 | 30 | 30 | 58.8±11.7 | 56.46±12.81 | I（23/7）；C（22/8） | TJ+CT | CT | 8W,10T/W | FMA-UE |
| 13 | Lu, Xiao | 2018 | 46 | 46 | 57.51±1.14 | 57.68±1.75 | I（31/15）；C（30/16） | TJ+CT | CT | —— | FMA |
| 14 | Huang, ShangJun | 2019 | 14 | 14 | 62.21±9.74 | 59.93±9.96 | I（12/2）；C（10/4） | TJ | CT | —— | FMA-LE |
| 15 | Liu, Liqun | 2019 | 48 | 48 | 64.82±7.16 | 64.18±7.59 | I（28/20）；C（26/22） | BDJ | CT | 12W | MBI、BBS、FMA |
| 16 | Xie, Beiqing | 2019 | 20 | 20 | 51.10±12.92 | 53.95±13.00 | I（13/7）；C（12/8） | BDJ+CT | CT | 3W,5T/W | FMA、BI、BBS |
| 17 | Zhu, Xiankun | 2020 | 30 | 30 | 59.1 | 58.9 | I（13/17）；C（16/14） | WQX+CT | CT | 4W,5T/W | BI、BBS |
| 18 | Fan, Jing | 2020 | 43 | 43 | 63.4±5.0 | 63.8±5.3 | I（29/14）；C（30/13） | TJ+CT | CT | 12W,3T/W | BBS、FMA-LE |
| 19 | Niu, Lulu | 2020 | 40 | 40 | 62.75±5.33 | 63.50±5.10 | I（22/18）；C（21/19） | BDJ+CT | CT | 8W | BBS、FMA |
| 20 | Wang, Jianping | 2020 | 30 | 30 | 55.10±6.28 | 55.97±6.21 | I（16/14）；C（17/13） | BDJ+CT | CT | 4W,5T/W | FMA、MBI |
| 21 | Yang, Keqin | 2020 | 30 | 30 | 64.03±3.88 | 62.93±4.67 | I（18/12）；C（14/16） | TJ+CT | CT | 6W,2T/W | BI、FMA |
| 22 | Yu, Lin | 2020 | 38 | 38 | 72.34±10.56 | 71.25±10.43 | I（21/17）；C（23/15） | BDJ+CT | CT | —— | BBS |
| 23 | Zheng, Haiying | 2020 | 37 | 37 | 47.52±13.83 | 45.49±12.15 | I（27/10）；C（29/8） | TJ+CT | CT | 12W | BBS、FMA |
| 24 | Rhayun Song | 2021 | 18 | 16 | 58.72±17.13 | 57.18±10.65 | I（10/8）；C（11/5） | TJ | CT | 24W,2T/W | MBI、BBS |
| 25 | Wang, Xinyuan | 2021 | 30 | 30 | 73.3±8.3 | 73.6±9.4 | I（22/8）；C（23/7） | TJ+CT | CT | 40D,5T/W | MBI |
| 26 | Xiao, Honglan | 2021 | 35 | 25 | 62.10±2.36 | 62.15±2.34 | I（22/13）；C（16/9） | TJ+CT | CT | —— | BBS、FMA |
| 27 | Zhang, Lingling | 2021 | 41 | 41 | 71.29±4.51 | | I（23/18）；C（24/17） | BDJ+CT | CT | 8W | BI、FMA-UE |
| 28 | Zhou, Haiying | 2021 | 35 | 35 | 69.1±8.5 | 69.5±8.3 | I（20/15）；C（22/13） | BDJ+CT | CT | 12W | FMA |
| 29 | Ye, M | 2022 | 24 | 24 | 61.63±9.21 | 62.75±6.41 | I（19/5）；C（22/2） | BDJ | CT | 24W,3T/W | FMA、BBS |
| 30 | Chen, Jun | 2022 | 46 | 46 | 52.48±4.57 | 53.12±3.97 | I（31/15）；C（31/15） | TJ+CT | CT | —— | FMA、BI |
| 31 | He, Jing | 2022 | 29 | 26 | 62.96±8.98 | 62.50±10.73 | I（27/2）；C（20/6） | TJ+CT | CT | 4W,4T/W | BBS、FMA |
| 32 | Ji, Xiaoyu | 2022 | 45 | 45 | 69.32±5.94 | 68.42±6.85 | I（26/19）；C（29/16） | BDJ+CT | CT | 8W | FMA |
| 33 | Liu, Wan | 2022 | 23 | 23 | 59.19±4.69 | 57.73±5.55 | —— | BDJ+CT | CT | 4W,6T/W | BBS |
| 34 | Tang, Qiang | 2022 | 33 | 34 | 54.9±13.1 | 56.5±11.2 | I（21/12）；C（20/14） | TJ+CT | CT | 8W,5T/W | FMA-LE、BBS、MBI |
| 35 | Xu, Jin | 2022 | 36 | 38 | 60.48±7.89 | 61.78±8.06 | I（19/17）；C（22/16） | YJJ | CT | 12W,6T/W | MBI、FMA、BBS |
| 36 | Cong, Hongzheng | 2023 | 20 | 20 | 56.1±7.59 | 53.6±5.4 | I（14/6）；C（12/8） | YJJ+CT | CT | 4W,5T/W | FMA-UE、MBI |
| 37 | Guan, Feng | 2023 | 50 | 50 | 70.07±6.45 | 70.19±6.52 | I（29/21）；C（28/22） | BDJ+CT | CT | 12W,14T/W | MBI、FMA、BBS |
| 38 | Wang, Wuhao | 2023 | 9 | 8 | 49.11±11.85 | 52.88±11.79 | I（7/2）；C（6/2） | TJ+CT | CT | 4W,7T/W | FMA-UE、MBI |
| 39 | Xu, Lin | 2023 | 30 | 30 | 64.29±3.51 | 65.31±3.62 | I（18/12）；C（16/14） | YJJ+CT | CT | 12W | BBS、MBI |
| 40 | Zhang, Fenglin | 2023 | 32 | 28 | 60.21±9.02 | 60.25±9.03 | I（19/13）；C（15/13） | WQX+CT | CT | 12W,5T/W | BBS |
| 41 | Che, Pei | 2024 | 26 | 25 | 62.04±10.26 | 65.07±9.82 | —— | WQX+CT | CT | 4W,5T/W | BBS |
| 42 | Chen, Junwen | 2024 | 21 | 21 | 52.86±14.84 | 54.14±12.30 | I（17/4）；C（17/4） | BDJ+CT | CT | 4W,5T/W | FMA-UE、BBS、MBI |
| 43 | Lai, Jiangong | 2024 | 18 | 18 | 59.00±9.17 | 58.39±9.42 | I（12/6）；C（11/7） | TJ+CT | CT | 8W,3T/W | BBS |
| 44 | Xue, Xiaoxu | 2024 | 20 | 20 | 52.95±8.38 | 57.40±9.16 | I（7/13）；C（7/13） | YJJ+CT | CT | —— | BBS |
| 45 | Xing, Xueliang | 2024 | 45 | 45 | 59.17±4.89 | 60.52±5.31 | I（27/18）；C（30/15） | YJJ+CT | CT | 8W,5T/W | FMA |
| 46 | Gong, Liuqian | 2024 | 20 | 20 | 59.50±8.56 | 57.95±7.60 | I（15/5）；C（11/9） | YJJ+CT | CT | 4W,5T/W | BBS、MBI |

Note: I, Intervention group; C, control group; D, days; W, weeks; T/W, sessions per week.
